# Pan-cancer analysis reveals signal transducer and activator of transcription (STAT) gene family as biomarkers for prognostic prediction and therapeutic guidance

**DOI:** 10.3389/fgene.2023.1120500

**Published:** 2023-03-09

**Authors:** Mei Cheng, Yifan Liu, Yangkun Guo, Man Li, Shuyuan Xian, Hengwei Qin, Yiting Yang, Weijin Qian, Jieling Tang, Yuwei Lu, Yuntao Yao, Mengyi Zhang, Minghao Jin, Long Xu, Runzhi Huang, Dayuan Xu

**Affiliations:** ^1^ Research Unit of key techniques for treatment of burns and combined burns and trauma injury, Department of Burn Surgery, The First Affiliated Hospital of Naval Medical University, Chinese Academy of Medical Sciences, Shanghai, China; ^2^ Department of Nephrology, The First Affiliated Hospital of Naval Medical University, Shanghai, China; ^3^ Shanghai Jiao Tong University School of Medicine, Shanghai, China; ^4^ Department of Orthopedics, The First Affiliated Hospital of Zhengzhou University, Zhengzhou, China; ^5^ Tongji University School of Medicine, Shanghai, China

**Keywords:** signal transducer and activator of transcription (STAT), pan-cancer, multidimensional analyses, clinical relevance, immune subtype, tumor stemness, tumor purity

## Abstract

**Background:** The signal transducer and activator of transcription (STAT) gene family have been widely found to regulate cell proliferation, differentiation, apoptosis, and angiogenesis through complex signaling pathways, and thus impacting tumor formation and development in different types of tumor. However, the roles of STATs on prognostic prediction and therapeutic guidance in pan-cancer remain unexplored.

**Materials and Methods:** The dataset of 33 types of TCGA tumor, para-carcinoma and normal tissues, was obtained from the UCSC Xena database, including the gene expression profiles in the formats of FPKM value, demographic characteristics, clinical information, and survival data of STATs. Differential expression and co-expression analyses, WGCNA, clinical relevance analysis, immune subtype analysis, tumor stemness analysis, tumor purity analysis, immune infiltration analysis, immunotherapy related analysis, tumor mutation related analysis, and drug sensitivity analysis were performed by R software.

**Results:** Differential expression of STAT1 was found between normal and BRCA tissues (*p* < 0.001, log2FC = 0.895). Additionally, the strongest correlation among STATs lied between STAT1 and STAT2 (correlation coefficient = 0.6). Moreover, high expression levels of STAT1 (*p* = 0.031) were revealed to be notably correlated with poor prognosis in KIRP. In addition, STAT1 expressed the highest value in immune subtypes C1, C2, C3, and C6 in LUAD. What’s more, strong negative correlations were demonstrated between expression of STAT6 and mDNAss and mRNAss of TGCT. Additionally, STAT4 expression was characterized to be significantly negatively correlated with tumor purity of the majority of cancer types. Moreover, STAT1 and STAT3 were shown to be generally high-expressed in pan-cancer myeloid cells, and STATs all had positive correlation with the infiltration of the majority of immune cells. In addition, STATs were revealed to be closely linked with immunotherapy response. What’s more, STAT4 expression was identified to have a strong negative correlation with TMB value in DLBC. Last but not least, positive correlations were accessed between STAT5 and sensitivity of Nelarabine (cor = 0.600, *p* < 0.001).

**Conclusion:** In the present study, we identified STATs as biomarkers for prognostic prediction and therapeutic guidance in pan-cancer. Hopefully our findings could provide a valuable reference for future STATs research and clinical applications.

## Introduction

The signal transducer and activator of transcription (STAT) gene family (STAT1, STAT2, STAT3, STAT4, STAT5A, STAT5B, and STAT6) play a complicated and fundamental role in regulating the proliferation, differentiation, apoptosis, angiogenesis, and immune system regulation of cell processes by transcribing and translating proteins with complex structures (Haura et al., 2005; Verhoeven et al., 2020). Particularly, STAT3 and STAT5 was shown to play important roles in tumorigenesis and development more than a decade ago (Buettner et al., 2002; Yu and Jove, 2004). Another study suggested that higher expression of STAT5 indicated a better prognosis in breast cancer (Barash, 2012). Moreover, it was then discovered by Yu, H. et al. that STAT3 had potential effect in cancer progression (Yu et al., 2014).

The function sections of STAT protein are as follows: N-domain, coiled-coil domain, DNA binding domain, Src-homology (SH2) domain, and transactivation domain. And as the most conservative and important section, arginine (Arg) residue of core SH2 domain could directly combine with tyrosine (Tyr) residue under phosphorylation of other molecular (Lim and Cao, 2006; Cheon and Stark, 2009). STATs were intranuclear transcription factors, but they existed in the cytoplasm under a resting state. Upon activation, STAT molecular polymerized to form a dimer and translocated to the nucleus to take part in regulating gene expression, especially for cells with interferon (IFN) signaling molecules by binding to target DNA sequences (Sadowski et al., 1993; Wegenka et al., 1993).

Although there were so many conclusions about STATs, the specific correlation between the STAT gene family and pan-cancer was still not clear. Based on this, we performed a series of analyses with the methods of Wilcox test, K-M survival analysis, Cox proportional hazards regression, weighted gene co-expression network analysis (WGCNA), Kruskal-Wallis test, Pearson/Spearman correlation analysis, one-class logistic regression (OCLR) and estimation of stromal and immune cells in malignant tumors using expression data (ESTIMATE) scores to analyze the correlation between expression of STATs and prognosis, hallmark cancer gene sets, immune subtypes, tumor stemness, tumor purity, immune infiltration, immunotherapy, and drug sensitivity in 33 The Cancer Genome Atlas (TCGA) tumor samples.

## Materials and methods

### Data acquisition and preprocessing

The dataset of 33 types of TCGA tumor tissues, along with their para-carcinoma and normal tissues, was obtained from the UCSC Xena database (http://xena.ucsc.edu/) on 28 January 2021, including the gene expression profiles in the formats of fragments per kilobase per Million (FPKM) value, phenotypic character, and survival data of STAT1, STAT2, STAT3, STAT4, STAT5A, STAT5B, and STAT6. The demographic, neoplasm staging, and prognostic information of patients were fetched from the database simultaneously.

The 33 kinds of TCGA tumors and their abbreviations: Adrenocortical carcinoma (ACC), Bladder Urothelial Carcinoma (BLCA), Breast invasive carcinoma (BRCA), Cervical squamous cell carcinoma and endocervical adenocarcinoma (CESC), Cholangiocarcinoma (CHOL), Colon adenocarcinoma (COAD), Lymphoid Neoplasm Diffuse Large B-cell Lymphoma (DLBC), Esophageal carcinoma (ESCA), Glioblastoma multiforme (GBM), Head and Neck squamous cell carcinoma (HNSC), Kidney Chromophobe (KICH), Kidney renal clear cell carcinoma (KIRC), Kidney renal papillary cell carcinoma (KIRP), Acute Myeloid Leukemia (LAML), Brain Lower Grade Glioma (LGG), Liver hepatocellular carcinoma (LIHC), Lung adenocarcinoma (LUAD), Lung squamous cell carcinoma (LUSC), Mesothelioma (MESO), Ovarian serous cystadenocarcinoma (OV), Pancreatic adenocarcinoma (PAAD), Pheochromocytoma and Paraganglioma (PCPG), Prostate adenocarcinoma (PRAD), Rectum adenocarcinoma (READ), Sarcoma (SARC), Skin Cutaneous Melanoma (SKCM), Stomach adenocarcinoma (STAD), Testicular Germ Cell Tumors (TGCT), Thyroid carcinoma (THCA), Thymoma (THYM), Uterine Corpus Endometrial Carcinoma (UCEC), Uterine Carcinosarcoma (UCS) and Uveal Melanoma (UVM).

### Differential expression analysis

Since gene expression of tumor samples were used as a continuous variable, the boxplot was used to show the differential expression median value of STATs between tumor and normal tissues. Meanwhile, the “ggpubr” R package was applied to analyze the differentially expressed genes in Wilcox test between tumor and normal tissues. Tumor types with less than three normal samples were excluded. In addition, we obtained protein expression levels of STATs between normal and tumor tissues from the Human Protein Atlas database (https://www.proteinatlas.org) (Sjöstedt et al., 2020) for further validation. Additionally, we obtained proteomics profiles from the LinkedOmics database (http://www.linkedomics.org/) (Vasaikar et al., 2018) to investigate the correlation between expression levels of STATs and relevant proteins.

### Co-expression analysis

The “corrplot” R package was utilized to explore latent expression patterns between every two STAT genes. Values and shades of color were adopted to demonstrate the expression correlation among STAT1, STAT2, STAT3, STAT4, STAT5A, STAT5B, and STAT6 (https://CRAN.R-project.org/package). Moreover, to further explore the correlation relationship among STATs, the STRING database (https://string-db.org/) (Szklarczyk et al., 2021) was employed to construct a protein-protein interaction (PPI) network.

### Weighted gene co-expression network analysis (WGCNA)

To further explore the possible co-expression of STAT family genes and specific tumor markers. WGCNA was conducted to reveal the correlations among the expression levels of differentially expressed genes (DEGs) between tumor and para-tumor KIRC tissues, 50 hallmarks of cancer, and STATs expression by WGCNA R package (Langfelder and Horvath, 2008). The 50 Hallmark cancer gene sets were collected from the Molecular Signatures Database (MSigDB) v7.0 (https://www.gsea-msigdb.org/gsea/msigdb/index.jsp) (Liberzon et al., 2015).

A gene co-expression network was initially established to find interaction patterns among genes based on the RNA-seq profiles of DEGs by correlation analysis. Specifically, if the observation value of the DEG were continuous and conformed to a normal distribution, we would perform Pearson correlation analysis, however, if the observation value of the DEG were categorical, Spearman correlation analysis would be conducted. Through performing the power function: aij = |sij|ß (aij referred to the weighted network adjacency between gene i and gene j; sij represented the correlation coefficient between gene i and gene j; *β* ≥ 1), a weighted adjacency matrix was built. Then, the optimal soft-threshold parameter *β* = 4 was obtained by calculating scale independence and mean connectivity. Afterwards, DEGs with similar expression features were integrated into a module by applying the topological overlap method. Moreover, to enlarge the capacity of the modules, a threshold was set up to merge similar modules and ensure there were no less than 20 genes in each module. Moreover, module eigengene (ME) was calculated to indicate the gene expression profiles of each module. So as to correlate different modules to corresponding phenotypic traits, the 50 Hallmark gene sets and seven STATs were input as characteristics of interest. Gene significance (GS) was performed to encode the correlations between expression of the 50 hallmark gene sets and STATs and DEGs. Module membership (MM) was computed to estimate the correlation between ME and DEGs.

### Clinical relevance analysis

Different types of tumor patients was divided into two groups according to the median value of STATs’ expression levels. Subsequently, Kaplan-Meier (KM) survival analysis was performed to analyze the correlation between patients’ overall survival (OS), disease-free interval (DFI), progression-free interval (PFI), and disease-specific survival (DSS) and expression levels of STATs (Heagerty and Zheng, 2005). Besides, to acquire the hazard ratios (HR) of the seven STATs in 33 tumor types, univariate Cox proportional hazards regression was adopted.

Moreover, to exclude the influence of confounding factors and validate the independent correlation relationships between STATs’ expression and tumor patients’ prognosis, a prognosis prediction model was constructed in KIRC patients. Specifically, the regression coefficient of each STAT gene was determined by multivariate Cox proportional hazards regression. Subsequently, risk score was calculated by the formula: ${risk\;score}_{n}=\overset{7}{\underset{i=1}{\sum }}\beta_{i}\times {Gene}_{i}$ . In the formula, ‘n’ represented the number of the KIRC patients, Gene_i_ represented the normalized expression level of STATs and ‘β_i_’ represented the corresponding regression coefficient of each STAT. Afterwards, the samples were then classified into two risk groups, high- and low-risk groups based on the median value of the risk scores. Cross-validation by train/test split was then performed to verify the multivariate prognostic model, in which risk curves, risk scatter plots and Kaplan-Meier survival plots were employed to illustrate the differences between the high- and low-risk groups. In addition, the area under the ROC curve was applied to assess the predictive accuracy of the model employing survivalROC R package. Additionally, independent prognostic analysis was proceeded by univariate and multivariate Cox regression analyses on risk score, and confounding factors including age, gender, race, grade, M, N, and T for model correction. Last but not least, differential expression analysis of the seven STATs in different stages of tumor was also conducted to further explore their clinical relevance.

### Immune subtype analysis

It has been revealed that six immune subtypes, including wound healing (C1), IFN-γ dominant (C2), inflammatory (C3), lymphocyte depleted (C4), immunologically quiet (C5), and TGF-β dominant (C6), are of great significance in therapy and prognosis spanning multiple tumor types, which were identified by studying the dominant sample features in different types of TCGA tumor (Thorsson et al., 2018). To access the differential expression levels of STAT genes among the six immune subtypes in each tumor type so as to obtain the correlation between them, we firstly performed the Hartley’s test to check whether the samples conform to homogeneity of variance. Afterwards, we conducted the Shapiro-Wilk test to examine whether the samples conform to a normal distribution. If the samples conform to homogeneity of variance or a normal distribution, we would carry out the analysis of variance (ANOVA), and if not, we would employ the Kruskal-Wallis test to access the differential expression levels of STAT genes among the six immune subtypes in each tumor type so as to obtain the correlation between them. And eventually the Kruskal-Wallis test was utilized.

### Tumor stemness analysis

Cancer stem cells (CSCs), with the characteristics of self-renewal and tumor heterogeneity, play important roles in survival, proliferation, metastasis, and recurrence of tumors. The DNA methylation-based stemness score (mDNAss) and mRNA expression-based stemness score (mRNAss) were calculated by the one-class logistic regression (OCLR) algorithm to describe the stemness indices in 33 types of tumor (Malta et al., 2018). Subsequently, to explore the association between STAT genes and tumor stemness characteristics, we carry out correlation analysis. Specifically, if the observation values of the variable were continuous and conformed to a normal distribution, we would perform Pearson correlation analysis, however, if the observation values of the variable were categorical, Spearman correlation analysis would be conducted.

### Tumor purity analysis

The ESTIMATE algorithm, a quantitative and visual method of estimating the proportion of stromal cells and immune cells, played a significant role in speculating tumor purity and malignant degree (Yoshihara et al., 2013). We accessed ESTIMATE scores in each TCGA tumor type by identifying gene expression signatures to show normal cell proportion or tumor purity accurately. Then we performed correlation analysis between STAT genes and ESTIMATE scores in pan-cancer to identify their correlation. Specifically, if the observation values of the variable were continuous and conformed to a normal distribution, we would perform Pearson correlation analysis, however, if the observation values of the variable were categorical, Spearman correlation analysis would be conducted.

### Immune infiltration analysis

In order to find out the expression characteristics of STAT genes in tumor infiltrating immune cells, we adopted gene expression profiles of STAT genes from the pan-cancer single-cell transcriptional atlases of tumor infiltrating myeloid cells and tumor infiltrating T cells reported by Zemin, Zhang, et al. (Cheng et al., 2021; Zheng et al., 2021). Additionally, the TIMER2.0 database (http://timer.cistrome.org) was also utilized to explore the correlation of STATs expression and different immune cells infiltration (Li et al., 2020).

### Immunotherapy related analysis

Immunotherapy can bring long-lasting clinical benefits, however, only a fraction of patients respond well to it. In order to reveal different tumors’ potential of escaping from T cell-mediated immune response and provide a reference for immunotherapeutical strategy, we conducted cytotoxic T lymphocyte (CTL) infiltration and survival analyses, along with T cell dysfunction and exclusion analyses employing the Tumor Immune Dysfunction and Exclusion (TIDE) database (http://tide.dfci.harvard.edu) (Fu et al., 2020) (Jiang et al., 2018). In the TIDE database, the most confident results were obtained using STATs expression data from five cohorts, which were TCGA (endometrial carcinoma), TCGA (metastatic melanoma), GSE12417_GPL570 (acute myeloid leukemia), E-MTAB-179 (neuroblastoma), METABRIC (triple negative breast cancer). In specific, Pearson/Spearman correlation analysis was conducted between expression levels of STATs and CTL infiltration in these cohorts, which was measured by expression levels of CTL markers (CD8A, CD8B, GZMA, GZMB, and PRF1). Moreover, we classified samples into STATs high expression and STATs low expression groups based on the median expression value of each STAT gene. Subsequently, we classified samples into CTL high infiltration (CTL top) and CTL low infiltration (CTL bottom) groups based on the median CTL infiltration levels to further reveal the influence of STATs expression on CTL infiltration and prognosis of patients with tumors in Cox proportional hazards model. What’s more, a variety of cohorts of patients with diverse tumors, along with the average expression profiles of cancer-associated fibroblasts (CAFs), myeloid-derived suppressor cells (MDSCs), and the M2 subtype of tumor-associated macrophages (TAMs) were utilized to model T cell dysfunction and exclusion by TIDE prediction values (Jiang et al., 2018).

### Tumor mutation related analysis

Investigations of tumor mutation burden (TMB) performed by Lawrence, et al. showed a significant association between TMB and immunotherapeutic strategy, as well as patients’ prognosis (Lawrence et al., 2013; Samstein et al., 2019). Consequently, based on the value of somatic median mutation frequencies, the form of log10 (TMB+ 1) was utilized to illustrate TMB of the 33 types of tumor and reveal the therapeutic relevance. What’s more, an oncoprint with mutation spectrum and genetic alteration was acquired from the cBioportal database (http://www.cbioportal.org) so as to access the seven STAT genes’ general alteration and distribution status in different tumors (Cerami et al., 2012), moreover, the mutation characteristics of relevant genes on recognized signaling transduction pathways with STATs including TGFBR1, TGFBR2, ACVR2A, ACVR1B, SMAD2, SMAD3, and SMAD4 were also analyzed.

### Drug sensitivity analysis

RNA sequencing profiles of STAT genes and drug activity data were obtained from the CellMiner database (https://discover.nci.nih.gov/cellminer/) (Reinhold et al., 2012). Subsequent preprocessing of the raw data was conducted by employing the Bioconductor R package (http://www.bioconductor.org/packages/release/bioc/html/impute.html). In addition, Pearson correlation analysis was performed to access the correlation between expression levels of STAT genes and drug sensitivity. Meanwhile, the plots were ranked by *p*-value, and the higher ranking suggests a more significant correlation between genes and drug sensitivity.

### Statistical analysis

All bioinformatic analyses wereconducted with R software, version 3.6.1. A significance level of a two-sided *p*-value less than 0.05 was adopted.

## Results

### Differential expression and co-expression analysis

**FIGURE 1:**
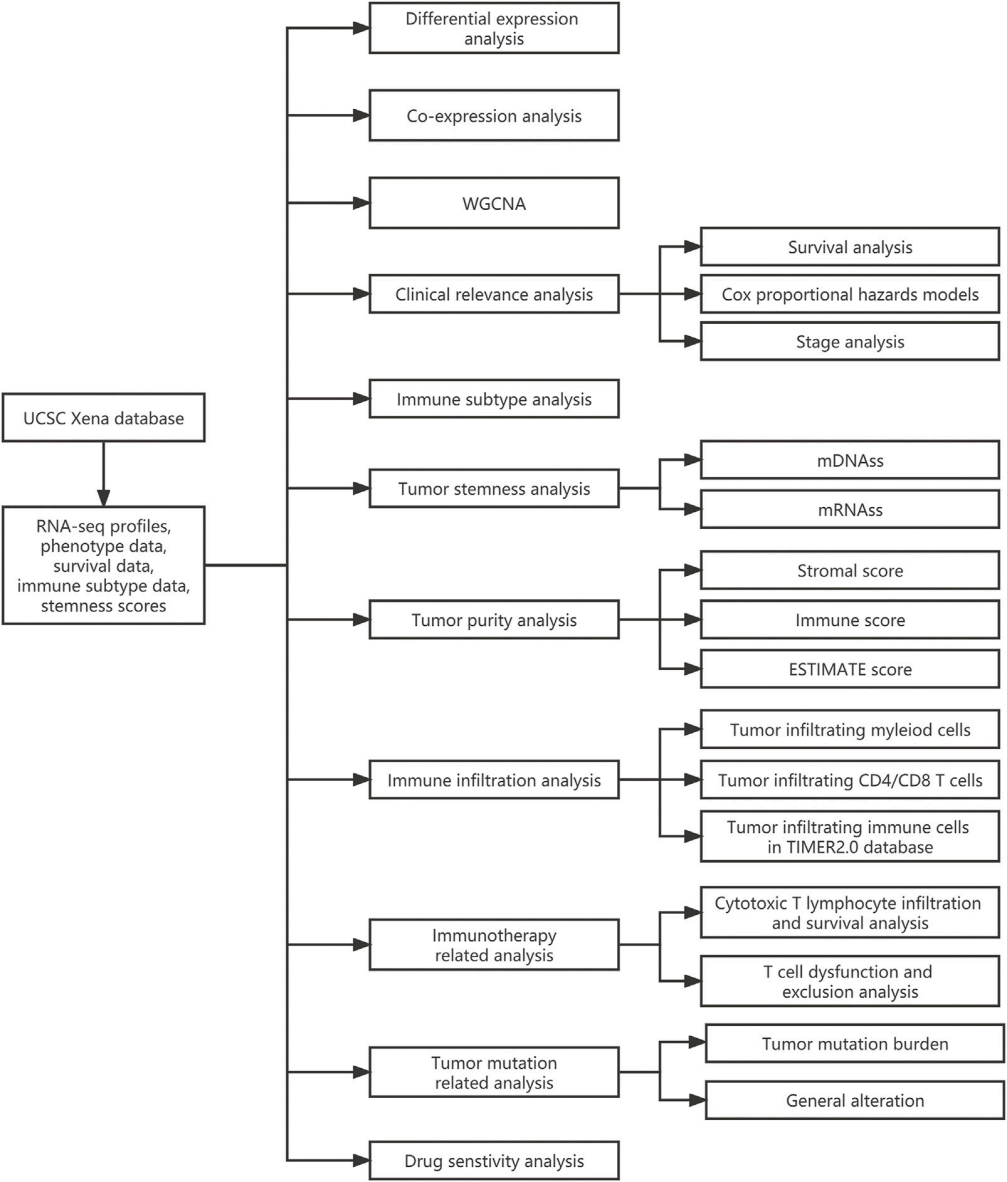
The flowchart of the whole analytic process of our study.

**FIGURE 2:**
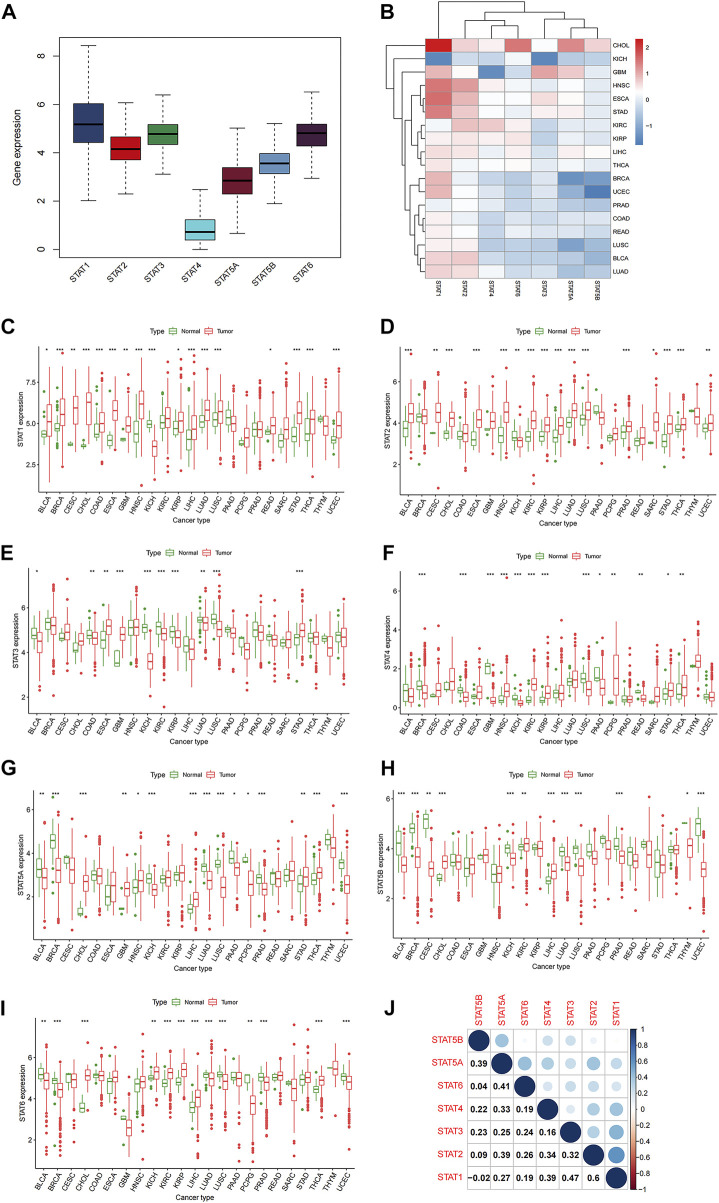
Differential expression analysis of STATs in pan-cancer. **(A)** Box plot demonstrating the average gene expression levels of STATs in all tumor types. **(B)** Heatmap revealing the expression levels of STATs measured by log2FC in different tumor types. **(C)** Box plots showing the average expression levels of STAT1 between normal and tumor tissues of 23 tumor types, in which BLCA (*p* < 0.05), BRCA (*p* < 0.001), CESC (*p* < 0.01), CHOL (*p* < 0.001), COAD (*p* < 0.001), ESCA (*p* < 0.001), GBM (*p* < 0.01), HNSC (*p* < 0.001), KICH (*p* < 0.001), KIRP (*p* < 0.05), LICH (*p* < 0.001), LUAD (*p* < 0.001), LUSC (*p* < 0.001), READ (*p* < 0.05), STAD (*p* < 0.001), THCA (*p* < 0.001), and UCEC (*p* < 0.001) exhibited statistical significance. **(D)** Box plots showing the average expression levels of STAT2 between normal and tumor tissues of 23 tumor types, in which BLCA (*p* < 0.001), CESC (*p* < 0.01), CHOL (*p* < 0.001), ESCA (*p* < 0.001), HNSC (*p* < 0.001), KICH (*p* < 0.01), KIRC (*p* < 0.001), KIRP (*p* < 0.05), LICH (*p* < 0.001), LUAD (*p* < 0.001), LUSC (*p* < 0.001), PRAD (*p* < 0.001), SARC (*p* < 0.05), STAD (*p* < 0.001), THCA (*p* < 0.001), and UCEC (*p* < 0.01) presented statistical significance. **(E)** Box plots showing the average expression levels of STAT3 between normal and tumor tissues of 23 tumor types, in which BLCA (*p* < 0.05), COAD (*p* < 0.01), ESCA (*p* < 0.01), GBM (*p* < 0.001), KICH (*p* < 0.01), KIRC (*p* < 0.001), KIRP (*p* < 0.05), LUAD (*p* < 0.01), LUSC (*p* < 0.001), and STAD (*p* < 0.001) displayed statistical significance. **(F)** Box plots showing the average expression levels of STAT4 between normal and tumor tissues of 23 tumor types, in which BRCA (*p* < 0.001), COAD (*p* < 0.001), GBM (*p* < 0.001), HNSC (*p* < 0.001), KICH (*p* < 0.01), KIRC (*p* < 0.001), KIRP (*p* < 0.05), LUSC (*p* < 0.001), PAAD (*p* < 0.05), PCPG (*p* < 0.01), READ (*p* < 0.01), STAD (*p* < 0.05), and THCA (*p* < 0.01) depicted statistical significance. **(G)** Box plots showing the average expression levels of STAT4 between normal and tumor tissues of 23 tumor types, in which BLCA (*p* < 0.01), BRCA (*p* < 0.001), CHOL (*p* < 0.001), GBM (*p* < 0.01), HNSC (*p* < 0.05), KICH (*p* < 0.001), LIHC (*p* < 0.001), LUAD (*p* < 0.001), LUSC (*p* < 0.001), PAAD (*p* < 0.05), PCPG (*p* < 0.05), PRAD (*p* < 0.001), STAD (*p* < 0.01), THCA (*p* < 0.001), and UCEC (*p* < 0.001) demonstrated statistical significance. **(H)** Box plots showing the average expression levels of STAT4 between normal and tumor tissues of 23 tumor types, in which BLCA (*p* < 0.001), BRCA (*p* < 0.001), CESC (*p* < 0.01), CHOL (*p* < 0.001), KICH (*p* < 0.001), KIRC (*p* < 0.01), LIHC (*p* < 0.001), LUAD (*p* < 0.001), LUSC (*p* < 0.001), PRAD (*p* < 0.001), THYM (*p* < 0.05), and UCEC (*p* < 0.001) revealed statistical significance. **(I)** Box plots showing the average expression levels of STAT4 between normal and tumor tissues of 23 tumor types, in which BLCA (*p* < 0.01), BRCA (*p* < 0.001), CHOL (*p* < 0.001), KICH (*p* < 0.01), KIRC (*p* < 0.001), KIRP (*p* < 0.001), LIHC (*p* < 0.001), LUAD (*p* < 0.001), LUSC (*p* < 0.001), PCPG (*p* < 0.01), PRAD (*p* < 0.001), THCA (*p* < 0.001), and UCEC (*p* < 0.001) indicated statistical significance. **(J)** Correlation analysis among STATs illustrated that STAT1 and STAT2 had the strongest correlation (correlation coefficient = 0.60).

The 33 TCGA tumor types were shown in Supplementary Table S1. And the analytical process of our present study was summarized in Figure 1. Gene expression levels of STAT family were revealed in Figure 2A, which clearly showed that STAT1, STAT2, STAT3, and STAT6 processed comparatively high expression levels, while STAT4 had the lowest expression value. STAT1 was up-regulated in most tumor types except for KICH, while STAT5B was down-regulated in almost all TCGA tumor samples except for CHOL (Figure 2B). Moreover, STAT1 was the only gene highly expressed in BRCA (*p* < 0.001, log2FC = 0.895) and also had escalated expression levels in many tumor types, such as CHOL (*p* < 0.001, log2FC = 2.327), COAD (*p* < 0.001, log2FC = 0.396), LIHC (*p* < 0.001, log2FC = 0.585) and STAD (*p* < 0.001, log2FC = 1.402) (Figure 2C). Likewise, the differences in the expression levels of STAT2 (Figure 2D), STAT3 (Figure 2E), STAT4 (Figure 2F), STAT5A (Figure 2G), STAT5B (Figure 2H), and STAT6 (Figure 2I) between normal and tumor tissues were also displayed. Additionally, employing the Human Protein Atlas database, protein expression levels of STAT1, STAT4, and STAT5A in normal and breast cancer tissues, as well as in normal and lung cancer tissues were displayed in Supplementary Figures S1A, S1B, and the protein expression levels of STAT6 in normal and lung cancer tissues, along with in normal and urothelial cancer tissues were demonstrated in Supplementary Figure S1C, which further validated the results above. Furthermore, the associated protein genes’ expression levels of STAT1 (Supplementary Figure S2A), STAT2 (Supplementary Figure S2B), STAT3 (Supplementary Figure S2C), STAT4 (Supplementary Figure S2D), STAT5A (Supplementary Figure S2E), STAT5B (Supplementary Figure S2F), and STAT6 (Supplementary Figure S2G), including positively and negatively correlated significant genes were illustrated in volcano plots and heatmaps utilizing the LinkedOmics database.

In co-expression analysis (Figure 2J), positive correlation relationships could be accessed between every two members of STAT family except for the correlation between STAT1 and STAT5B (correlation coefficient = −0.02). Moreover, it was shown that the strongest positive correlation was between STAT1 and STAT2 (correlation coefficient = 0.6), which implied a strongest interaction. Meanwhile, STAT1 and STAT3 were of a relatively high association (correlation coefficient = 0.47). In addition, STAT1 and STAT4, STAT2 and STAT5A, and STAT5A and STAT5B possessed the same correlation coefficient of 0.39. What’s more, exerting the STRING database, a PPI network was obtained to further prove the powerful association among members of STAT family in Supplementary Figure S3.

In WGCNA, the KIRC samples dendrogram and trait heatmap of the 50 hallmark gene sets and STATs were shown in Supplementary Figure S4A. Additionally, the cluster dendrogram of DEGs revealed the co-expression modules with different branches and color blocks in Figure 4B. What’s more, in the module trait relationships heatmap, the black module was demonstrated to have generally strong correlations with STATs, especially STAT1 (correlation coefficient = 0.73, *P* = 3e-84), STAT2 (correlation coefficient = 0.63, *P* = 9e-56), STAT4 (correlation coefficient = 0.7, *P* = 2e-75), and STAT5A (correlation coefficient = 0.72, *P* = 2e-80). Moreover, the black module also had strong correlations with hallmark allograft rejection (correlation coefficient = 0.78, *P* = 3e-103), interferon γ response (correlation coefficient = 0.78, *P* = 2e-103), inflammatory response (correlation coefficient = 0.6, *P* = 4e-51), interferon *a* response (correlation coefficient = 0.59, *P* = 8e-48), and IL6-JAK-STAT3 signaling (correlation coefficient = 0.47, *P* = 2e-28).

### Clinical correlation analysis

**FIGURE 3:**
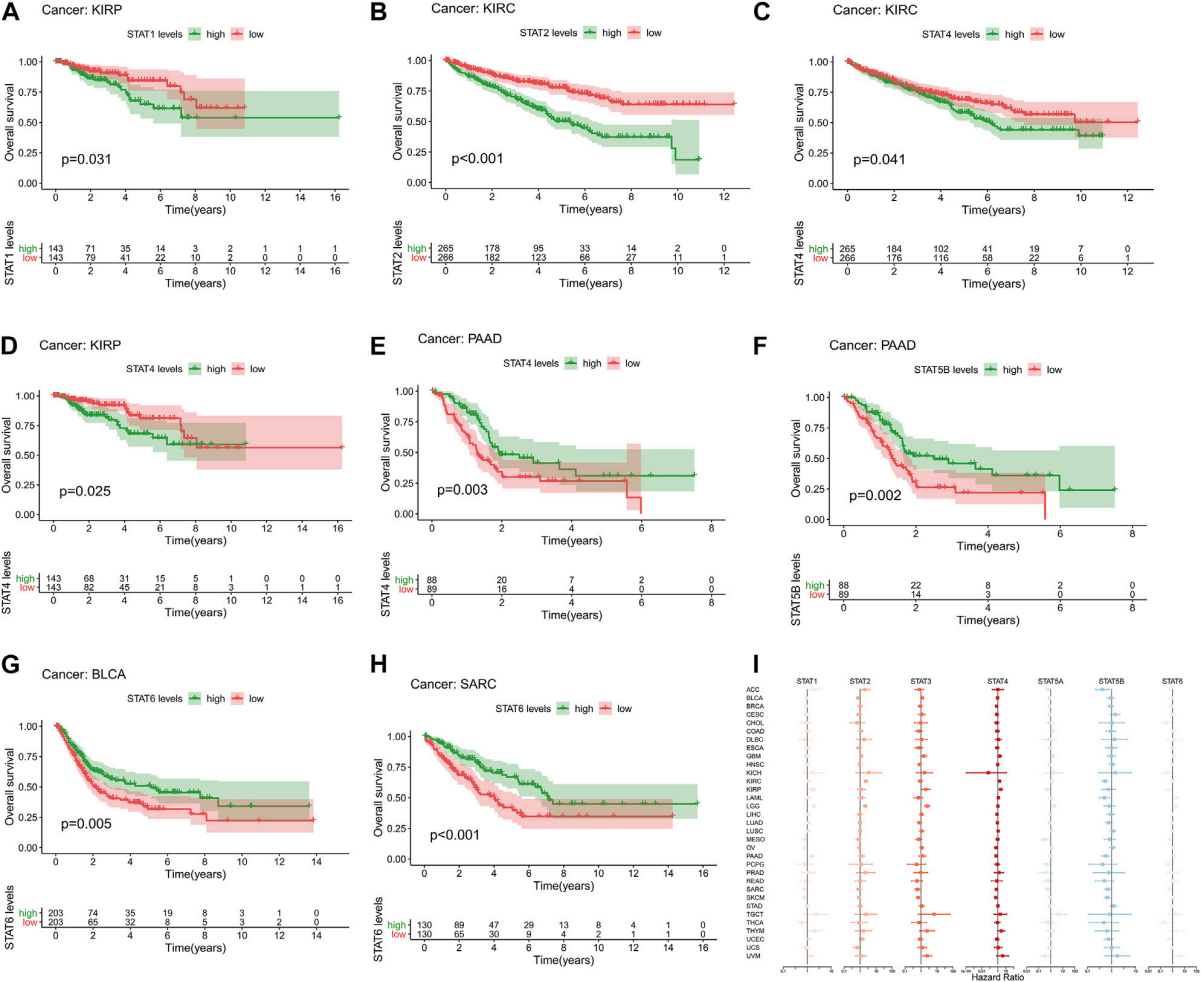
Survival analysis of STATs in pan-cancer. **(A)** Kaplan-Meier plot showed that low STAT1 expression indicated a better prognosis in KIRP patients (*p* = 0.031). **(B)** Kaplan-Meier plot revealed that low STAT2 expression suggested a more favorable prognosis in KIRC patients (*p* < 0.001). **(C)** Kaplan-Meier plot visualized that high expression levels of STAT4 were associated with worse clinical outcomes in KIRC patients (*p* = 0.041). **(D)** Kaplan-Meier plot displayed that elevated STAT4 levels were linked with more unfavorable clinical outcomes in KIRP patients (*p* = 0.025). **(E)** Kaplan-Meier plot portrayed that up-regulated STAT4 expression was correlated with better prognosis in PAAD patients (*p* = 0.003). **(F)** Kaplan-Meier plot exhibited that high STAT5B levels indicated a better overall survival status in patients with PAAD (*p* = 0.002). **(G)** Kaplan-Meier plot illustrated that down-regulation of STAT6 expression was associated with worse clinical outcomes in patients with BLCA (*p* = 0.005). **(H)** Kaplan-Meier plot characterizing that low STAT6 levels suggested a more unfavorable prognosis in patients with SARC (*p* < 0.001). **(I)** Forest plot showing the results of Cox proportional hazards regression analyses of STATs in 33 tumor types, in which STAT1 and STAT4 were identified as risk factors of KIRP with HR > 1, as well as STAT2 and STAT4 of KIRC, while STAT4 and STAT5B were recognized to be protective factors of PAAD with HR < 1, as well as STAT6 of BLCA and SARC.

High expression levels of STAT1 (*p* = 0.031, Figure 3A) were revealed to be notably correlated with poor prognosis in KIRP. Additionally, elevated expression levels of STAT2 (*p* < 0.001, Figure 3B) and STAT4 (*p* = 0.041, Figure 3C) were significantly associated with an unfavourable prognosis in KIRC. In addition, up-regulation of STAT4 (*p* = 0.025, Figure 3D) was demonstrated to be linked with poor OS in patients with KIRP. Moreover, up-regulated STAT4 expression (*p* = 0.003, Figure 3E) and STAT5B expression (*p* = 0.002, Figure 3F) were discovered to be correlated with better prognosis in patients with PAAD. Besides, it was shown that highly expressed STAT6 was significantly correlated with favorable prognosis in BLCA (*p* = 0.005, Figure 3G) and SARC (*p* < 0.001, Figure 3H). What’s more, the univariate Cox proportional hazards regression analysis results of STATs in 33 tumor types were all illustrated in Figure 3I, which were consistent with the results of the K-M survival analyses above. To be specific, STAT1 and STAT4 were manifested as risk factors of KIRP with HR > 1, as well as STAT2 and STAT4 of KIRC, while STAT4 and STAT5B were revealed to be protective factors of PAAD with HR < 1, as well as STAT6 of BLCA and SARC.

Additionally, the statistically significant results of DFI (Supplementary Figure S5A), PFI (Supplementary Figure S5B), and DSS (Supplementary Figure S5C) between high and low expression of STAT1 were also shown in K-M survival plots, as well as the univariate Cox hazards regression analysis results measured by OS (Supplementary Figure S5D), DFI (Supplementary Figure S5E), PFI (Supplementary Figure S5F), and DSS (Supplementary Figure S5G) in forest plots. In addition, expression levels of STAT3 were also revealed to be correlated with DFI (Supplementary Figure S6A), PFI (Supplementary Figure S6B), and DSS (Supplementary Figure S6C) of different tumor patients in K-M survival plots, and the univariate Cox hazards regression analysis results measured by OS (Supplementary Figure S6D), DFI (Supplementary Figure S6E), PFI (Supplementary Figure S6F), and DSS (Supplementary Figure S6G) in forest plots were as well demonstrated. Moreover, STAT6 expression was also characterized to be linked with DFI (Supplementary Figure S7A), PFI (Supplementary Figure S7B), and DSS (Supplementary Figure S7C) of patients with different tumors in K-M survival plots, plus displaying the univariate Cox hazards regression analysis results measured by OS (Supplementary Figure S7D), DFI (Supplementary Figure S7E), PFI (Supplementary Figure S7F), and DSS (Supplementary Figure S7G) in forest plots.

Furthermore, a prognostic prediction model was constructed based on the expression of seven STATs in KIRC patients by calculating risk scores. In the subsequent cross-validation by train/test split, the risk curves displayed KIRC patients of high- and low-risk groups in increasing risk score in all set, train set and test set (Supplementary Figure S8A). Additionally, risk scatter plots demonstrated sensor (alive or dead) and survival time of patients with ascending risk score in all set, train set and test set (Supplementary Figure S8B), which manifested that patients with higher risk scores had a higher mortality and possibly a shorter survival time. In addition, K-M survival plots all showed statistically significant differences (*p* < 0.001) between high- and low-risk KIRC patients in all set, train set and test set (Supplementary Figure S8C), which suggested a favorable effectiveness of the prognostic model. What’s more, ROC curves revealed a decent accuracy of the prognostic model in all set (AUC = 0.745), train set (AUC = 0.752) and test set (AUC = 0.757) (Supplementary Figure S8D). Moreover, it was shown that risk score had a HR = 14.783 (95% CI (5.652–38.664), *p* < 0.001) in univariate Cox regression (Figure 7E) and a HR = 7.204 (95% CI (2.293–22.631), *p* < 0.001) in multivariate Cox regression (Figure 7F). Consequently, we confirmed that the risk score was an independent prognostic factor for KIRC patients.

Last but not least, we discovered that STAT gene expression levels were related to tumor stages in various cancers, such as COAD (Supplementary Figure S9A) and STAD (Supplementary Figure S9B). STAT1 (*p* < 0.01) and STAT4 (*p* < 0.001) revealed the significant correlation in different stages for COAD, with both expressing the lowest in stage IV. In comparison to STAT1, STAT4 expression was lower in all tumor progression stages and on a downward trend, which could be used to monitor tumor progression and formulate therapeutic options in clinical tumor therapy. Furthermore, the expression of STAT2 (*p* < 0.001) and STAT4 (*p* < 0.05) in STAD was correlated with TNM stages, with both expressing the highest in stage III.

### Immune subtype analysis

**FIGURE 4:**
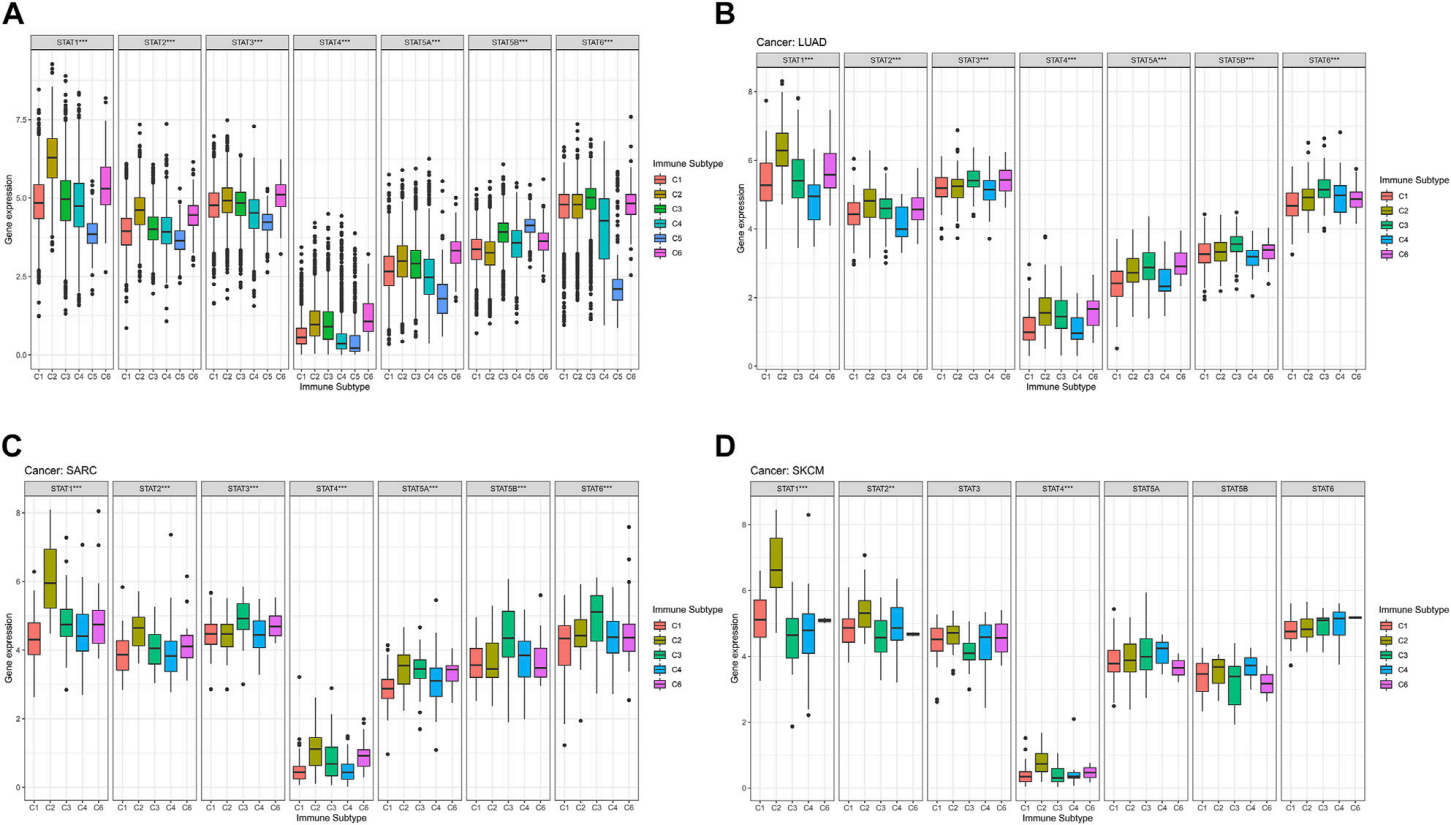
Immune subtype analysis of STATs in pan-cancer. **(A)** Box plots showed the differential expression of average expression levels of STAT1 (*p* < 0.001), STAT2 (*p* < 0.001), STAT3 (*p* < 0.001), STAT4 (*p* < 0.001), STAT5A (*p* < 0.001), STAT5B (*p* < 0.001), and STAT6 (*p* < 0.001) across TCGA cancers among C1-C6 immune subtypes. **(B)** Box plots displayed significant differences in expression levels of STAT1 (*p* < 0.001), STAT2 (*p* < 0.001), STAT3 (*p* < 0.001), STAT4 (*p* < 0.001), STAT5A (*p* < 0.001), STAT5B (*p* < 0.001), and STAT6 (*p* < 0.001) among immune subtypes C1, C2, C3, C4, and C6 in LUAD. **(C)** Box plots revealed significant differences in expression levels of STAT1 (*p* < 0.001), STAT2 (*p* < 0.001), STAT3 (*p* < 0.001), STAT4 (*p* < 0.001), STAT5A (*p* < 0.001), STAT5B (*p* < 0.001), and STAT6 (*p* < 0.001) among immune subtypes C1, C2, C3, C4, and C6 in SARC. **(D)** Box plots demonstrated significant differences in expression levels of STAT1 (*p* < 0.001), STAT2 (*p* < 0.01), and STAT4 (*p* < 0.001) among immune subtypes C1, C2, C3, C4, and C6 in SKCM.

The average expression values of STAT1 (*p* < 0.001), STAT2 (*p* < 0.001), STAT3 (*p* < 0.001), STAT4 (*p* < 0.001), STAT5A (*p* < 0.001), STAT5B (*p* < 0.001), and STAT6 (*p* < 0.001) among immune subtypes C1-C6 in all tumor types were identified to have notable differences (Figure 4A). Moreover, it was worth noting that the expression levels of STAT genes in subtype C5 were the most down-regulated of six immune subtypes except for STAT5B. Additionally, STAT4 was shown to express the lowest value among STATs in all immune subtypes.

Specifically, in LUAD, significant differences were demonstrated in the expression levels of STAT1 (*p* < 0.001), STAT2 (*p* < 0.001), STAT3 (*p* < 0.001), STAT4 (*p* < 0.001), STAT5A (*p* < 0.001), STAT5B (*p* < 0.001), and STAT6 (*p* < 0.001) among immune subtypes C1, C2, C3, C4, and C6 (Figure 4B). In addition, STAT1 expressed the highest value in immune subtypes C1, C2, C3, and C6, while expression level of STAT4 was the lowest in immune subtypes C1, C2, C3, C4, and C6. Besides, C2 and C3 possessed comparatively elevated expression of STAT1, STAT2, STAT3, STAT4, STAT5A, STAT5B, and STAT6 compared with other immune subtypes.

In terms of SARC, it was illustrated that expression levels of STAT1 (*p* < 0.001), STAT2 (*p* < 0.001), STAT3 (*p* < 0.001), STAT4 (*p* < 0.001), STAT5A (*p* < 0.001), STAT5B (*p* < 0.001), and STAT6 (*p* < 0.001) among immune subtypes C1, C2, C3, C4, and C6 were of significant differences (Figure 4C). Moreover, escalated expression of STAT1, STAT2, STAT4, STAT5A were found in immune subtype C2. In addition, immune subtype C3 was identified to have up-regulated expression of STAT3, STAT5B, and STAT6. What’s more, expression level of STAT4 was the most down-regulated of all STAT genes in immune subtypes C1, C2, C3, C4, and C6.

For SKCM, there were also notable differences in the expression levels of STAT1 (*p* < 0.001), STAT2 (*p* < 0.01), and STAT4 (*p* < 0.001) among immune subtypes C1, C2, C3, C4, and C6 (Figure 4D). Additionally, C2 had the highest expression of STAT1, STAT2, STAT3, and STAT4. Likewise, STAT4 expression had the most down-regulation of all STAT genes in immune subtypes C1, C2, C3, C4, and C6.

### Tumor stemness analysis

**FIGURE 5:**
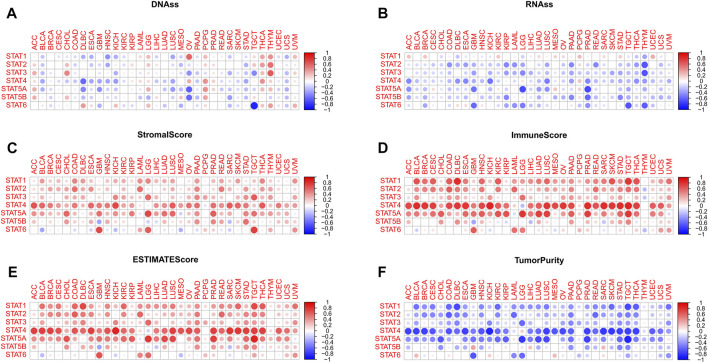
Tumor stemness and tumor purity analyses of STATs in pan-cancer. **(A)** Heatmap revealing the correlations between expression levels of STAT1, STAT2, STAT3, STAT4, STAT5A, STAT5B and STAT6 and mDNAss in 33 TCGA tumor types. **(B)** Heatmap showling the correlations between expression levels of STAT1, STAT2, STAT3, STAT4, STAT5A, STAT5B and STAT6 and mRNAss in 33 TCGA tumor types. **(C)** Heatmap demonstrating the correlations between expression levels of STAT1, STAT2, STAT3, STAT4, STAT5A, STAT5B and STAT6 and stromal score. **(D)** Heatmap demonstrating the correlations between expression levels of STAT1, STAT2, STAT3, STAT4, STAT5A, STAT5B, and STAT6 and immune score. **(E)** Heatmap demonstrating the correlations between expression levels of STAT1, STAT2, STAT3, STAT4, STAT5A, STAT5B, and STAT6 and ESTIMATE score. **(F)** Heatmap demonstrating the correlations between expression levels of STAT1, STAT2, STAT3, STAT4, STAT5A, STAT5B and STAT6 and tumor purity. (mDNAss: DNA methylation-based stemness score; mRNAss: mRNA-based stemness score).

Positive and negative correlations between STAT genes and mDNAss of different tumor types were exhibited in Figure 5A. For instance, strong negative correlations were found between STAT4 and DLBC, STAT5A and OV, STAT5B and OV, and STAT6 and TGCT. Instead, positive correlations existed between STAT1 and OV, STAT2 and THYM, and STAT2 and THYM.

For mRNAss, a number of negative correlations between STATs and TCGA tumor types were shown in Figure 5B, except for STAT1. Take PRAD, for example, negative correlations of STAT4, STAT5A, STAT5B, and STAT6 suggested that the higher expression of STATs, the higher differentiation and lower malignant degree of tumor cells with less tumor stemness characteristics, which might aid in speculating tumor prognosis in clinical application.

### Tumor purity analysis

**FIGURE 6:**
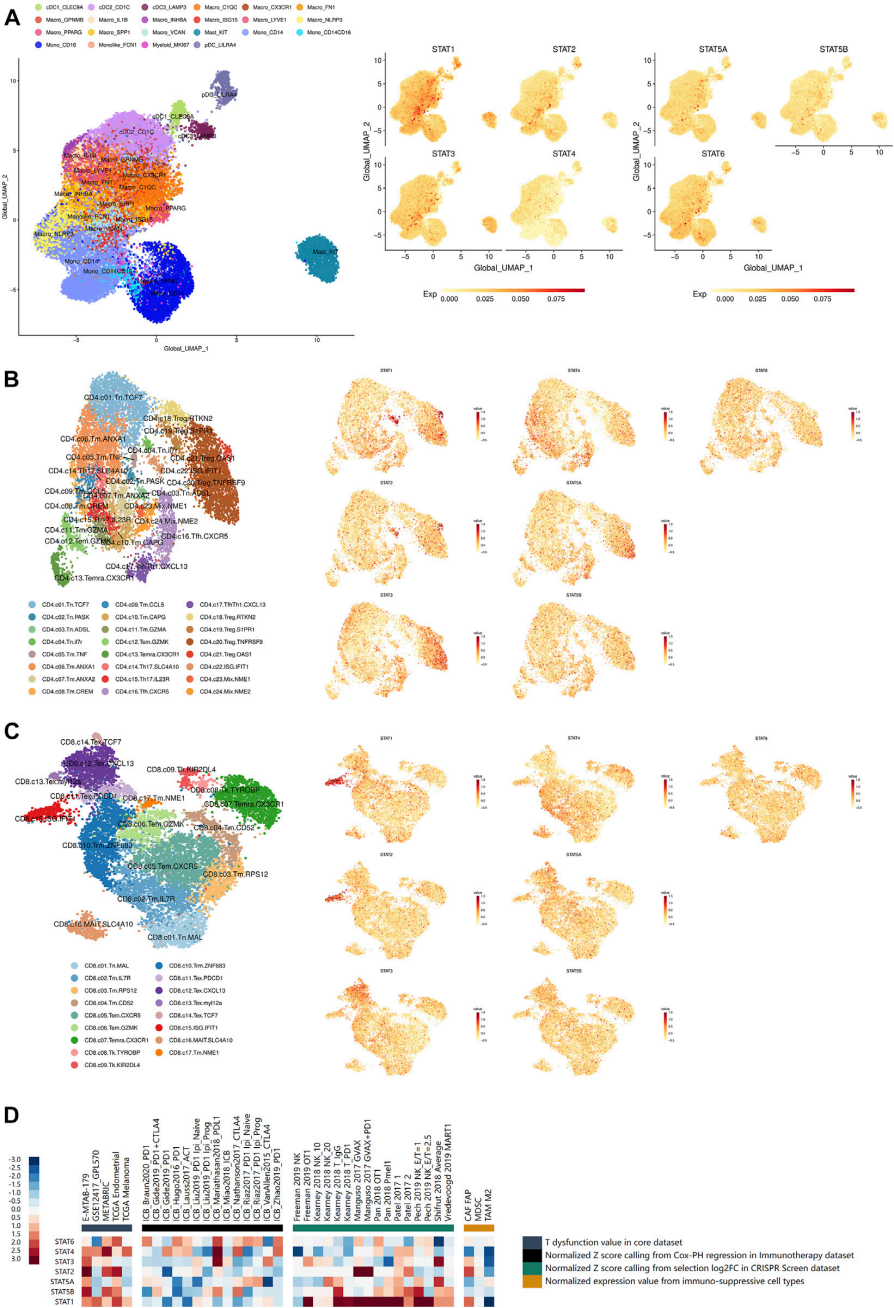
Immune infiltration and immunotherapy related analyses of STATs in pan-cancer. **(A)** UMAP feature plots depicting the distribution of STAT1, STAT2, STAT3, STAT4, STAT5A, STAT5B, and STAT6 expression in myeloid cells in pan-cancer, which showed that STAT1 was remarkably high expressed in macrophages with marker ISG15, while STAT3 expression was significantly escalated in mast cells with marker KIT. **(B)** UMAP feature plots presented the distribution of STAT1, STAT2, STAT3, STAT4, STAT5A, STAT5B, and STAT6 expression in CD4^+^ T cells in pan-cancer, which revealed that STAT1 and STAT2 expression were notably up-regulated in CD4^+^ Treg cell with marker OSA1 and CD4^+^ T cell with ISG IFIT1. **(C)** UMAP feature plots exhibited the distribution of STAT1, STAT2, STAT3, STAT4, STAT5A, STAT5B, and STAT6 expression in CD8^+^ T cells in pan-cancer, which illustrated that STAT1 and STAT2 had prominent up-regulation in CD8^+^ T cell with ISG IFIT1. **(D)** Heatmap visualizing the correlation analysis between STATs expression and tumor immune dysfunction and exclusion-related factors including T dysfunction value in core dataset, normalized z score calling from Cox-PH regression in immunotherapy dataset, normalized z score calling from selection log2FC in CRISPR Screen dataset, and normalized expression value from immune-suppressive cell types.

According to Figure 5C; Figure 6E, it was characterized that almost every type of tumor had obviously positive correlations between expression of STAT genes and stromal score, immune score, and ESTIMATE score, which indicated that higher the expression of STAT genes, the more stromal and immune cells in the TME, and thus the less tumor purity (Figure 5F). Notably, STAT4 had the strongest correlations across the majority of TCGA tumor types. On the contrary, negative correlations existed in only several tumor types, such as BLCA and STAT6, GBM and STAT5B, and SARC and STAT5B.

More specifically, for BRCA (Supplementary Figure S10A), negative correlations were discovered between STAT2 (R = −0.11, *p* < 0.001), STAT3 (R = −0.17, *p* < 0.001), STAT4 (R = −0.18, *p* < 0.001), STAT5A (R = −0.26, *p* < 0.001), STAT5B (R = −0.32, *p* < 0.001), and STAT6 (R = −0.36, *p* < 0.001) and mRNAss, while STAT1 (R = 0.25, *p* < 0.001) had a positive correlation with mRNAss. Additionally, there were also negative correlations between STAT3 (R = −0.092, *p* = 0.011), STAT4 (R = −0.07, *p* = 0.049), STAT5A (R = −0.14, *p* < 0.001), STAT5B (R = −0.16, *p* < 0.001), and STAT6 (R = −0.16, *p* < 0.001) and mDNAss. Consequently, we could conclude that the higher expression of STAT3, STAT4, STAT5A, STAT5B, and STAT6, the higher differentiation degree and less tumor stemness characteristics of BRCA tumor cells. Moreover, the expression of STAT1 had prominently positive correlations with stromal score (R = 0.21, *p* < 0.001), immune score (R = 0.52, *p* < 0.001), and ESTIMATE score (R = 0.44, *p* < 0.001), same as STAT2 (stromal score: R = 0.36, *p* < 0.001; Immune score: R = 0.47, *p* < 0.001; and ESTIMATE score: R = 0.49, *p* < 0.001), STAT4 (stromal score: R = 0.51, *p* < 0.001; Immune score: R = 0.80, *p* < 0.001; and ESTIMATE score: R = 0.76, *p* < 0.001) and STAT5A (stromal score: R = 0.29, *p* < 0.001; Immune score: R = 0.41, *p* < 0.001; and ESTIMATE score: R = 0.41, *p* < 0.001). Consequently, we deduced that high expression of STAT1, STAT2, STAT4, and STAT5A indicated low BRCA tumor purity.

For COAD (Supplementary Figure S10B), negative correlations were also identified between STAT1 (R = −0.17, *p* < 0.001), STAT2 (R = −0.50, *p* < 0.001), STAT3 (R = −0.28, *p* < 0.001), STAT4 (R = −0.36, *p* < 0.001), STAT5A (R = −0.28, *p* < 0.001), and STAT5B (R = −0.26, *p* < 0.001) and mRNAss. In addition, there were also negative correlations between STAT1 (R = −0.12, *p* = 0.043), STAT2 (R = −0.18, *p* < 0.001), and STAT4 (R = −0.21, *p* < 0.001) and mDNAss. Hence, we proposed that higher STAT1, STAT2, and STAT4 expression suggested less tumor stemness characteristics. Besides, the expression of STAT1 was exhibited to have significantly positive correlations with stromal score (R = 0.49, *p* < 0.001), immune score (R = 0.66, *p* < 0.001), and ESTIMATE score (R = 0.60, *p* < 0.001), as well as STAT2 (stromal score: R = 0.58, *p* < 0.001; Immune score: R = 0.65, *p* < 0.001; and ESTIMATE score: R = 0.65, *p* < 0.001), STAT3 (stromal score: R = 0.32, *p* < 0.001; Immune score: R = 0.44, *p* < 0.001; and ESTIMATE score: R = 0.40, *p* < 0.001), STAT4 (stromal score: R = 0.53, *p* < 0.001; Immune score: R = 0.75, *p* < 0.001; and ESTIMATE score: R = 0.67, *p* < 0.001), STAT5A (stromal score: R = 0.33, *p* < 0.001; immune score: R = 0.39, *p* < 0.001; and ESTIMATE score: R = 0.38, *p* < 0.001), and STAT5B (stromal score: R = 0.27, *p* < 0.001; and ESTIMATE score: R = 0.18, *p* < 0.001). Thus, higher the expression of STAT1, STAT2, STAT3, STAT4, STAT5A, and STAT5B, the lower the tumor purity.

For HNSC (Supplementary Figure S10C), negative correlations were spotted between STAT2 (R = −0.16, *p* < 0.001), STAT4 (R = −0.26, *p* < 0.001), and STAT5B (R = −0.24, *p* < 0.001) and RNAss, while STAT6 (R = 0.11, *p* = 0.018) had a positive correlation with RNAss. Besides, there were also negative correlations between STAT1 (R = −0.41, *p* = 0.011), STAT2 (R = −0.32, *p* = 0.049), STAT4 (R = −0.34, *p* < 0.001), STAT5A (R = −0.17, *p* < 0.001), and STAT5B (R = −0.14, *p* = 0.0017) and mDNAss. Taken together, it was implied that up-regulation of STAT2, STAT4, and STAT5B meant down-regulation of HNSC tumor stemness characteristics. Moreover, the expression of STAT1 was depicted to possess significantly positive correlations with stromal score (R = 0.23, *p* < 0.001), immune score (R = 0.58, *p* < 0.001), and ESTIMATE score (R = 0.46, *p* < 0.001), as well as STAT2 (stromal score: R = 0.24, *p* < 0.001; Immune score: R = 0.48, *p* < 0.001; and ESTIMATE score: R = 0.41, *p* < 0.001), STAT3 (immune score: R = 0.22, *p* < 0.001; and ESTIMATE score: R = 0.17, *p* < 0.001), STAT4 (stromal score: R = 0.50, *p* < 0.001; Immune score: R = 0.70, *p* < 0.001; and ESTIMATE score: R = 0.69, *p* < 0.001), STAT5A (stromal score: R = 0.27, *p* < 0.001; Immune score: R = 0.61, *p* < 0.001; and ESTIMATE score: R = 0.52, *p* < 0.001), and STAT5B (stromal score: R = 0.32, *p* < 0.001; Immune score: R = 0.25, *p* < 0.001; and ESTIMATE score: R = 0.33, *p* < 0.001). On the whole, it was illustrated that higher the expression of STAT1, STAT2, STAT3, STAT4, STAT5A, and STAT5B, the lower the tumor purity.

For LIHC (Supplementary Figure S10D), negative correlations were recognized between STAT3 (R = −0.29, *p* < 0.001), STAT4 (R = −0.14, *p* < 0.001), STAT5A (R = −0.17, *p* = 0.0013), STAT5B (R = −0.12, *p* = 0.026), and STAT5A (R = −0.21, *p* < 0.001) and mRNAss. Additionally, there were also negative correlations between STAT1 (R = −0.17, *p* = 0.0011), STAT4 (R = −0.22, *p* < 0.001) and mDNAss, while STAT2 (R = 0.10, *p* = 0.048) and STAT5A (R = 0.13, *p* = 0.011) had a positive correlation with mDNAss. All in all, up-regulated expression of STAT4 was revealed to suggest down-regulated LIHC tumor stemness characteristics. What’s more, the expression of STAT1 was visualized to possess significantly positive correlations with stromal score (R = 0.28, *p* < 0.001), immune score (R = 0.43, *p* < 0.001), and ESTIMATE score (R = 0.40, *p* < 0.001), plus STAT2 (immune score: R = 0.16, *p* = 0.0018; and ESTIMATE score: R = 0.13, *p* = 0.015), STAT3 (stromal score: R = 0.27, *p* < 0.001; immune score: R = 0.22, *p* < 0.001; and ESTIMATE score: R = 0.26, *p* < 0.001), STAT4 (stromal score: R = 0.40, *p* < 0.001; immune score: R = 0.54, *p* < 0.001; and ESTIMATE score: R = 0.52, *p* < 0.001), and STAT5A (stromal score: R = 0.38, *p* < 0.001; Immune score: R = 0.52, *p* < 0.001; and ESTIMATE score: R = 0.50, *p* < 0.001), while STAT5B (stromal score: R = −0.14, *p* = 0.0063; Immune score: R = −0.26, *p* < 0.001; and ESTIMATE score: R = −0.23, *p* < 0.001) had negative correlations. In general, it was displayed that higher expression of STAT1, STAT2, STAT3, STAT4, and STAT5A indicated a lower tumor purity.

### Immune infiltration analysis

The distribution of STAT1, STAT2, STAT3, STAT4, STAT5A, STAT5B, and STAT6 expression in myeloid cells in pan-cancer single-cell transcriptional atlases of tumor infiltrating myeloid cells and tumor infiltrating T cells was demonstrated in Figure 6A, Supplementary Figures S11A–G, which revealed that STAT1 and STAT3 were generally high-expressed in myeloid cells, while STAT4 and STAT5B had basically no expression. In particular, STAT1 was remarkably high expressed in macrophages with marker IFN stimulated gene (ISG) 15, while STAT3 expression was significantly escalated in mast cells with marker KIT. Additionally, the distribution of STAT1, STAT2, STAT3, STAT4, STAT5A, STAT5B, and STAT6 expression in CD4^+^ T cells in pan-cancer were also clearly illustrated (Figure 6B; Supplementary Figures S11A–G), which showed that STAT1 and STAT2 expression were notably up-regulated in CD4^+^ Treg cell with marker OSA1 and CD4^+^ T cell with ISG IFIT1. In addition, the distribution of STAT1, STAT2, STAT3, STAT4, STAT5A, STAT5B, and STAT6 expression in CD8^+^ T cells in pan-cancer were displayed (Figure 6B; Supplementary Figures S11A–G), which presented that STAT1 and STAT2 had prominent up-regulation in CD8^+^ T cell with ISG IFIT1. Moreover, STAT1, STAT2, STAT3, STAT4, STAT5A, STAT5B, and STAT6 expression were all shown to be positively correlated with the infiltration of the majority of immune cells including dendritic cells, monocytes, macrophages, CD4^+^ T cells, CD8 T cells, and B cells, in which STAT1, STAT2, STAT4, and STAT5A were the most significant exerting theTIMER2.0 database (Supplementary Figures S14A–D; S15A–C).

### Immunotherapy related analysis

The statistically significant results of correlation between STATs expression and CTL infiltration levels were all visualized. As shown in Supplementary Figure S14A, STAT1 expression was revealed to be positively correlated with CTL infiltration levels in endometrial carcinoma (r = 0.363, *p* = 2.48e^−18^), acute myeloid leukemia (r = 0.335, *p* = 2.53e^−3^), neuroblastoma (r = 0.363, *p* = 1.52e^−13^), metastatic melanoma (r = 0.623, *p* = 1.69e^−35^), and triple negative breast cancer (r = 0.655, *p* = 6.97e^−30^). Besides, as demonstrated in Supplementary Figure S16b, STAT2 expression was depicted to had positive correlation CTL infiltration levels in endometrial carcinoma (r = 0.192, *p* = 6.61e^−6^), neuroblastoma (r = 0.229, *p* = 5.22e^−6^), metastatic melanoma (r = 0.356, *p* = 6.28e^−11^), and triple negative breast cancer (r = 0.360, *p* = 1.58e^−8^). In addition, as shown in Supplementary Figure S16C, STAT3 expression was revealed to be positively associated with CTL infiltration levels in neuroblastoma (r = 0.201, *p* = 6.36e^−5^) and metastatic melanoma (r = 0.231, *p* = 3.27e^−5^). Moreover, as demonstrated in Supplementary Figure S16D, STAT4 expression was characterized to had positive correlation with CTL infiltration levels in endometrial carcinoma (r = 0.584, *p* = 1.01e^−50^), acute myeloid leukemia (r = 0.418, *p* = 1.25e^−4^), neuroblastoma (r = 0.579, *p* = 3.52e^−36^), metastatic melanoma (r = 0.540, *p* = 2.37e^−25^), and triple negative breast cancer (r = 0.835, *p* = 8.30e^−62^). What’s more, as shown in Supplementary Figure S16E, STAT5A expression was characterized to be positively linked with CTL infiltration levels in endometrial carcinoma (r = 0.298, *p* = 1.55e^−12^), acute myeloid leukemia (r = 0.220, *p* = 7.84e^−5^), neuroblastoma (r = 0.578, *p* = 4.53e^−36^), and triple negative breast cancer (r = 0.467, *p* = 5.25e^−14^). Additionally, as demonstrated in Supplementary Figure S16F, STAT5B expression was displayed to be positively correlated with CTL infiltration levels in endometrial carcinoma (r = 0.106, *p* = 1.40e^−2^), acute myeloid leukemia (r = 0.291, *p* = 1.28e^−7^), and triple negative breast cancer (r = 0.327, *p* = 3.31e^−7^). Moreover, as shown in Supplementary Figure S16G, STAT6 expression was shown to be positively linked with CTL infiltration levels in neuroblastoma (r = 0.478, *p* = 1.32e^−23^) and triple negative breast cancer (r = 0.253, *p* = 9.26e^−5^). In a word, higher expression of STATs all suggested higher level of CTL infiltrations.

The statistically significant results of the survival analysis of STATs expression and CTL infiltration levels in different tumors were clearly illustrated. In endometrial carcinoma (z score = 2.07, *p* = 0.0383), a higher CTL level indicated a better prognosis when STAT1 had relatively low expression (Supplementary Figure S17A). Moreover, in neuroblastoma (z score = 3.22, *p* = 0.0013), endometrial carcinoma (z score = 2.49, *p* = 0.0127), and triple negative breast cancer (z score = 2.05, *p* = 0.04), higher CTL levels all suggested more favorable survival outcomes when STAT2 had relatively low expression (Supplementary Figures S17B–D). Additionally, in triple negative breast cancer (z score = 2.68, *p* = 0.00731), a lower CTL level indicated a better survival outcome when STAT4 were high expressed (Supplementary Figure S17E). In addition, in neuroblastoma (z score = 2.46, *p* = 0.0138) and triple negative breast cancer (z score = 1.99, *p* = 0.0471), escalated CTL levels suggested more favorable prognosis when STAT5B had relatively low expression (Supplementary Figures S17F–G). Besides, in acute myeloid leukemia (z score = −2.03, *p* = 0.0421), a higher CTL level indicated a better prognosis when STAT6 had higher expression (Supplementary Figure S17H). Taken together, we concluded that a tumor sample with high STAT1, STAT2, STAT4, and STAT5B expression levels tended to be enriched in T cell dysfunction phenotypes, while STAT6 showed to be the opposite.

What’s more, the results of correlation analyses between STATs expression and tumor immune dysfunction and exclusion-related factors including T dysfunction value in core dataset, normalized z score calling from Cox-PH regression in immunotherapy dataset, normalized z score calling from selection log2FC in CRISPR Screen dataset, and normalized expression value from immune-suppressive cell types were illustrated in Figure 6D. It was shown that STAT1 had significantly positive correlations with normalized z score of many samples calling from selection log2FC in CRISPR Screen dataset. Also, STAT2, STAT4, and STAT5B had several positive correlations with T dysfunction and exclusion values of samples from those datasets. These results again revealed that high STAT1, STAT2, STAT4, and STAT5B expression levels indicated enrichment in T cell dysfunction and exclusion phenotypes in tumor samples.

### Tumor mutation related analysis

**FIGURE 7:**
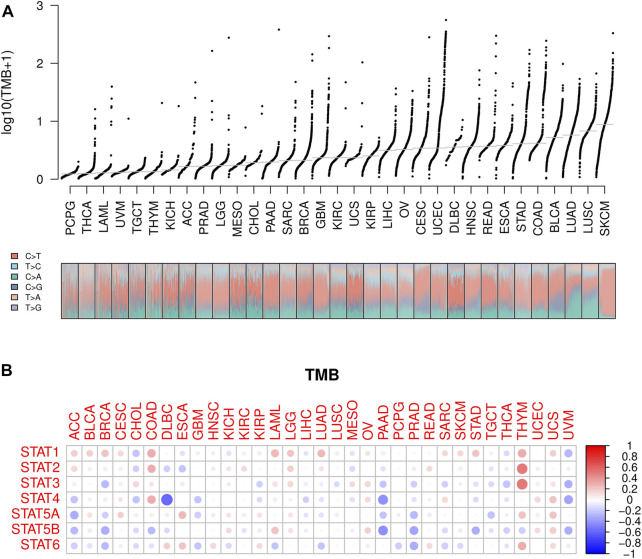
TMB analysis of STATs in pan-cancer. **(A)** TMB values was shown in the form of log10 (TMB+ 1) in 33 types of cancer, in which lower TMB values were identified in PCPG, THCA, and LAML while higher TMB values were revealed in SKCM, LUSC, and LUAD. **(B)** Heatmap demonstrating the correlations between STATs expression and TMB values, from which STAT4 expression was spotted to have a strong negative correlation with TMB value in DLBC, as well as STAT4 and STAT5B expression with TMB values in PAAD. Meanwhile, STAT1, STAT2, STAT3, and STAT6 expression were demonstrated to have positive correlations with TMB values in THYM.

Lower TMB values were seen in PCPG, THCA, and LAML while higher TMB values were shown in SKCM, LUSC, and LUAD (Figure 7A), suggesting potentially favorable effects of immune therapy on SKCM, LUSC, and LUAD instead of PCPG, THCA, and LAML. Subsequently, the association between STATs expression and TMB in pan-cancer was revealed by Spearman correlation analysis in Figure 7B, from which we spotted that STAT4 expression had a strong negative correlation with TMB value in DLBC, as well as STAT4 and STAT5B expression with TMB values in PAAD. Meanwhile, STAT1, STAT2, STAT3, and STAT6 expression were demonstrated to have positive correlations with TMB values in THYM, indicating that high STATs expression might be related to a good immune therapy outcome.

As illustrated in Supplementary Figure S18A, STATs had multiple types but rather low frequencies of genetic alteration in various malignancies. STAT3 had a relatively high alteration frequency of 0.8%, the majority of which were amplification (unknown significance), missense mutation (unknown significance), truncating mutation (unknown significance), missense mutation (putative driver), and inframe mutation (putative driver). In addition, STAT5A, and STAT5B also had relatively high alteration frequencies of 0.6% and 0.9%, mainly amplification (unknown significance), missense mutation (unknown significance), and deep deletion (unknown significance). STATs, in general, were thought to act as tumor driver genes, promoting tumorigenesis. Additionally, TGFBR2, ACVR2A, and SMAD4 were shown to have high alteration frequencies in pan-cancer (Supplementary Figure S17B).

### Drug sensitivity analysis

**FIGURE 8:**
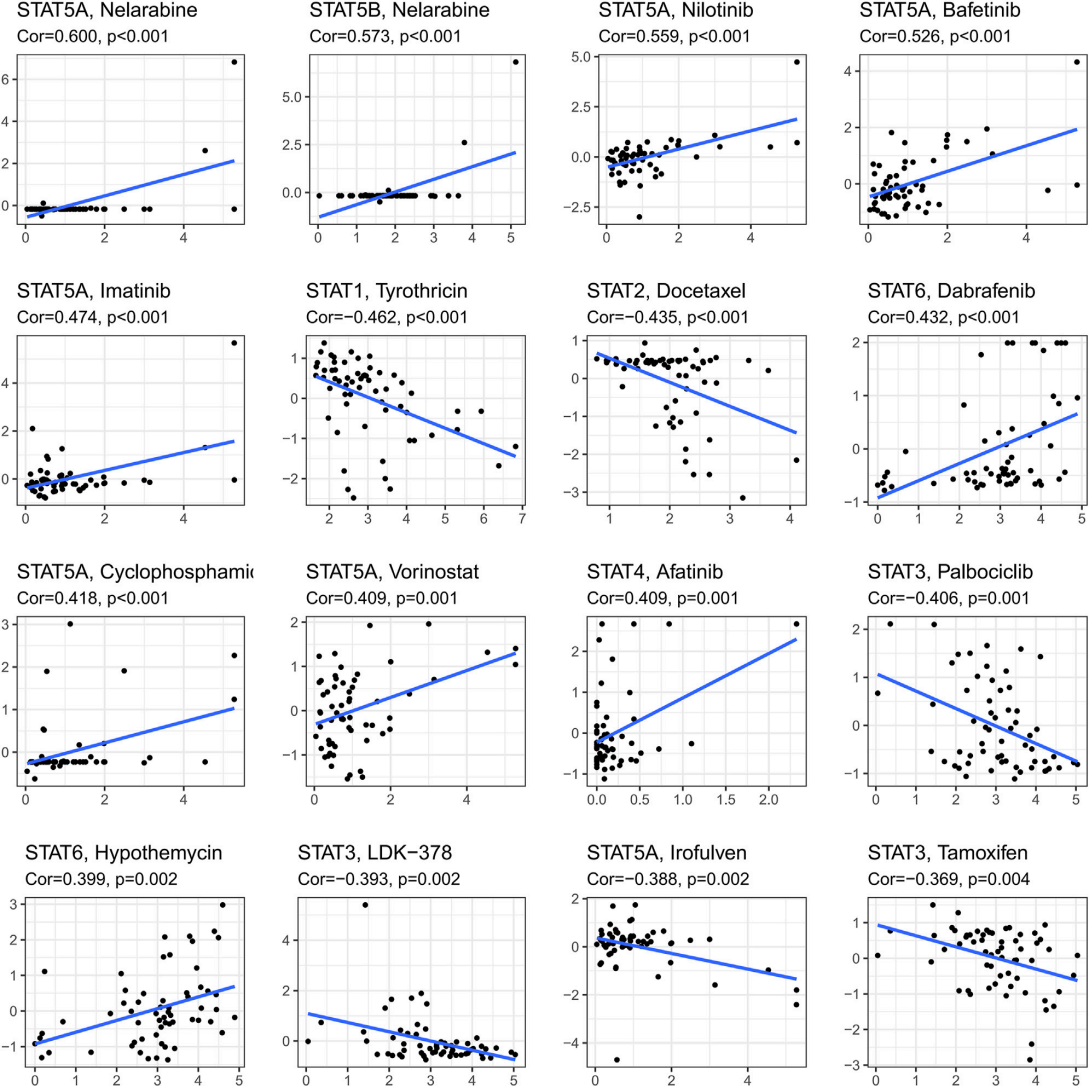
Drug response analysis. The results of correlations between drug sensitivity and STAT1, STAT2, STAT3, STAT4, STAT5A, STAT5B, and STAT6 across TCGA cancers, in which STAT5A expression was shown to have positive correlations with Nelarabine (cor = 0.600, *p* < 0.001), Nilotinib (cor = 0.599, *p* < 0.001), Bafetinib (cor = 0.526, *p* < 0.001), Imatinib (cor = 0.474, *p* < 0.001), Cyclophosphamide (cor = 0.418, *p* < 0.001), and Vorinostat (cor = 0.409, *p* = 0.001), while a negative correlation with Irofulven (cor = −0.338, *p* = 0.002). Moreover, positive correlations were accessed between STAT5B and Nelarabine (cor = 0.573, *p* < 0.001), STAT6 and Dabrafenin (cor = 0.432, *p* < 0.001), and STAT4 and Afatinib (cor = 0.409, *p* = 0.001), while negative correlations were obtained between STAT1 and Tyrothricin (cor = −0.462, *p* < 0.001), and STAT2 and Docetaxel (cor = −0.435, *p* < 0.001). Furthermore, STAT3 was revealed to be negatively linked with Palbociclib (cor = −0.406, *p* = 0.001), LDK-378 (cor = −0.393, *p* = 0.002), and Tamoxifen (cor = −0.369, *p* = 0.004).

Ranked by *p*-value, the association between drug sensitivity and STAT genes was displayed in Figure 8 employing the CellMiner database, which revealed that the expression levels of STAT genes could influence the sensitivity of tumor cells to certain drugs. STAT5A had positive correlations with many types of drugs, including Nelarabine (cor = 0.600, *p* < 0.001), Nilotinib (cor = 0.599, *p* < 0.001), Bafetinib (cor = 0.526, *p* < 0.001), Imatinib (cor = 0.474, *p* < 0.001), Cyclophosphamide (cor = 0.418, *p* < 0.001), and Vorinostat (cor = 0.409, *p* = 0.001), while a negative correlation with Irofulven (cor = −0.338, *p* = 0.002), indicating that patients with high STAT5A expression might be more susceptible to Nelarabine, Nilotinib, Bafetinib, Imatinib, Cyclophosphamide, and Vorinostat, while more resistant to Irofulven. Other positive correlations were accessed between STAT5B and Nelarabine (cor = 0.573, *p* < 0.001), STAT6 and Dabrafenin (cor = 0.432, *p* < 0.001), and STAT4 and Afatinib (cor = 0.409, *p* = 0.001), while negative correlations were obtained between STAT1 and Tyrothricin (cor = −0.462, *p* < 0.001), and STAT2 and Docetaxel (cor = −0.435, *p* < 0.001). What’s more, STAT3 was revealed to antagonize the sensitivity of tumor cells to Palbociclib (cor = −0.406, *p* = 0.001), LDK-378 (cor = −0.393, *p* = 0.002), and Tamoxifen (cor = −0.369, *p* = 0.004).

## Discussion

Generally speaking, as intra-nuclear transcription factors, STATs combine with each other to form a dimer and play a pivotal role in regulating gene expression in the nucleus while activated based on its core SH2 domain. Up to now, up-regulated STAT4 mRNA has been found to be significantly correlated with IFN-γ in patients with gastric cancer and it has been linked to improved disease-free survival (Nishi et al., 2017). Increased STAT5 expression was discovered to be associated with good prognosis in breast cancer (Barash, 2012). STAT1, one of the biomarkers of ovarian cancer, was reported as a disease outcome by Juliana A. J, et al. (Josahkian et al., 2018). In addition, activated STAT6 regulated the signaling pathway to promote the process of lung cancer and might be a therapeutic target for lung cancer (Fu et al., 2019). Above all, there were certain a number of research achievements of STATs in cancers, however, the effects of all members in STAT gene family on 33 types of TCGA tumors have still been unclear so far. Thus, we performed a multidimensional analysis to obtain more results between STAT expression and different tumor types by using the methods of Cox proportional hazards regression, Kruskal-Wallis test, Spearman correlation analysis, ESTIMATE scores, and so on.

According to our multidimensional analysis, it was demonstrated that significantly differential expression of STATs were found among 11,057 samples (10,327 tumor samples and 730 adjacent samples) in 33 TCGA tumor types. WGCNA results revealed that the black module had strong correlations with STAT1 (correlation coefficient = 0.73, *P* = 3e-84), STAT2 (correlation coefficient = 0.63, *P* = 9e-56), STAT4 (correlation coefficient = 0.7, *P* = 2e-75), and STAT5A (correlation coefficient = 0.72, *P* = 2e-80), as well as hallmark allograft rejection (correlation coefficient = 0.78, *P* = 3e-103), interferon γ response (correlation coefficient = 0.78, *P* = 2e-103), inflammatory response (correlation coefficient = 0.6, *P* = 4e-51), interferon *a* response (correlation coefficient = 0.59, *P* = 8e-48), and IL6-JAK-STAT3 signaling (correlation coefficient = 0.47, *P* = 2e-28). That indicated potential relationships between STAT and rejection of allograft, interferon response, inflammatory response and IL6-JAK-STAT3 signaling pathway in pan-cancer, which was consistent with relevant previous research (Darnell et al., 1994; Platanias, 2005; Stepkowski and Kirken, 2006; Mimura et al., 2018; Dambal et al., 2020). Subsequent studies showed the correlation between the expression levels of STATs and a variety of cancer characteristics, such as clinical survival outcomes, immune subtypes, TME, stemness features, and drug sensitivity.

Notably, STAT1 was shown to be highly expressed in most types of tumors as a low-risk gene with HR < 1, which was consistent with the conclusion proposed by Zhang Y, et al. (Zhang and Liu, 2017). Moreover, high expression levels of STAT1 were also revealed to be linked to a better prognosis in patients with ovarian cancer (Josahkian et al., 2018). STAT1 drove gene expression by encoding proteins with IFN signaling, particularly type I and II IFNs, which induced phosphorylation of Janus Kinases (JAKs) and STATs *via* binding their receptors, respectively (Ng et al., 2011). Based on this, IFN-β promoted and sustained the expression of STAT1 ascribed to the Interferon-Stimulate Response Element (ISRE) sites of the STAT1 promoter region, which suggested that the Interferon-Stimulated Gene Factor 3 (ISGF3) played a positive role in auto-regulating STAT1 gene expression (Levy et al., 1988; Darnell et al., 1994; Yuasa and Hijikata, 2016). High STAT1 expression promoted cell cycle suppression and apoptosis, enhanced the tumor suppression effect of p53, stimulated anti-angiogenic factors and inhibited pro-angiogenic factors. Besides, it could accelerate the antigen presentation of dendritic cells by enhancing the cytotoxicity of natural killer (NK) cells and cytotoxic T lymphocyte (CTL) in order to eliminate tumor cells effectively (Adámková et al., 2007; Khodarev et al., 2012). With the above complicated regulations, high expression of STAT1 means better survival outcomes in most tumor types. Nevertheless, there were several cancer types that showed opposite conclusions with STAT1 (Meissl et al., 2017), which implied that the specific circumstance might be attributed to certain tumor types and it could assist in guiding treatment in clinical applications.

It is worth noting that up-regulation of STAT4 was tightly associated with unfavorable overall survival in patients with KIRC and KIRP. Moreover, up-regulated STAT4 and STAT5B expression were discovered to be linked with better prognosis in patients with PAAD. For BLCA and SARC, high STAT6 expression also indicated increased survival rates. The results mentioned above that haven’t been researched before might lead to a new way cancer detection and prediction.

The investigators have found a correlation between expressional STAT3/STAT5 ratio and prognosis in colon carcinoma (Klupp et al., 2015). In particular, STAT3 was under-expressed while STAT4 and STAT5 were over-expressed in colon cancer tissue. Furthermore, increased expression of STAT1 and STAT3 in tumor tissue implicated adverse prognosis whereas higher STAT4 or STAT5 expression meant improved survival. In this study, however, STAT4 was shown to be lowly expressed in COAD during the whole pathological process across stages I to IV with a declining trend.

Six immune subtypes have been identified by Thorsson, V, et al. (Thorsson et al., 2018), but the relationship with 33 TCGA types of tumor was unclear until now. Based on this, we performed a Kruskal-Wallis test to obtain the immune features of STATs and compared the differential expression of STATs across C1-C6. Significant variation was shown in lung cancer tissue, SARC, and SKCM. Immune subtypes were identified to possess a conclusive correlation with tumor microenvironment, which suggested the prognosis and therapeutic options (Soldevilla et al., 2019). Made up of tumor cells themselves, surrounding stromal cells, immune cells, and micro-vessels, TME plays an important role in tumorigenesis with three features of hypoxia, chronic inflammation, and immunosuppression. Since stromal cells within the TME were genetically stable, they could be used as a therapeutic response target to reduce drug resistance and tumor recurrence risk (Quail and Joyce, 2013). Thus, we used stromal score to access the proportion of stromal cells in TME and reflect the tumor purity. The high stromal score showed that the expression of STATs, especially STAT4, were positively correlated with the number of stromal cells. Additionally, STAT1, STAT3, STAT5A and STAT6 were highly expressed in LGG with high stromal scores and thus low purity, a result of which was more likely to make a definite diagnosis of the malignant entity and have a direct association with reducing survival time. The clinical significance was consistent with investigators Chuanbao Zhang, et al., in 2017 (Zhang et al., 2017). Moreover, the results of immune infiltration analysis showed preference of STATs’ distribution in certain immune cells, which might be associated with unique function of STATs in immune response. And that STATs expression were positively correlated with immune infiltration were consistent with the results of immune scores.

CSCs, with the features of self-renewing, differentiating, and proliferating, were capable of reconstructing and propagating tumors. The potential characteristics of CSCs depended on signaling pathways, TME, drug resistance markers, cell surface molecules, and so on, which provided an unprecedented treatment strategy for overcoming tumor recurrence and chemo-resistance by targeting CSCs (Prasad et al., 2020). In order to further access the correlation between stemness features and STATs, the OCLR algorithm was performed to calculate mDNAss and mRNAss to demonstrate the stemness properties. Many regulatory molecules affecting the stemness of breast cancer may serve as therapeutic targets in clinical applications including STATs (Lu et al., 2018; Misra et al., 2018; Gao et al., 2019; Yu et al., 2019). Thus, expression levels of STATs might act as an important part in maintaining cancer cell stemness properties.

In-depth immunotherapy research was carried out utilizing the TIDE database in order to better understand the impact of STATs on tumor immunotherapy. Higher TIDE prediction scores are linked with worse immunotherapy response and unfavorable survival outcomes under anti-programmed cell death 1 receptor (PD1) and anti-cytotoxic T lymphocyte-associated protein 4 (CTLA4) therapies in pan-cancer (Jiang et al., 2018). Our study revealed that high STAT1, STAT2, STAT4, and STAT5B expression levels indicated enrichment in T cell dysfunction and exclusion phenotypes, which suggested worse immune checkpoint blocker responses and worse patient survival. Intriguingly, it was reported that altered transcriptional output in JAK-STAT signaling pathway might be involved with KIRC patients’ responsiveness to immune checkpoint therapy (Miao et al., 2018). Moreover, transcription alteration of STATs were also revealed to be associated with altered antitumor T cell responses (Freitas et al., 2022).

A notable association was found between STATs and drug response. For instance, STAT5 had positive correlations with various types of drugs, while STAT1 and STAT2 were negatively correlated with Tyrothricin and Docetaxel, respectively. Different expression levels of STATs indicated rather increased sensitivity or resistance to anti-tumor drugs.

The research on the roles of STATs in pan-cancer was not processed before *via* a multidimensional analysis, but there were still some limitations in our present study. Genes and habitus were so diverse among Americans, Asians and European that samples of the database might be not suitable for Asians. Otherwise, the results were just testified by one single database, thus further verification and validation should be processed by other public databases to increase credibility. Last but not least, due to the inherent defects of bioinformatics analysis, we intend to test and verify the potential mechanism with molecular and animal experiments in the near future. Although investigators has shown some relevance between STATs expression and targeted therapy, more complex mechanisms are still void, hopefully our study provides a new clue to the application of anti-cancer drugs.

## Conclusion

STATs were revealed to have extensive and profound associations with tumors by regulating gene expression in the nucleus, which occupied a significant status in pan-cancer. We carried out deep research to multi-dimensionally analyze the roles of the STAT gene family in differential and co-expression analysis, WGCNA, clinical features, immune subtypes, tumor stemness, tumor purity, immune infiltration, immunotherapy response, tumor mutation and drug sensitivity across 33 TCGA types of tumor. From that we revealed STATs to be biomarkers for prognostic prediction and therapeutic guidance in pan-cancer. Hopefully our findings could provide a valuable reference for scientific research and clinical applications on STATs in the future.

## Abbreviation

STAT, Signal transducer and activator of transcription; TCGA, The Cancer Genome Atlas; SH2, Src-homology; Arg, Arginine; Tyr, Tyrosine; IFN, Interferon; OCLR, One-class logistic regression; ESTIMATE, Estimation of stromal and immune cells in malignant tumors using expression data; FPKM, Fragments Per Kilobase per Million; KM, Kaplan-Meier; OS, Overall survival; DFI, Disease-free interval; PFI, Progression-free interval; DSS, Disease-specific survival; HR, Hazard Ratios; CSCs, Cancer Stem cells; mDNAss, DNA methylation-based stemness index; mRNAss, mRNA expression-based stemness index; TME, The tumor microenvironment; TMB, Tumor mutation burden; PPI, Protein-protein interaction; JAKs, Janus Kinases; ISRE, Interferon-Stimulate Response Element; ISGF3, Interferon-Stimulated Gene Factor 3; NK, Natural Killer; CTL, Cytotoxic T Lymphocyte.

## Data Availability

Publicly available datasets were analyzed in this study. This data can be found here: The datasets generated and/or analysed during the current study are available in the TCGA program (https://portal.gdc.cancer.gov).

## Abbreviations

STAT, Signal transducer and activator of transcription; TCGA, The Cancer Genome Atlas; SH2, Src-homology; Arg, Arginine; Tyr, Tyrosine; IFN, Interferon; OCLR, One-class logistic regression; ESTIMATE, Estimation of stromal and immune cells in malignant tumors using expression data; FPKM, Fragments Per Kilobase per Million; KM, Kaplan-Meier; OS, Overall survival; DFI, Disease-free interval; PFI, Progression-free interval; DSS, Disease-specific survival; HR, Hazard Ratios; CSCs, Cancer Stem cells; mDNAss, DNA methylation-based stemness index; mRNAss, mRNA expression-based stemness index; TME, The tumor microenvironment; TMB, Tumor mutation burden; PPI, Protein-protein interaction; JAKs, Janus Kinases; ISRE, Interferon-Stimulate Response Element; ISGF3, Interferon-Stimulated Gene Factor 3; NK, Natural Killer; CTL, Cytotoxic T Lymphocyte.
