# Supplementary material for: Pan-cancer analysis reveals signal transducer and activator of transcription (STAT) gene family as biomarkers for prognostic prediction and therapeutic guidance
Source: Front Genet. 2023 Mar 9;14:1120500. doi: 10.3389/fgene.2023.1120500 (PMC10034013; doi:10.3389/fgene.2023.1120500)
Supplement: Supplementary file 1 [file DataSheet2.pdf]

Supplementary material

Table S1 33 TCGA cancer types and their abbreviations.

Cancer	Abbreviation	Cancer	Abbreviation
Adrenocortical carcinoma	ACC	Lung adenocarcinoma	LUAD
Bladder Urothelial Carcinoma	BLCA	Lung squamous cell carcinoma	LUSC
Breast invasive carcinoma	BRCA	Mesothelioma	MESO
Cervical squamous cell carcinoma and endocervical adenocarcinoma	CESC	Ovarian serous cystadenocarcinoma	OV
Cholangiocarcinoma	CHOL	Pancreatic adenocarcinoma	PAAD
Colon adenocarcinoma	COAD	Pheochromocytoma and Paraganglioma	PCPG
Lymphoid Neoplasm Diffuse Large B-cell Lymphoma	DLBC	Prostate adenocarcinoma	PRAD
Esophageal carcinoma	ESCA	Rectum adenocarcinoma	READ
Glioblastoma multiforme	GBM	Sarcoma	SARC
Head and Neck squamous cell carcinoma	HNSC	Skin Cutaneous Melanoma	SKCM
Kidney Chromophobe	KICH	Stomach adenocarcinoma	STAD
Kidney renal clear cell carcinoma	KIRC	Testicular Germ Cell Tumors	TGCT
Kidney renal papillary cell carcinoma	KIRP	Thyroid carcinoma	THCA
Acute Myeloid Leukemia	LAML	Thymoma	THYM
Brain Lower Grade Glioma	LGG	Uterine Corpus Endometrial Carcinoma	UCEC
Liver hepatocellular carcinoma	LIHC	Uterine Carcinosarcoma	UCS
Uveal Melanoma	UVM		

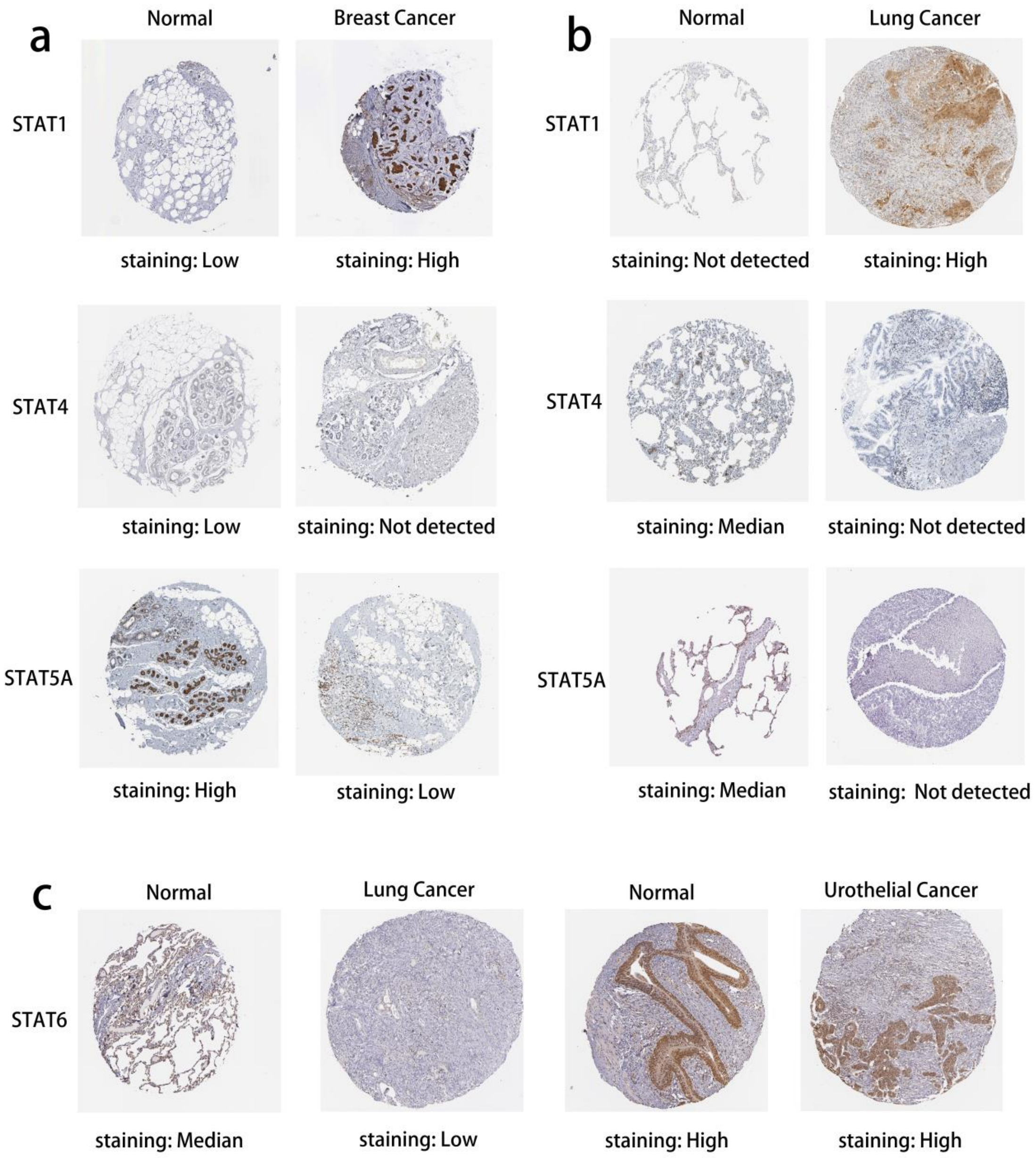

Figure S1 The Human Protein Atlas validation.

(a) The protein expression level of STAT1 was higher in breast cancer than normal tissues, while STAT4 and STAT5A were higher in normal tissues. (b) The protein expression level of STAT1 was higher in lung cancer than normal tissues, while STAT4 and STAT5A were the opposite. (c) The protein expression level of STAT6 was higher in lung cancer than normal tissues, while both high in normal and urothelial cancer tissues.

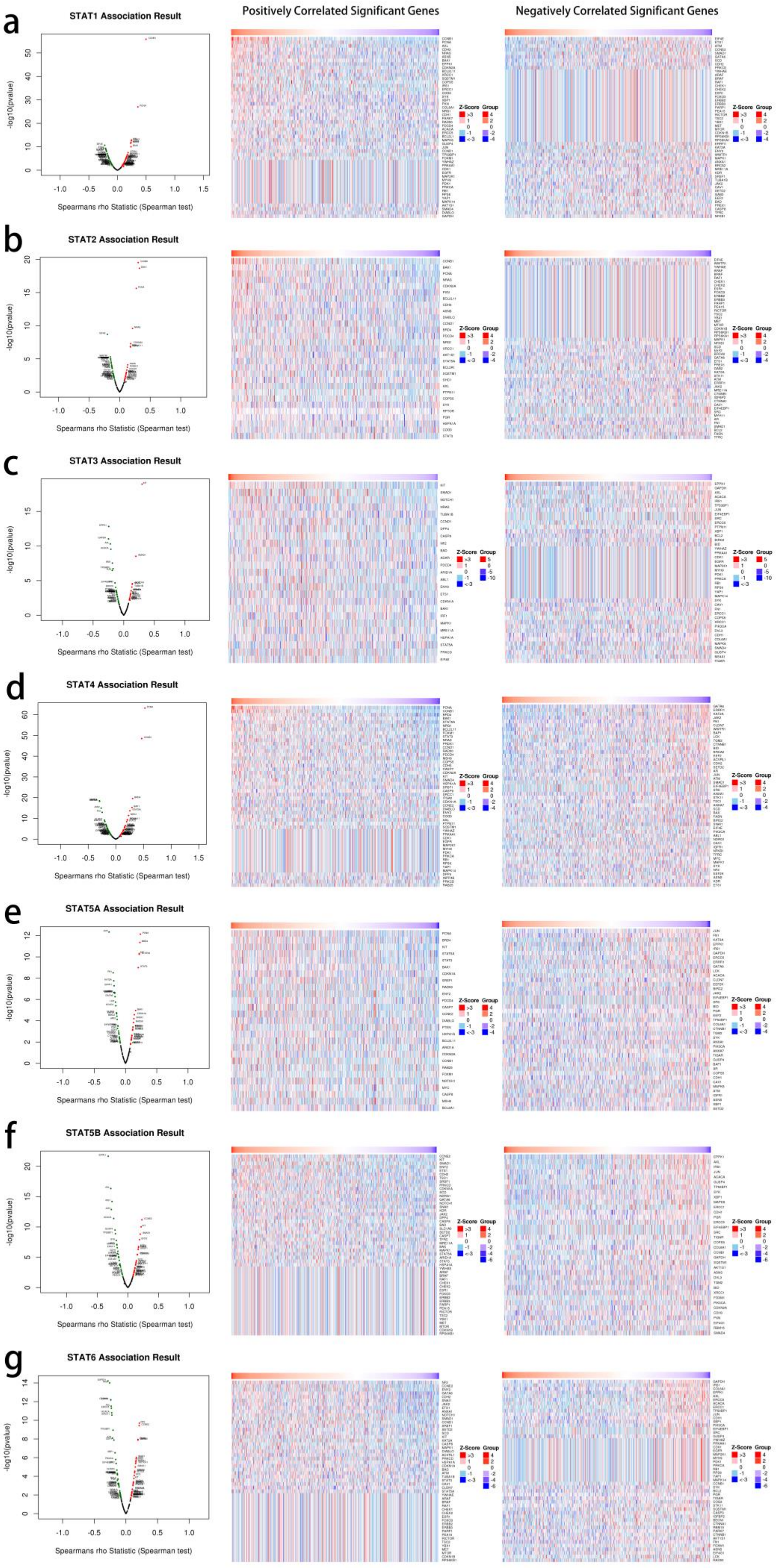
a
STAT1 Association Result
Positively Correlated Significant Genes
Negatively Correlated Significant Genes
-log10(pvalue)
Spearmans rho Statistic (Spearman test)
Z-Score
Group
b
STAT2 Association Result
c
STAT3 Association Result
d
STAT4 Association Result
e
STAT5A Association Result
f
STAT5B Association Result
g
STAT6 Association Result

Figure S2 The LinkedOmics database validation.

Volcano plots and heatmaps showing the associated protein genes' expression levels of STAT1 (a), STAT2 (b), STAT3 (c), STAT4 (d), STAT5A (d), STAT5B (e), and STAT6 (f), including positively and negatively correlated significant genes.

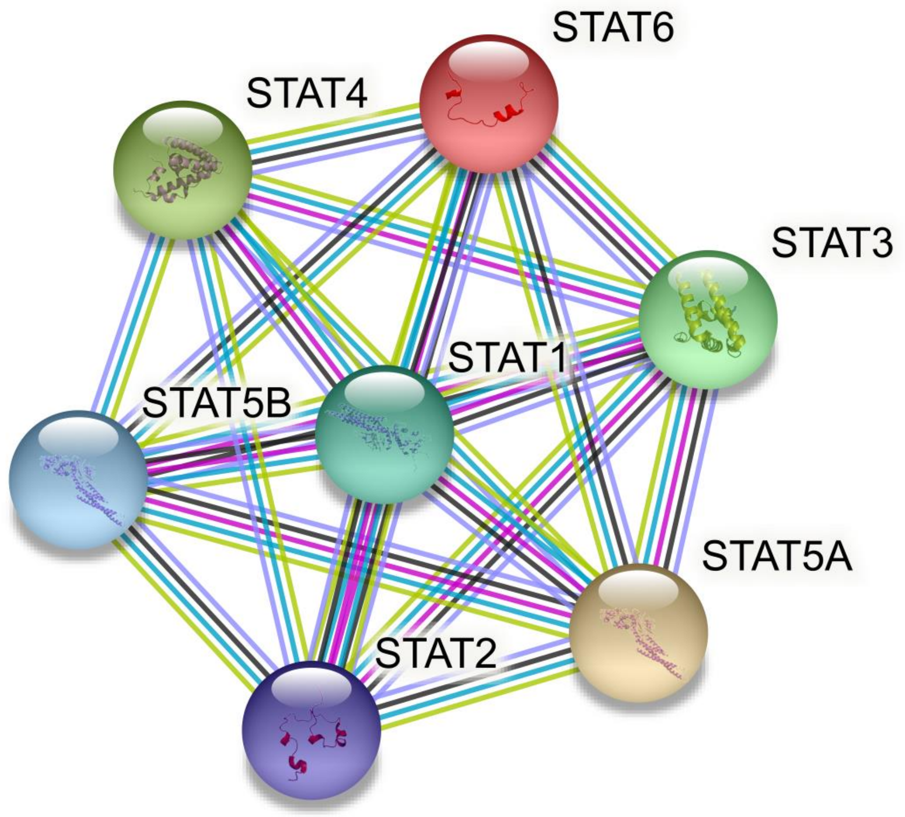

Figure S3 The STRING database validation.

The PPI network of STAT family members revealed a remarkably tight connections between each two STATs.

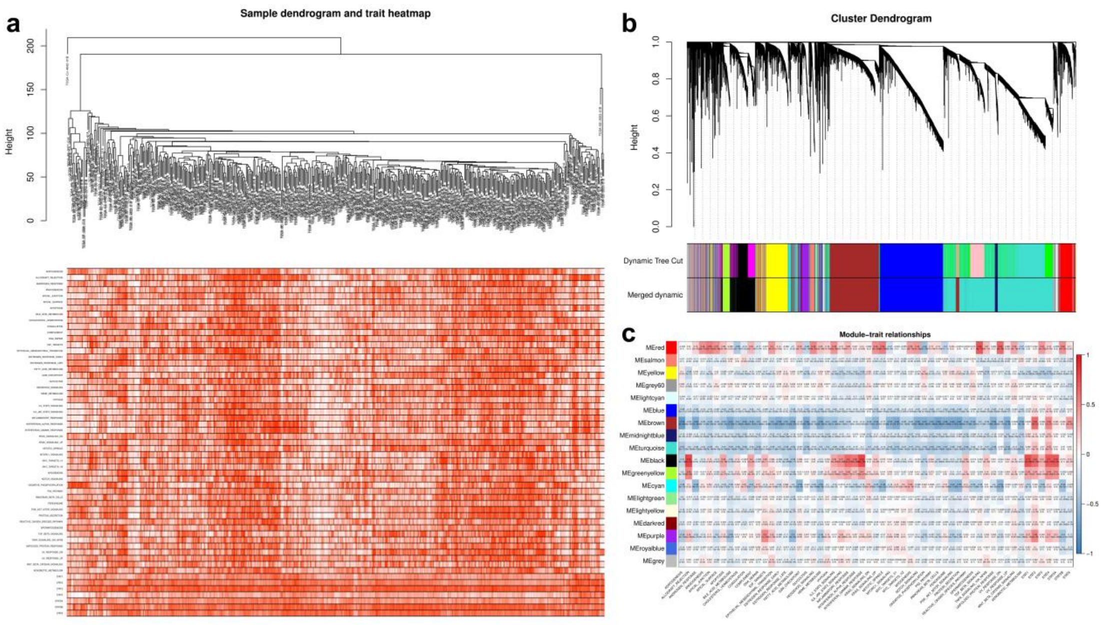

Figure S4 WGCNA of STATs in KIRC.

(a) Sample dendrogram and trait heatmap showing the expression of the 50 hallmark gene sets and STATs. (b) Cluster dendrogram of DEGs between tumor and para-tumor KIRC tissues revealing the co-expression modules with different branches and color blocks. (c) Module trait relationships heatmap demonstrating that the black module had generally strong correlations with STATs, especially STAT1 (correlation coefficient = 0.73, *P* = 3e-84), STAT2 (correlation coefficient = 0.63, *P* = 9e-56), STAT4 (correlation coefficient = 0.7, *P* = 2e-75), and STAT5A (correlation coefficient = 0.72, *P* = 2e-80), as well as hallmark allograft rejection (correlation coefficient = 0.78, *P* = 3e-103), interferon γ response (correlation coefficient = 0.78, *P* = 2e-103), inflammatory response (correlation coefficient = 0.6, *P* = 4e-51), interferon α response (correlation coefficient = 0.59, *P* = 8e-48), and IL6-JAK-STAT3 signaling (correlation coefficient = 0.47, *P* = 2e-28).

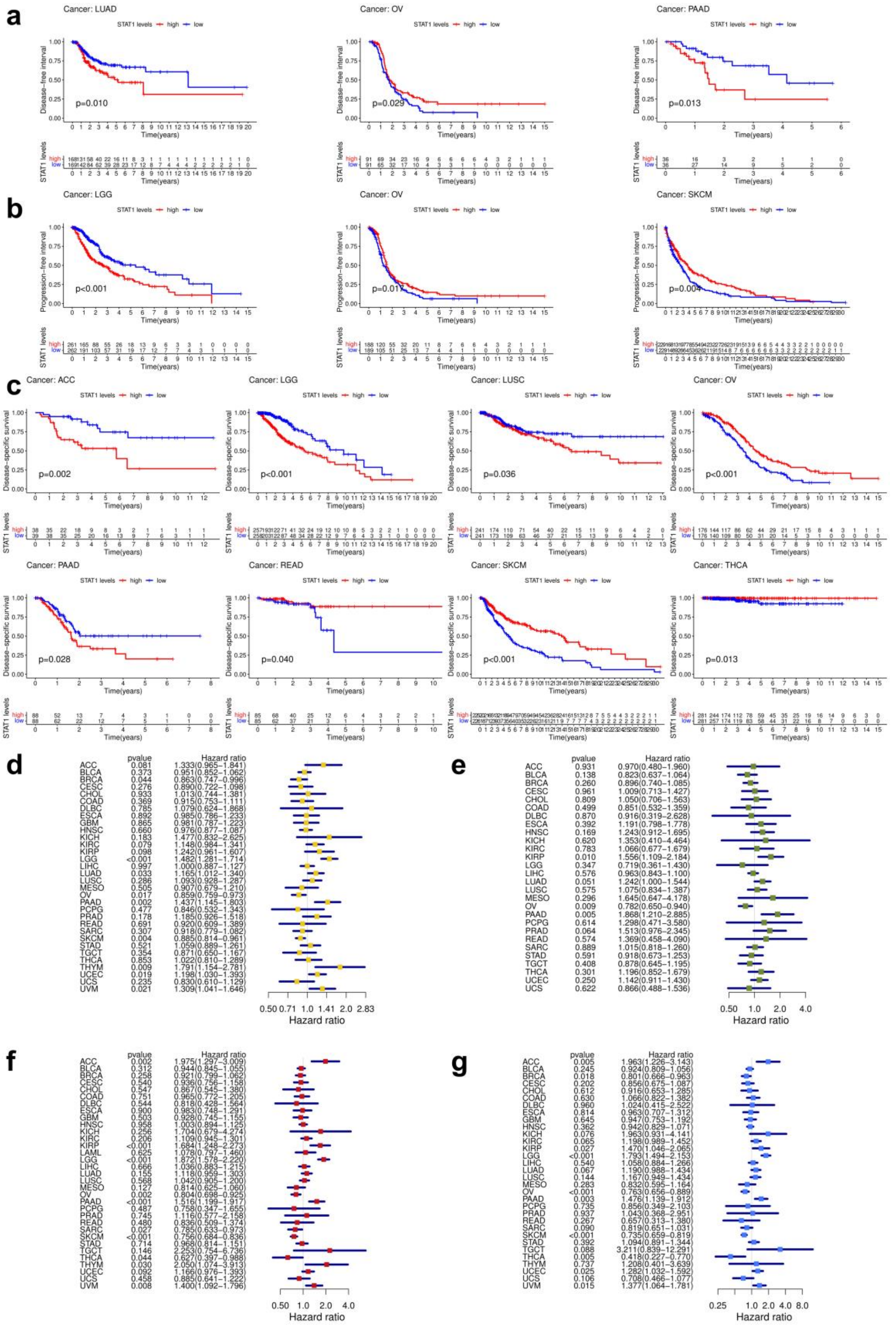

Figure S5 Supplementary survival analysis of STAT1 in pan-cancer.

(a) Statistically significant differences were found between high and low expression of STAT1 in DSI of LUAD (*P* = 0.010), OV (*P* = 0.029), and PAAD (*P* = 0.013) patients. (b) Statistically significant differences were obtained between high and low expression of STAT1 in DFI of LGG

(*P* < 0.001), OV (*P* = 0.017), and SKCM (*P* = 0.004) patients. (c) Statistically significant differences were reached between high and low expression of STAT1 in DSS of ACC (*P* = 0.002), LGG (*P* < 0.001), LUSC (*P* = 0.036), OV (*P* < 0.001), PAAD (*P* = 0.028), READ (*P* = 0.040), SKCM (*P* < 0.001), and THCA (*P* = 0.013) patients. (d) Forest plot depicted univariate Cox hazards regression analysis results measured by OS that STAT1 was a protective factor in BRCA (*P* = 0.044, HR = 0.863), OV (*P* = 0.017, HR = 0.859), and SKCM (*P* = 0.004, HR = 0.885), while a risk factor in LGG (*P* < 0.001, HR = 1.482), LUAD (*P* = 0.033, HR = 1.165), PAAD (*P* = 0.002, HR = 1.437), THYM (*P* = 0.009, HR = 1.791), UCEC (*P* = 0.019, HR = 1.198), and UVM (*P* = 0.021, HR = 1.309). (e) Forest plot demonstrated univariate Cox hazards regression analysis results measured by DFI that STAT1 was a protective factor in OV (*P* = 0.009, HR = 0.782), while a risk factor in KIRP (*P* = 0.010, HR = 1.556) and PAAD (*P* = 0.005, HR = 1.868). (f) Forest plot revealed univariate Cox hazards regression analysis results measured by PFI that STAT1 was a protective factor in OV (*P* = 0.002, HR = 0.804), SARC (*P* = 0.027, HR = 0.785) SKCM (*P* < 0.001, HR = 0.756), and THCA (*P* = 0.044, HR = 0.627), while a risk factor in ACC (*P* = 0.002, HR = 1.975), KIRP (*P* < 0.001, HR = 1.684), LGG (*P* < 0.001, HR = 1.872), PAAD (*P* < 0.001, HR = 1.516), THYM (*P* = 0.030, HR = 2.050), and UVM (*P* = 0.008, HR = 1.400). (g) Forest plot showed univariate Cox hazards regression analysis results measured by DSS that STAT1 was a protective factor in BRCA (*P* = 0.018, HR = 0.801), OV (*P* < 0.001, HR = 0.763), SKCM (*P* < 0.001, HR = 0.735), THCA (*P* = 0.005, HR = 0.418) while a risk factor in ACC (*P* = 0.005, HR = 1.963), KIRP (*P* = 0.027, HR = 1.470), LGG (*P* < 0.001, HR = 1.793), PAAD (*P* = 0.003, HR = 1.476), UCEC (*P* = 0.025, HR = 1.282), and UVM (*P* = 0.015, HR = 1.377).

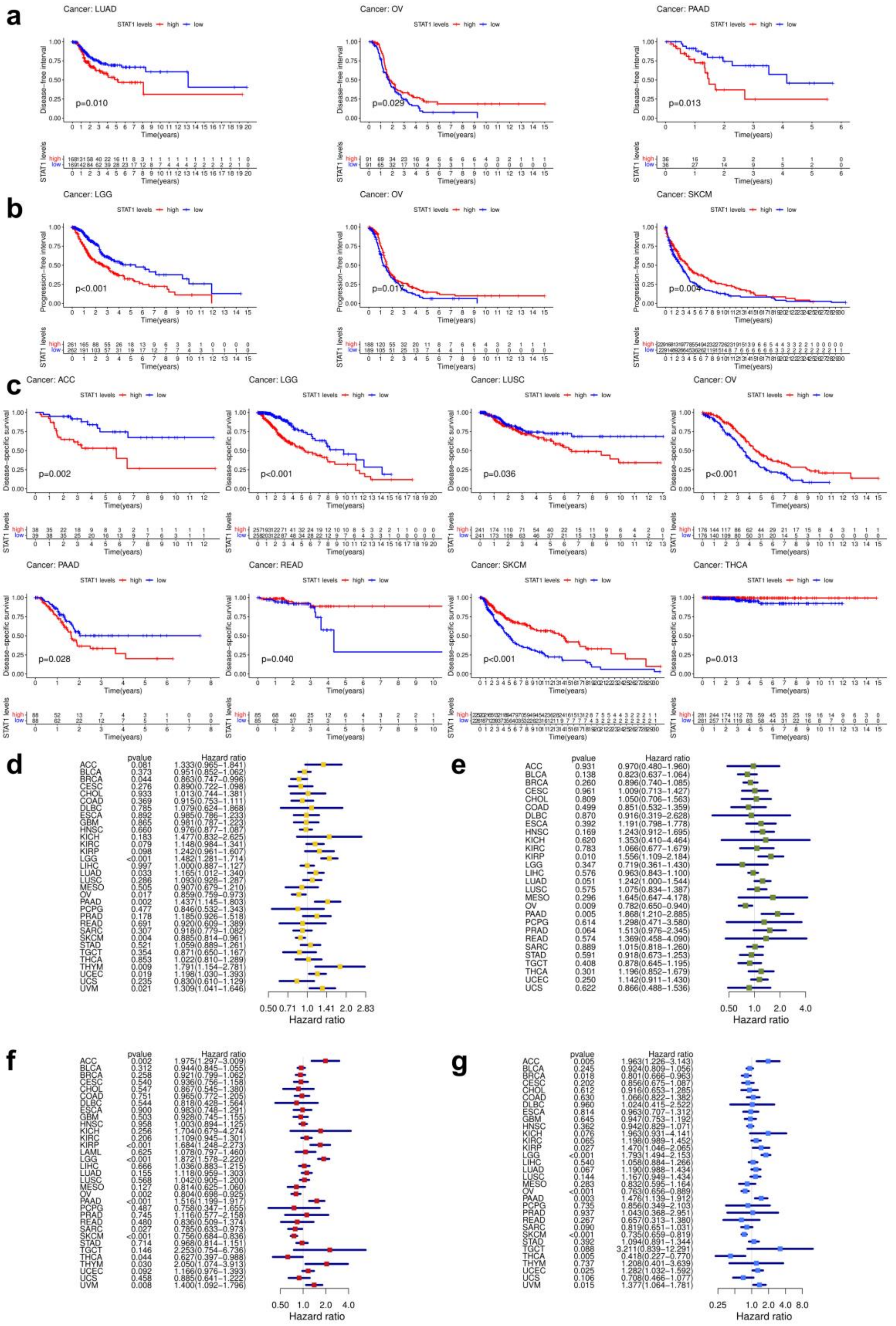

Figure S6 Supplementary survival analysis of STAT3 in pan-cancer.

(a) Statistically significant differences were found between high and low expression of STAT3 in DSI of BRCA (P = 0.043), LUSC (P = 0.003), and PCPG (P = 0.031) patients. (b) Statistically significant differences were obtained between high and low expression of STAT3 in DFI of

COAD (*P* = 0.015), HNSC (*P* = 0.046), LGG (*P* = 0.002), PRAD (*P* = 0.024), and SKCM (*P* = 0.003) patients. (c) Statistically significant differences were reached between high and low expression of STAT3 in DSS of COAD (*P* = 0.045), KICH (*P* < 0.001), LGG (*P* < 0.001), and SKCM (*P* < 0.001) patients. (d) Forest plot depicted univariate Cox hazards regression analysis results measured by OS that STAT3 was a protective factor in SARC (*P* = 0.005, HR = 0.600) and SKCM (*P* < 0.001, HR = 0.659), while a risk factor in KIRP (*P* = 0.032, HR = 1.987), LGG (*P* < 0.001, HR = 2.424), and UVM (*P* = 0.010, HR = 2.406). (e) Forest plot demonstrated univariate Cox hazards regression analysis results measured by DFI that STAT3 was a protective factor in SARC (*P* = 0.013, HR = 0.590), while a risk factor in LUSC (*P* = 0.004, HR = 1.991). (f) Forest plot revealed univariate Cox hazards regression analysis results measured by PFI that STAT3 was a protective factor in HNSC (*P* = 0.009, HR = 0.742), SARC (*P* = 0.010, HR = 0.676), and SKCM (*P* = 0.001, HR = 0.741), while a risk factor in LGG (*P* < 0.001, HR = 2.230), and UVM (*P* = 0.004, HR = 2.377). (g) Forest plot showed univariate Cox hazards regression analysis results measured by DSS that STAT3 was a protective factor in HNSC (*P* = 0.045, HR = 0.760), SARC (*P* = 0.007, HR = 0.590), and SKCM (*P* < 0.001, HR = 0.633) while a risk factor in LGG (*P* < 0.001, HR = 2.945), PAAD (*P* = 0.003, HR = 1.476) and UVM (*P* = 0.014, HR = 2.401).

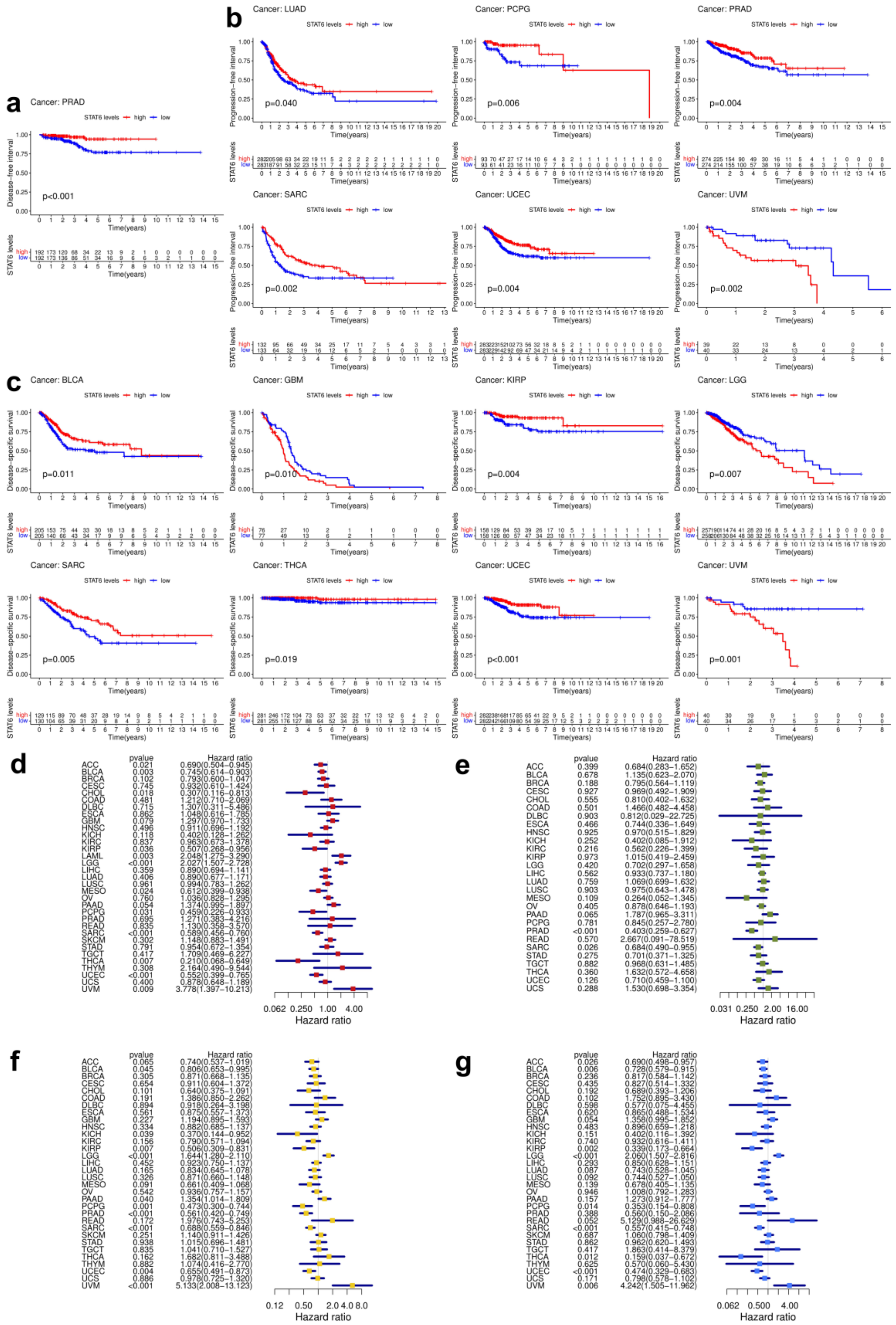

Figure S7 Supplementary survival analysis of STAT6 in pan-cancer.

(a) Statistically significant differences were found between high and low expression of STAT6 in DSI of PRAD (P < 0.001) patients. (b) Statistically significant differences were obtained between high and low expression of STAT6 in DFI of LUAD (P = 0.040), PCPG (P = 0.006), PRAD (P =

0.004), SARC (*P* = 0.002), UCEC (*P* = 0.004), and UVM (*P* = 0.002) patients. (c) Statistically significant differences were reached between high and low expression of STAT6 in DSS of BLCA (*P* = 0.011), GBM (*P* = 0.010), KIRP (*P* = 0.004), and LGG (*P* = 0.007), SARC (*P* = 0.005), THCA (*P* = 0.019), UCEC (*P* < 0.001), and UVM (*P* = 0.001) patients. (d) Forest plot depicted univariate Cox hazards regression analysis results measured by OS that STAT6 was a protective factor in ACC (*P* = 0.021, HR = 0.690), BLCA (*P* = 0.003, HR = 0.745), CHOL (*P* = 0.018, HR = 0.307), KIRP (*P* = 0.036, HR = 0.507), MESO (*P* = 0.024, HR = 0.612), PCPG (*P* = 0.031, HR = 0.459), SARC (*P* < 0.001, HR = 0.589), THCA (*P* = 0.007, HR = 0.210), and UCEC (*P* < 0.001, HR = 0.552), while a risk factor in LAML (*P* = 0.003, HR = 2.048), LGG (*P* < 0.001, HR = 2.027), and UVM (*P* = 0.009, HR = 3.778). (e) Forest plot demonstrated univariate Cox hazards regression analysis results measured by DFI that STAT6 was a protective factor in PRAD (*P* < 0.001, HR = 0.403) and SARC (*P* = 0.026, HR = 0.684). (f) Forest plot revealed univariate Cox hazards regression analysis results measured by PFI that STAT6 was a protective factor in BLCA (*P* = 0.045, HR = 0.806), KICH (*P* = 0.039, HR = 0.370), KIRP (*P* = 0.007, HR = 0.506), PCPG (*P* = 0.001, HR = 0.473), PRAD (*P* < 0.001, HR = 0.561), SARC (*P* < 0.001, HR = 0.688), and UCEC (*P* = 0.004, HR = 0.655), while a risk factor in LGG (*P* < 0.001, HR = 1.644), PAAD (*P* = 0.040, HR = 1.354), and UVM (*P* < 0.001, HR = 5.133). (g) Forest plot showed univariate Cox hazards regression analysis results measured by DSS that STAT6 was a protective factor in ACC (*P* = 0.026, HR = 0.690), BLCA (*P* = 0.006, HR = 0.728), KIRP (*P* = 0.002, HR = 0.339), PCPG (*P* = 0.014, HR = 0.353), SARC (*P* < 0.001, HR = 0.557), THCA (*P* = 0.012, HR = 0.159), and UCEC (*P* < 0.001, HR = 0.474), while a risk factor in LGG (*P* < 0.001, HR = 2.060) and UVM (*P* = 0.006, HR = 4.242).

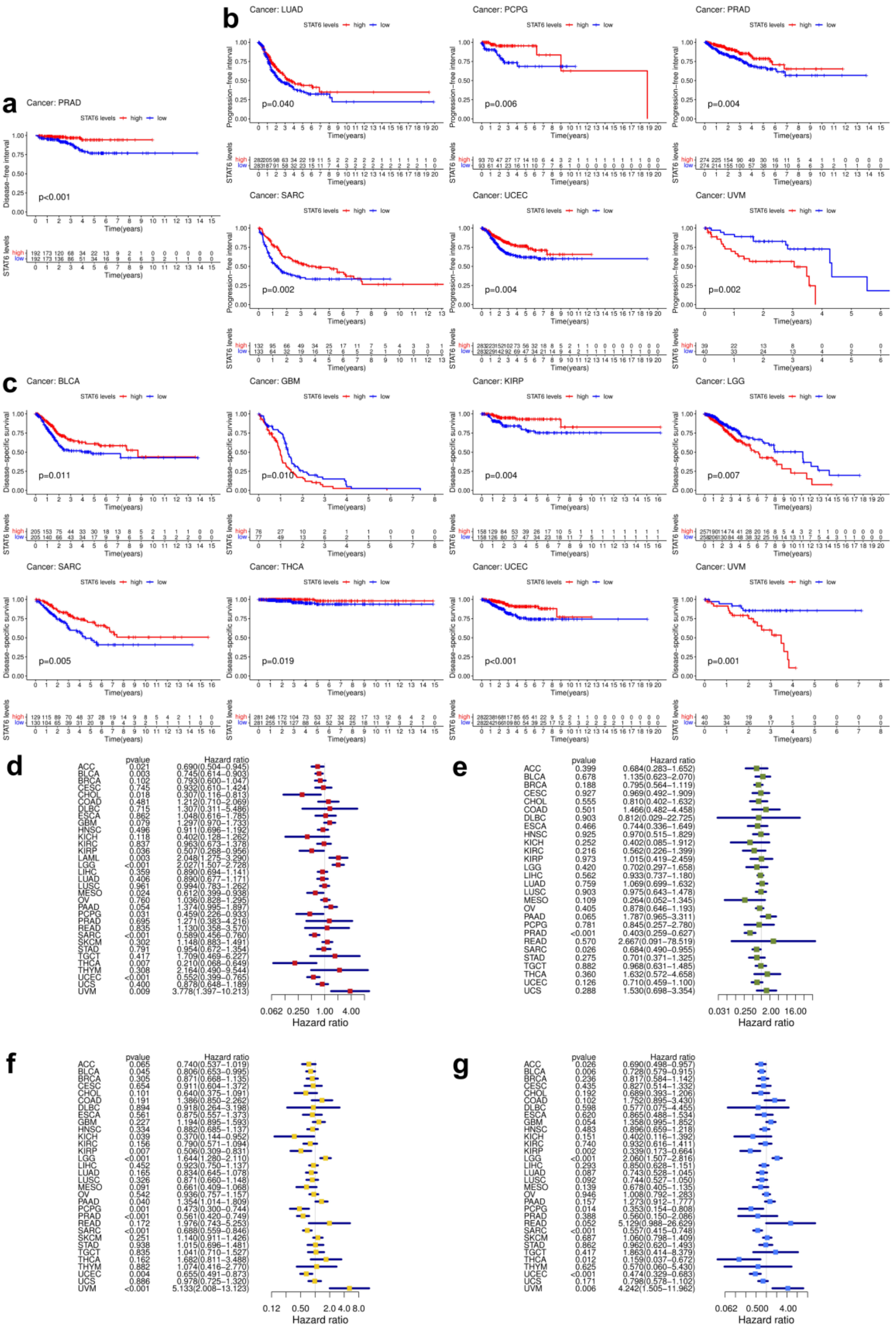

Figure S8 Multivariate Cox hazards regression of STATs in KIRC.

(a) The risk curves displaying KIRC patients of high- and low-risk groups in increasing risk score in all, train, and test sets. (b) The risk scatter plots demonstrating sensor (alive or dead) and survival time of patients with ascending risk score in in all, train, and test sets. (c) K-M survival plots all showing statistically significant differences (p < 0.001) between high- and low-risk KIRC patients in in all, train, and test sets. (d) ROC curves revealing a favorable reliability of the prognostic model in all set (AUC = 0.745), train set (AUC = 0.752) and test set (AUC = 0.757). (e) The univariate Cox hazards regression indicating that risk score had a HR = 14.783 (95% CI (5.652-38.664), *P* < 0.001). (f) The multivariate Cox hazards regression suggesting that risk score had a HR = 7.204 (95% CI (2.293-22.631), *P* < 0.001).

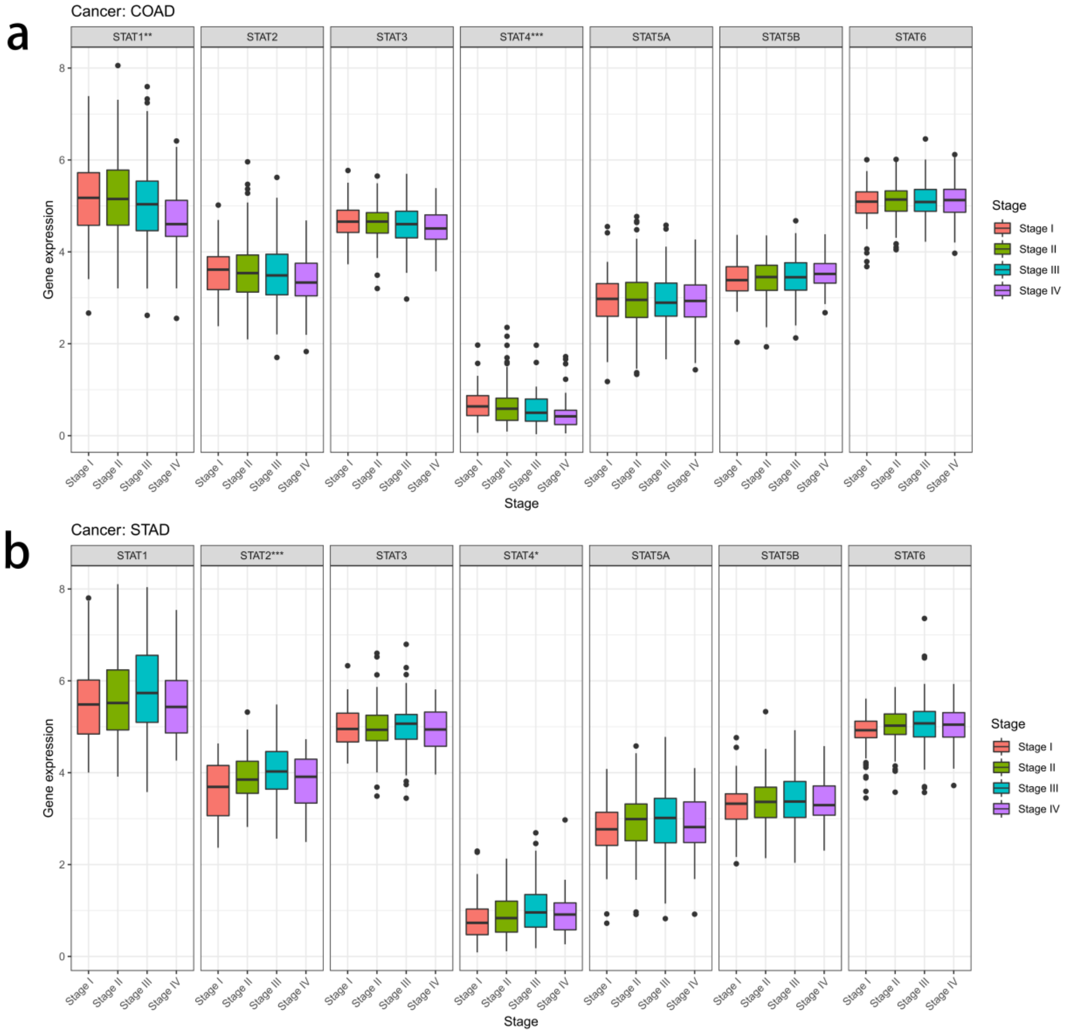

Figure S9 Tumor stages analysis of STATs in pan-cancer.

(a) Box plots displaying the differential expression of STATs in COAD among stage I, II, III, and IV of COAD, in which STAT1 ($P < 0.01$) and STAT4 ($P < 0.001$) showed statistical significance.

(b) Box plots illustrating differential expression of STATs in STAD among stage I, II, III, and IV, in which the expression of STAT2 ($P < 0.001$) and STAT4 ($P < 0.05$) exhibited statistical significance.

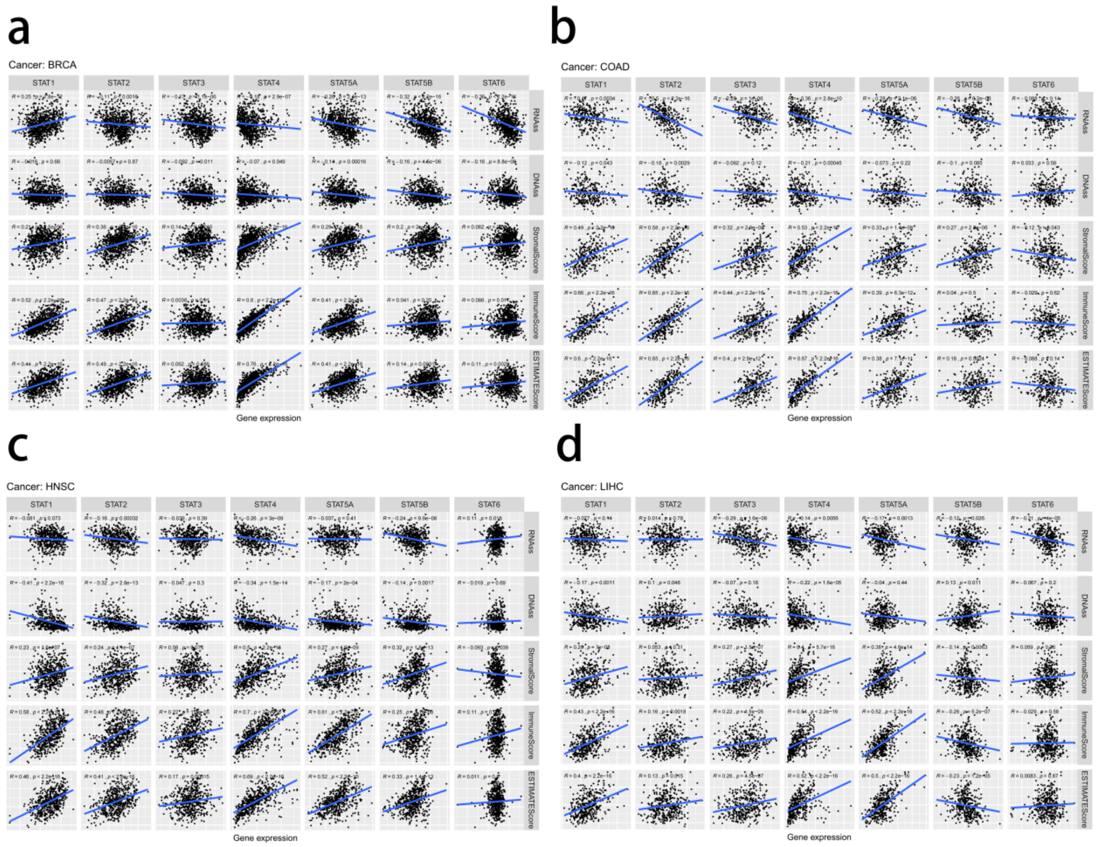

Figure S10 Tumor stemness and tumor purity analyses of STATs in pan-cancer.

(a) The correlation between STATs and mRNAss, mDNAss, stromal scores, immune scores and ESTIMTE scores in BRCA. (b) The correlation between STATs and mRNAss, mDNAss, stromal scores, immune scores and ESTIMTE scores in COAD. (c) The correlation between STATs and mRNAss, mDNAss, stromal scores, immune scores and ESTIMTE scores in HNSC. (d) The correlation between STATs and mRNAss, mDNAss, stromal scores, immune scores and ESTIMTE scores in LIHC.

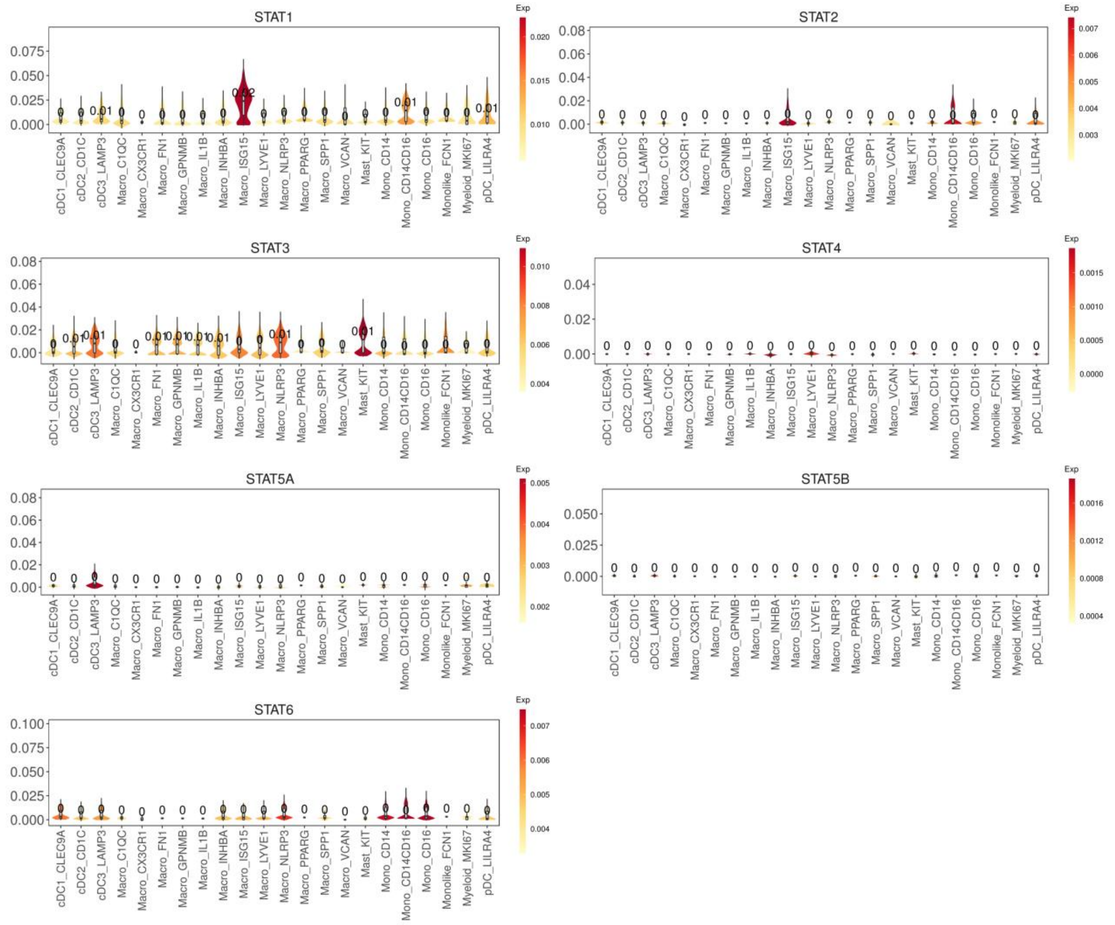

Figure S11 Supplementary figures for Figure 6a.

Violin plot showing the distribution of STAT1 (a), STAT2 (b), STAT3 (c), STAT4 (d), STAT5A (e), STAT5B (f), and STAT6 (g) expression in different types of tumor infiltrating myeloid cells in pan-cancer, which showed that STAT1 was remarkably high expressed in macrophages with marker ISG15, while STAT3 expression was significantly escalated in mast cells with marker KIT.

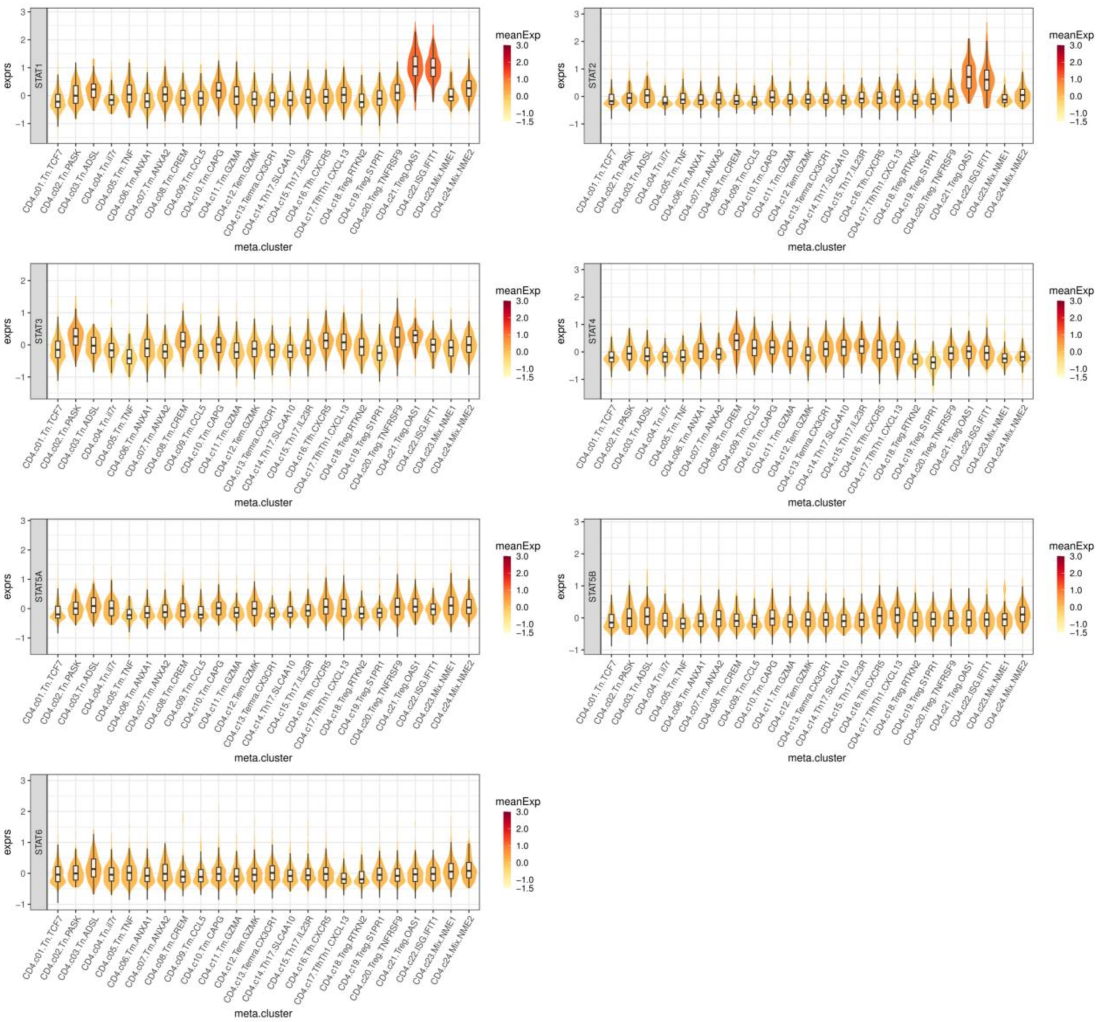

Figure S12 Supplementary figures for Figure 6b.

Violin plots revealing the distribution of STAT1 (a), STAT2 (b), STAT3 (c), STAT4 (d), STAT5A (e), STAT5B (f), and STAT6 (g) expression in different types of tumor infiltrating CD4+ T cells in pan-cancer, which revealed that STAT1 and STAT2 expression were notably up-regulated in CD4+ Treg cell with marker OSA1 and CD4+ T cell with ISG IFIT1.

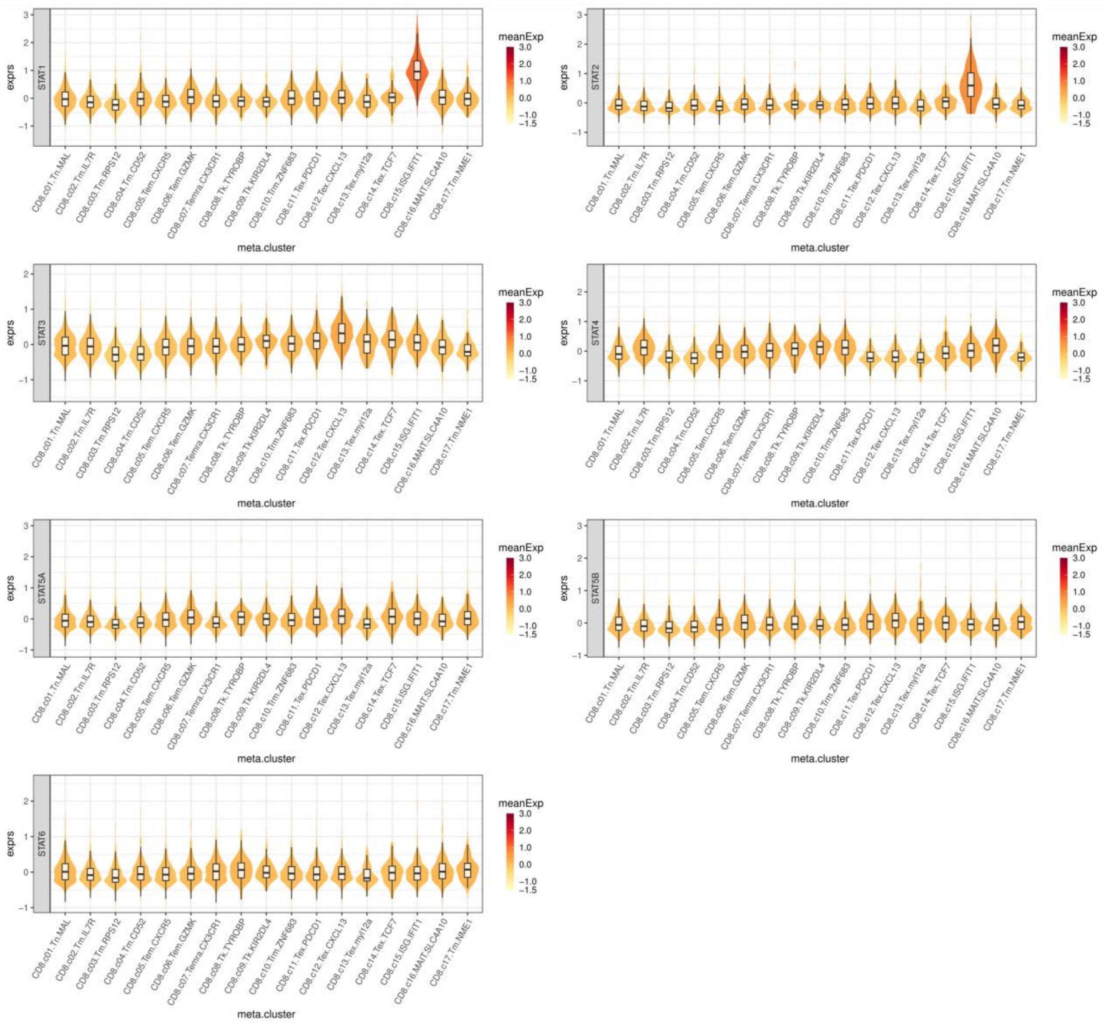

Figure S13 Supplementary figures for Figure 6c.

Violin plots demonstrating the distribution of STAT1 (a), STAT2 (b), STAT3 (c), STAT4 (d), STAT5A (e), STAT5B (f), and STAT6 (g) expression in different types of tumor infiltrating CD8+ T cells in pan-cancer, which illustrated that STAT1 and STAT2 had prominent up-regulation in CD8+ T cell with ISG IFIT1.

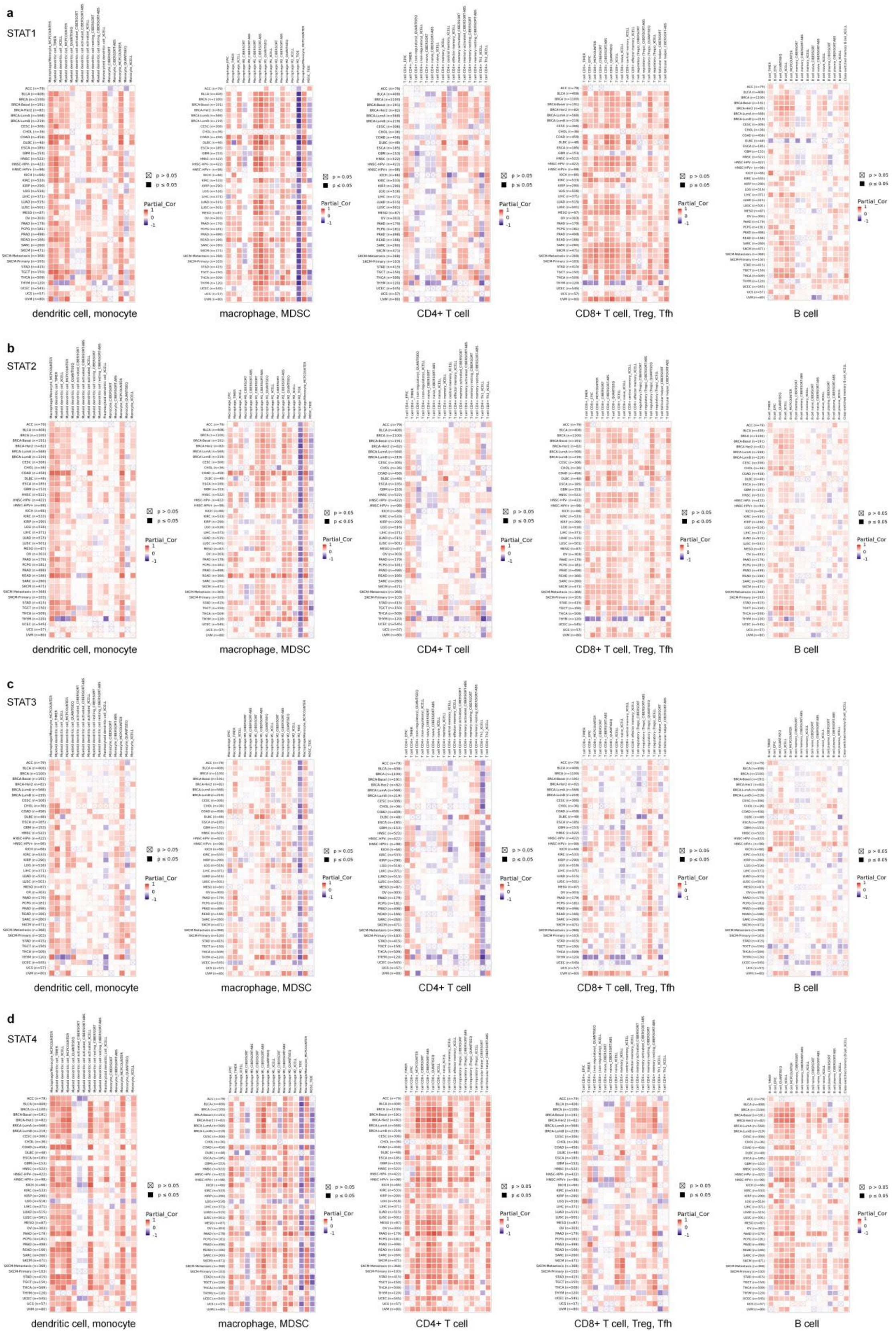

Figure S14 Immune infiltration analysis of STATs in pan-cancer.

Heatmaps depicting the correlation between STAT1 (a), STAT2 (b), STAT3 (c), and STAT4 (d) expression and different types of immune cells' infiltration in pan-cancer.

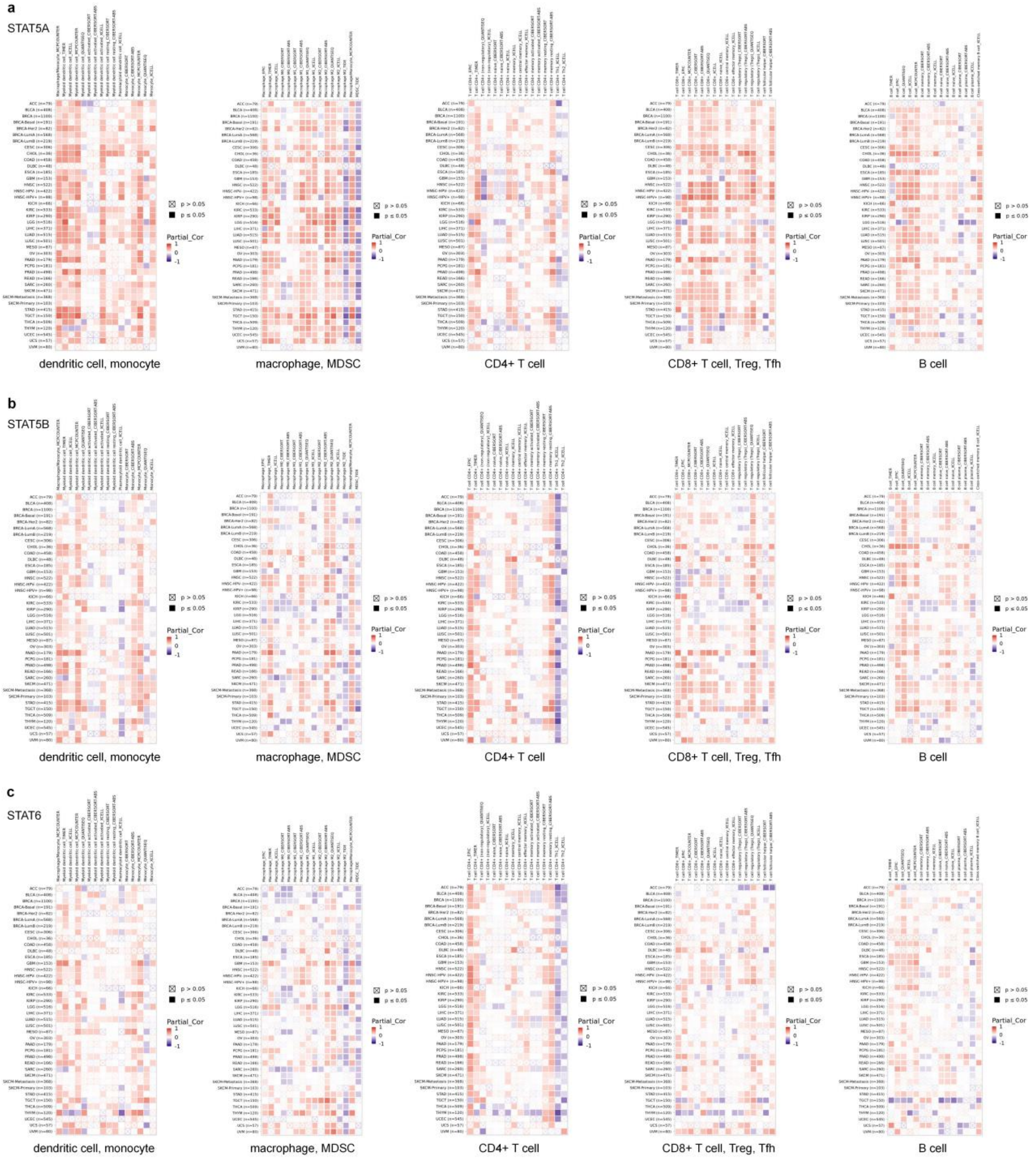

Figure S15 Immune infiltration analysis of STATs in pan-cancer.

Heatmaps illustrating the correlation between STAT5A (a), STAT5B (b), and STAT6 (c) expression and different types of immune cells' infiltration in pan-cancer, positive correlations were found in the infiltration of the majority of immune cells including dendritic cells, monocytes, macrophages, CD4+ T cells, CD8 T cells, and B cells, in which STAT1, STAT2, STAT4, and STAT5A were the most significant.

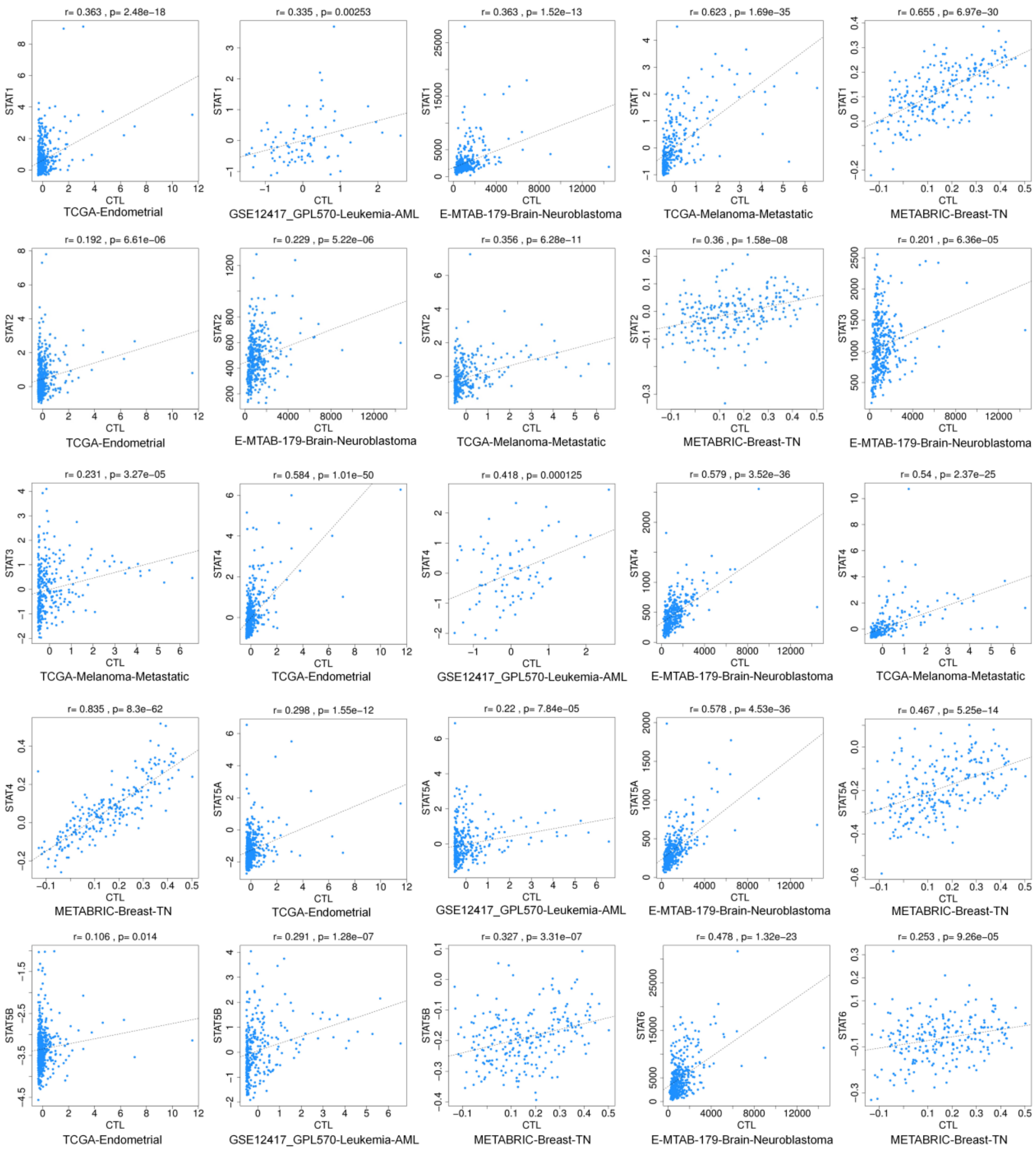

Figure S16 Immunotherapy related analysis of STATs in pan-cancer.

(a) STAT1 expression was revealed to be positively correlated with CTL infiltration levels in endometrial carcinoma (r = 0.363, *P* = 2.48e^{-18}), acute myeloid leukemia (r = 0.335, *P* = 2.53e^{-3}), neuroblastoma (r = 0.363, *P* = 1.52e^{-13}), metastatic melanoma (r = 0.623, *P* = 1.69e^{-35}), and triple negative breast cancer (r = 0.655, *P* = 6.97e^{-30}). (b) STAT2 expression was depicted to had positive correlation CTL infiltration levels in endometrial carcinoma (r = 0.192, *P* = 6.61e^{-6}), neuroblastoma (r = 0.229, *P* = 5.22e^{-6}), metastatic melanoma (r = 0.356, *P* = 6.28e^{-11}), and triple negative breast cancer (r = 0.360, *P* = 1.58e^{-8}). (c) STAT3 expression was revealed to be positively associated with CTL infiltration levels in neuroblastoma (r = 0.201, *P* = 6.36e^{-5}) and metastatic melanoma (r = 0.231, *P* = 3.27e^{-5}). (d) STAT4 expression was characterized to had positive correlation with CTL infiltration levels in endometrial carcinoma (r = 0.584, *P* = 1.01e^{-50}), acute myeloid leukemia (r = 0.418, *P* = 1.25e^{-4}), neuroblastoma (r = 0.579, *P* = 3.52e^{-36}), metastatic melanoma (r = 0.540, *P* = 2.37e^{-25}), and triple negative breast cancer (r = 0.835, *P* = 8.30e^{-62}). (e) STAT5A expression was characterized to be positively linked with CTL infiltration

levels in endometrial carcinoma (r = 0.298, P = 1.55e^{-12}), acute myeloid leukemia (r = 0.220, P = 7.84e^{-5}), neuroblastoma (r = 0.578, P = 4.53e^{-36}), and triple negative breast cancer (r = 0.467, P = 5.25e^{-14}). (f) STAT5B expression was displayed to be positively correlated with CTL infiltration levels in endometrial carcinoma (r = 0.106, P =1.40e^{-2}), acute myeloid leukemia (r = 0.291, P = 1.28e^{-7}), and triple negative breast cancer (r = 0.327, P = 3.31e^{-7}). (g) STAT6 expression was shown to be positively linked with CTL infiltration levels in neuroblastoma (r = 0.478, P =1.32e^{-23}) and triple negative breast cancer (r = 0.253, P = 9.26e^{-5}).

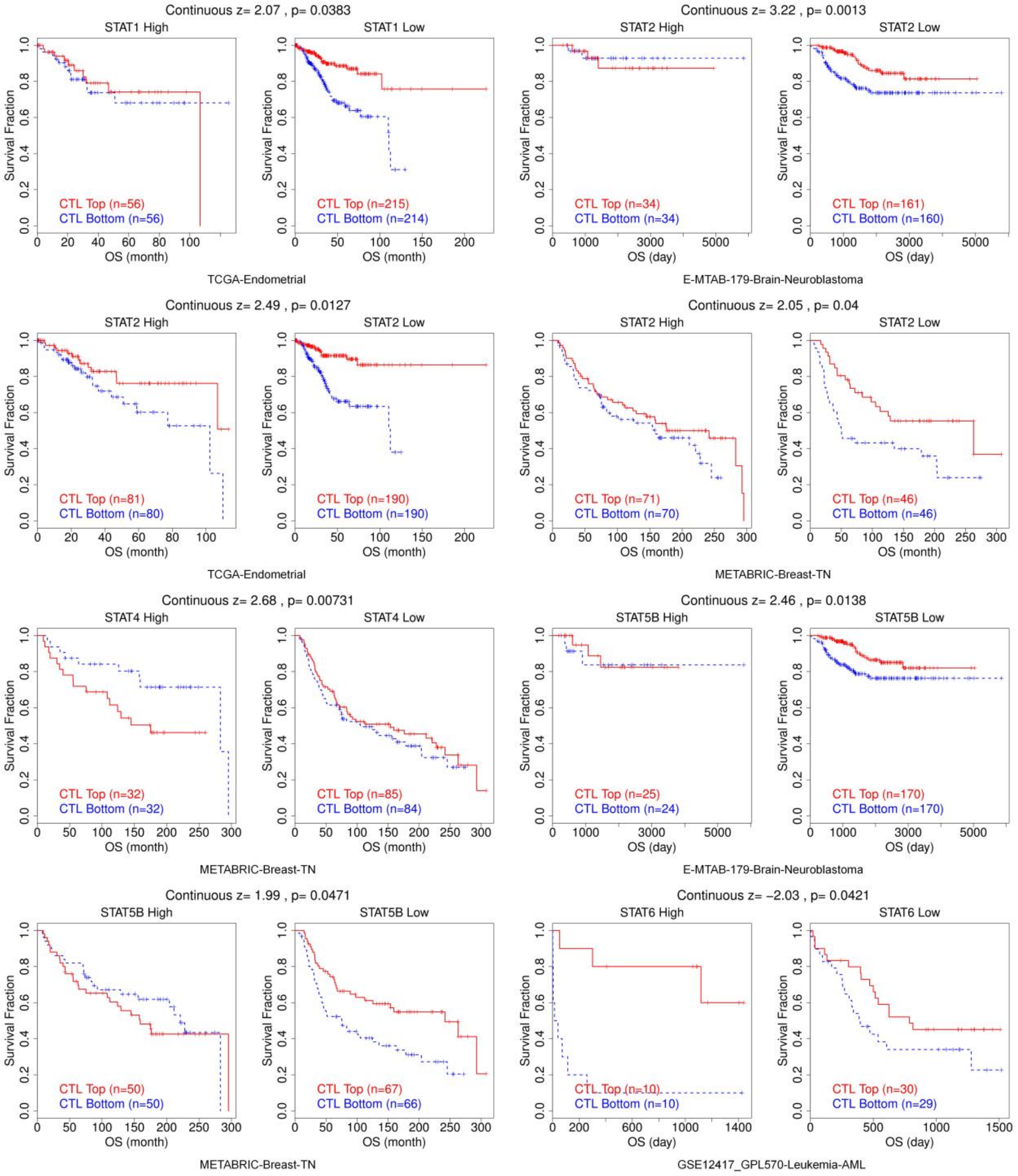

Figure S17 Immunotherapy related analysis of STATs in pan-cancer.

(a) A higher CTL level indicated a better prognosis when STAT1 had relatively low expression in endometrial carcinoma (z score = 2.07, P = 0.0383). (b) Higher CTL levels suggested more favorable survival outcomes when STAT2 had relatively low expression in neuroblastoma (z score = 3.22, P = 0.0013). (c) Higher CTL levels suggested more favorable survival outcomes when STAT2 had relatively low expression in endometrial carcinoma (z score = 2.49, P = 0.0127). (d)

Higher CTL levels suggested more favorable survival outcomes when STAT2 had relatively low expression in triple negative breast cancer (z score = 2.05, *P* = 0.04). (e) A lower CTL level indicated a better survival outcome when STAT4 were high expressed in triple negative breast cancer (z score = 2.68, *P* = 0.00731). (f) Escalated CTL levels suggested more favorable prognosis when STAT5B had relatively low expression in neuroblastoma (z score = 2.46, *P* = 0.0138). (g) Escalated CTL levels suggested more favorable prognosis when STAT5B had relatively low expression in triple negative breast cancer (z score = 1.99, *P* = 0.0471). (h) A higher CTL level indicated a better prognosis when STAT6 had higher expression in acute myeloid leukemia (z score = -2.03, *P* = 0.0421).

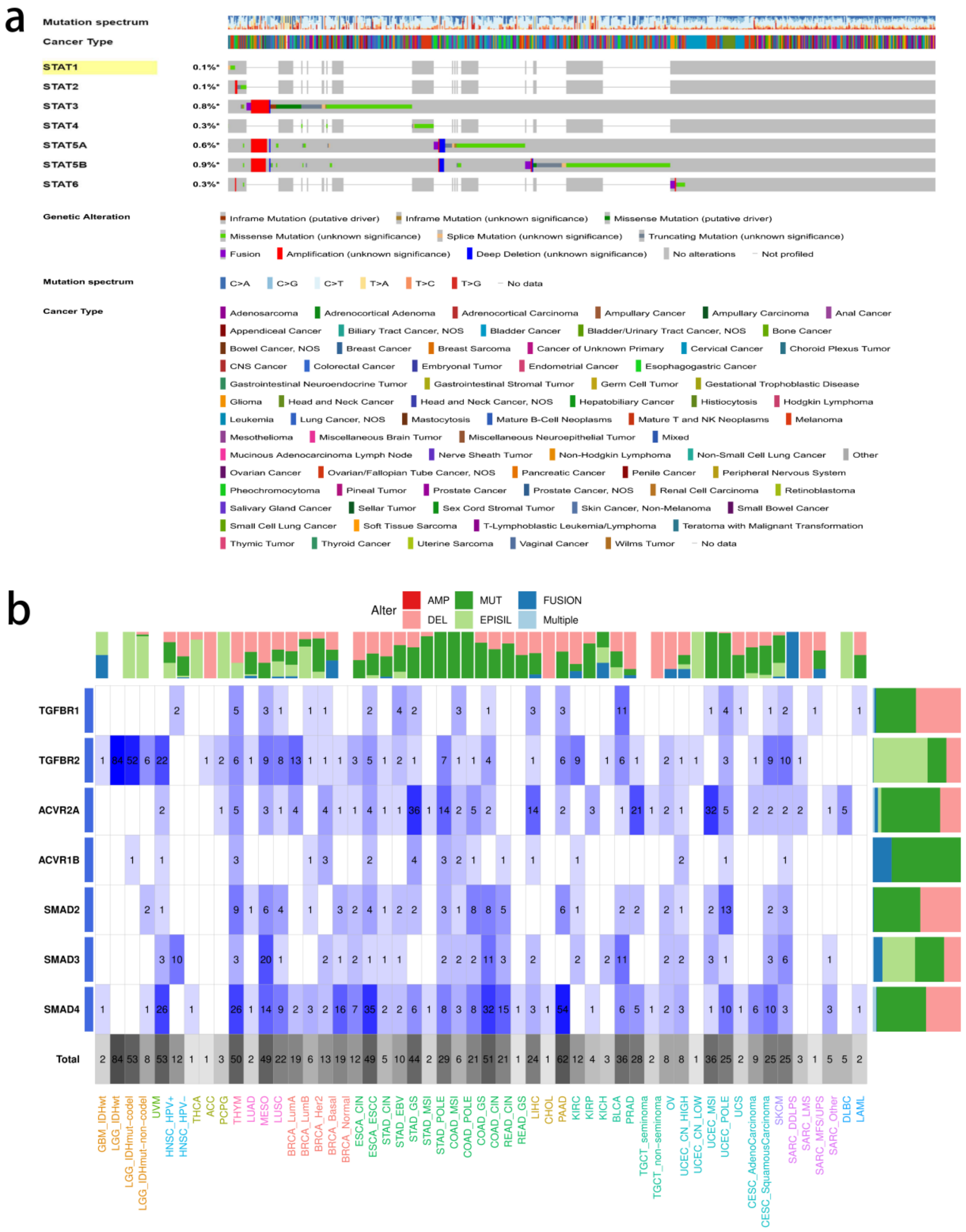

Figure S18 Tumor mutation related analysis of STATs in pan-cancer.

(a) An oncoprint illustrated the mutations of STATs from Cbioportal, which showed that STATs had multiple types but rather low frequencies of genetic alteration in various malignancies. (b) Detailed heatmap of mutation frequencies of relevant genes on recognized signaling transduction pathways with STATs including TGFBR1, TGFBR2, ACVR2A, ACVR1B, SMAD2, SMAD3, and SMAD4 were also analyzed.
