# Supplementary material for: Pan-cancer analysis reveals signal transducer and activator of transcription (STAT) gene family as biomarkers for prognostic prediction and therapeutic guidance
Source: Front Genet. 2023 Mar 9;14:1120500. doi: 10.3389/fgene.2023.1120500 (PMC10034013; doi:10.3389/fgene.2023.1120500)
Supplement: Supplementary file 2 [file DataSheet1.ZIP › Source data/drugCor.pdf]

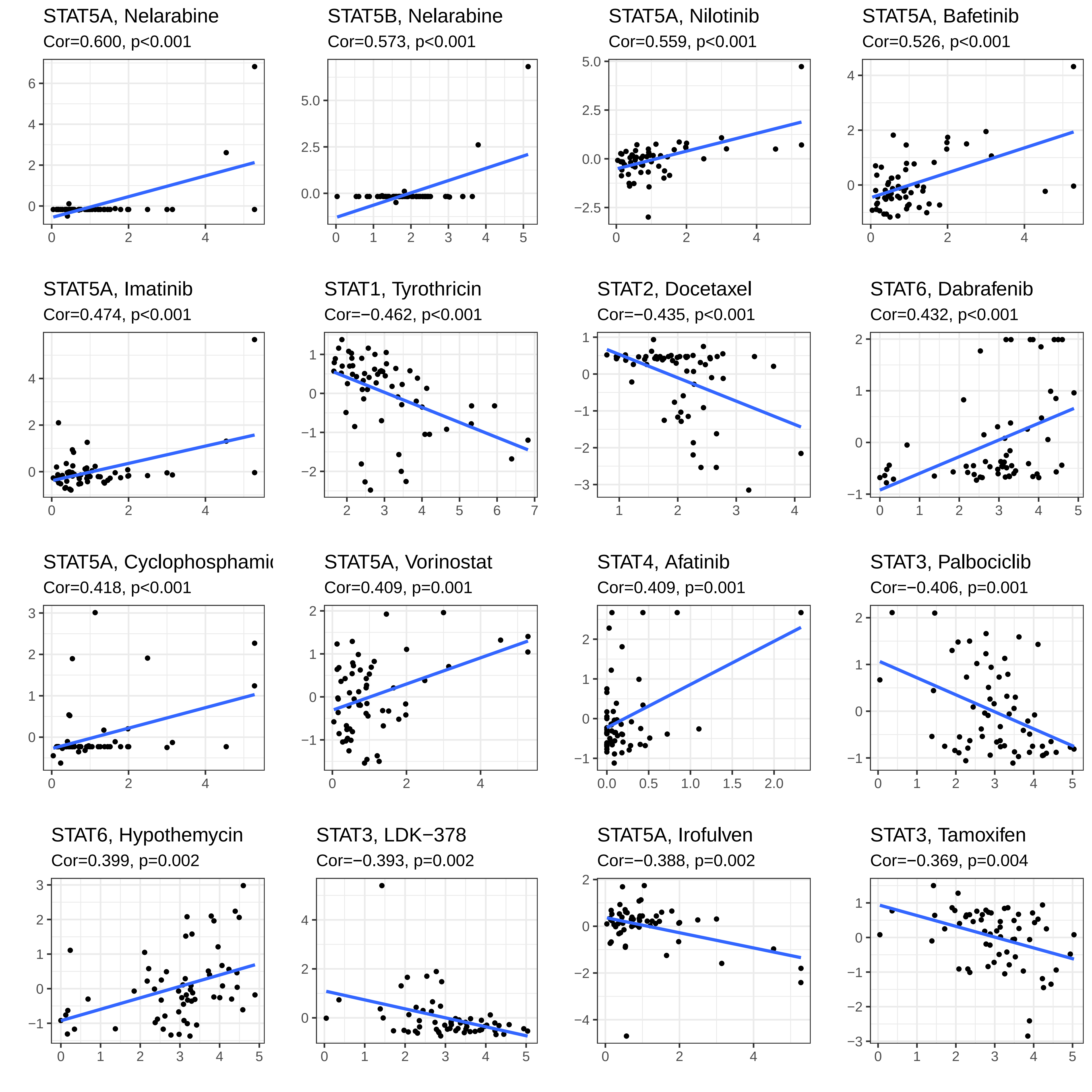

STAT5A, Nelarabine
Cor=0.600, p<0.001
STAT5B, Nelarabine
Cor=0.573, p<0.001
STAT5A, Nilotinib
Cor=0.559, p<0.001
STAT5A, Bafetinib
Cor=0.526, p<0.001
STAT5A, Imatinib
Cor=0.474, p<0.001
STAT1, Tyrothricin
Cor=−0.462, p<0.001
STAT2, Docetaxel
Cor=−0.435, p<0.001
STAT6, Dabrafenib
Cor=0.432, p<0.001
STAT5A, Cyclophosphami
Cor=0.418, p<0.001
STAT5A, Vorinostat
Cor=0.409, p=0.001
STAT4, Afatinib
Cor=0.409, p=0.001
STAT3, Palbociclib
Cor=−0.406, p=0.001
STAT6, Hypothemycin
Cor=0.399, p=0.002
STAT3, LDK−378
Cor=−0.393, p=0.002
STAT5A, Irofulven
Cor=−0.388, p=0.002
STAT3, Tamoxifen
Cor=−0.369, p=0.004
