# Supplementary figures and images for: Pan-cancer analysis reveals signal transducer and activator of transcription (STAT) gene family as biomarkers for prognostic prediction and therapeutic guidance
Source: Front Genet. 2023 Mar 9;14:1120500. doi: 10.3389/fgene.2023.1120500 (PMC10034013; doi:10.3389/fgene.2023.1120500)

# Cancer: BRCA

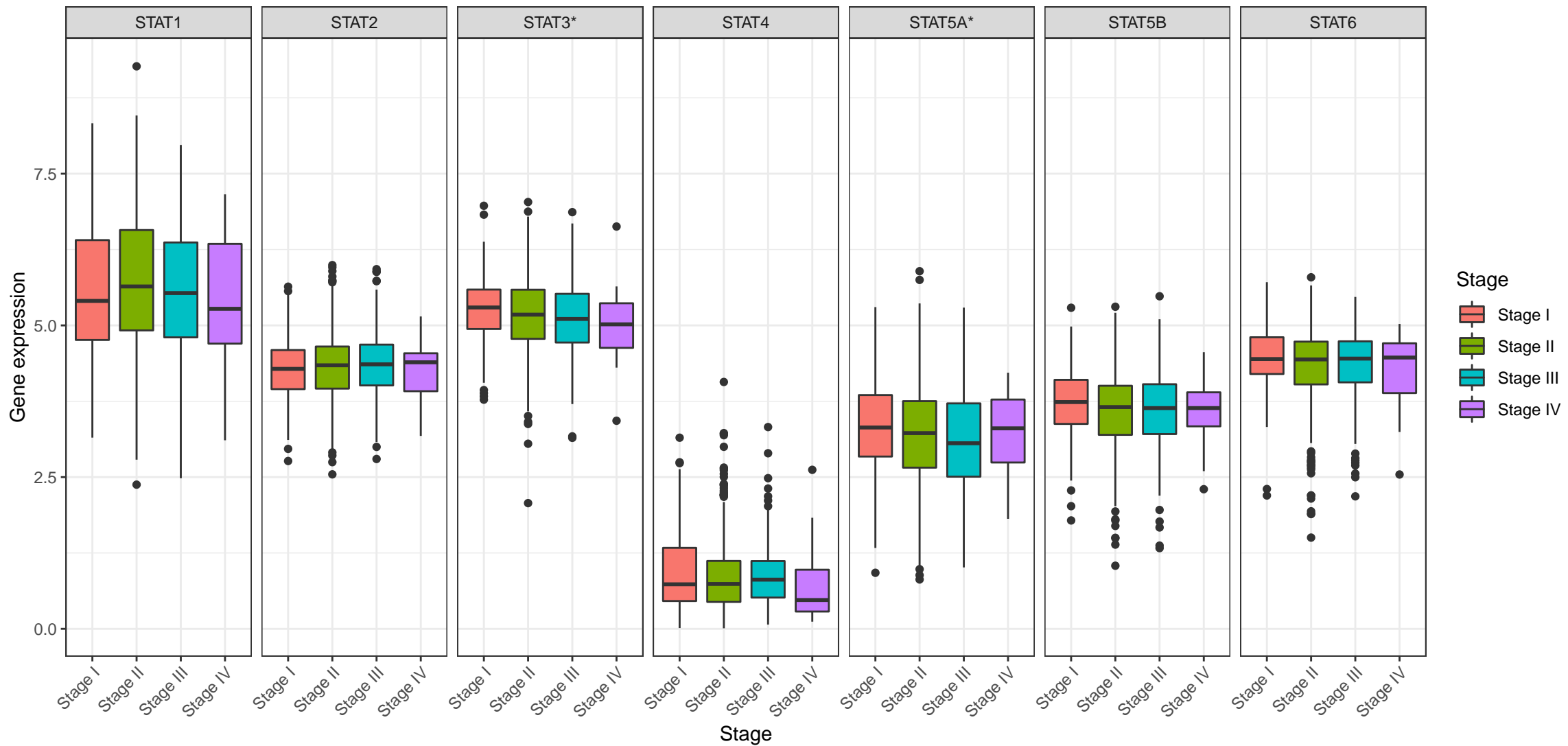

Supplement: Supplementary file 2 [file DataSheet1.ZIP › Source data/BRCA cliCor.pdf]

# Cancer: BRCA

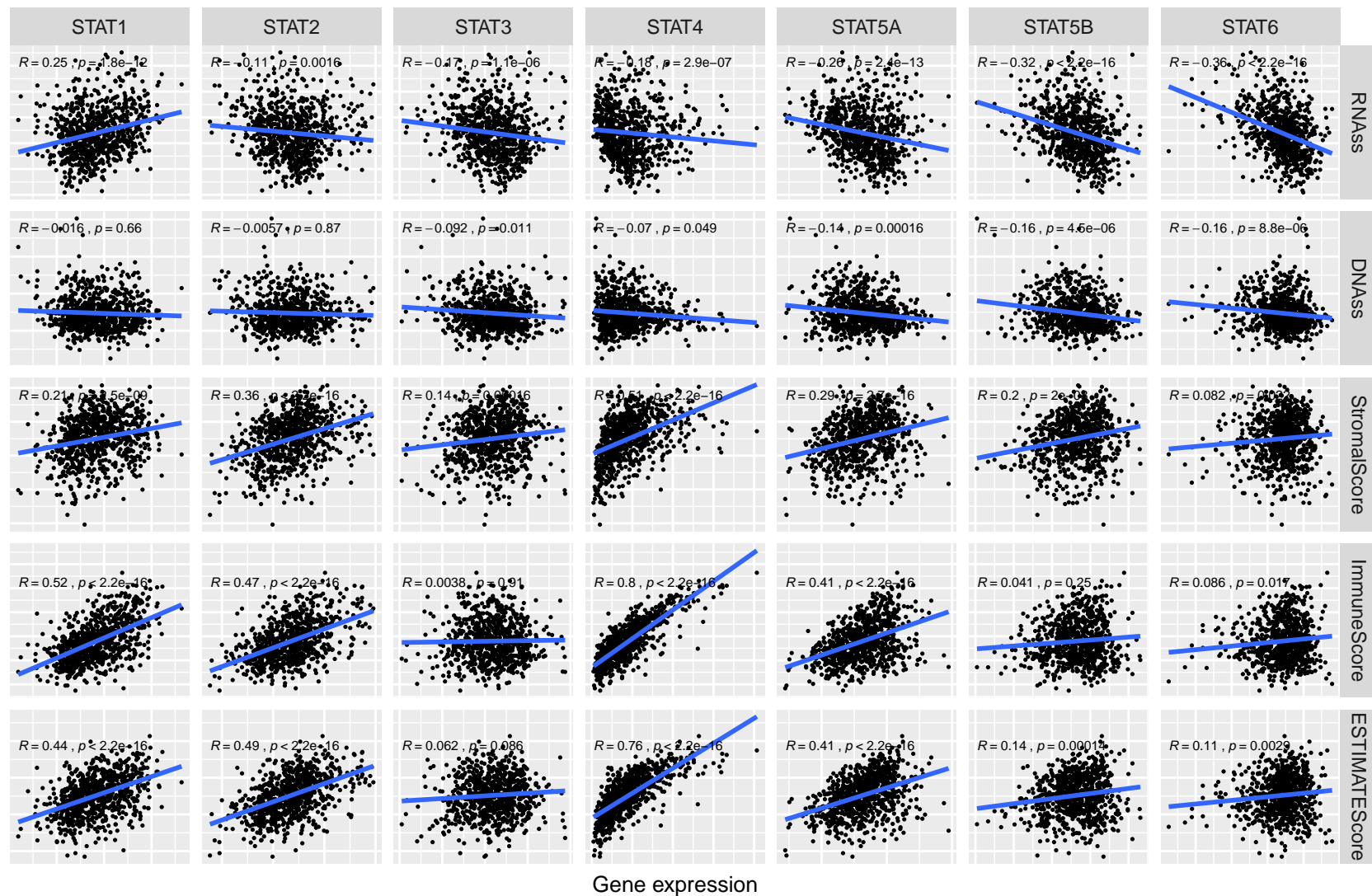

Supplement: Supplementary file 2 [file DataSheet1.ZIP › Source data/BRCA cor.pdf]

# Cancer: COAD

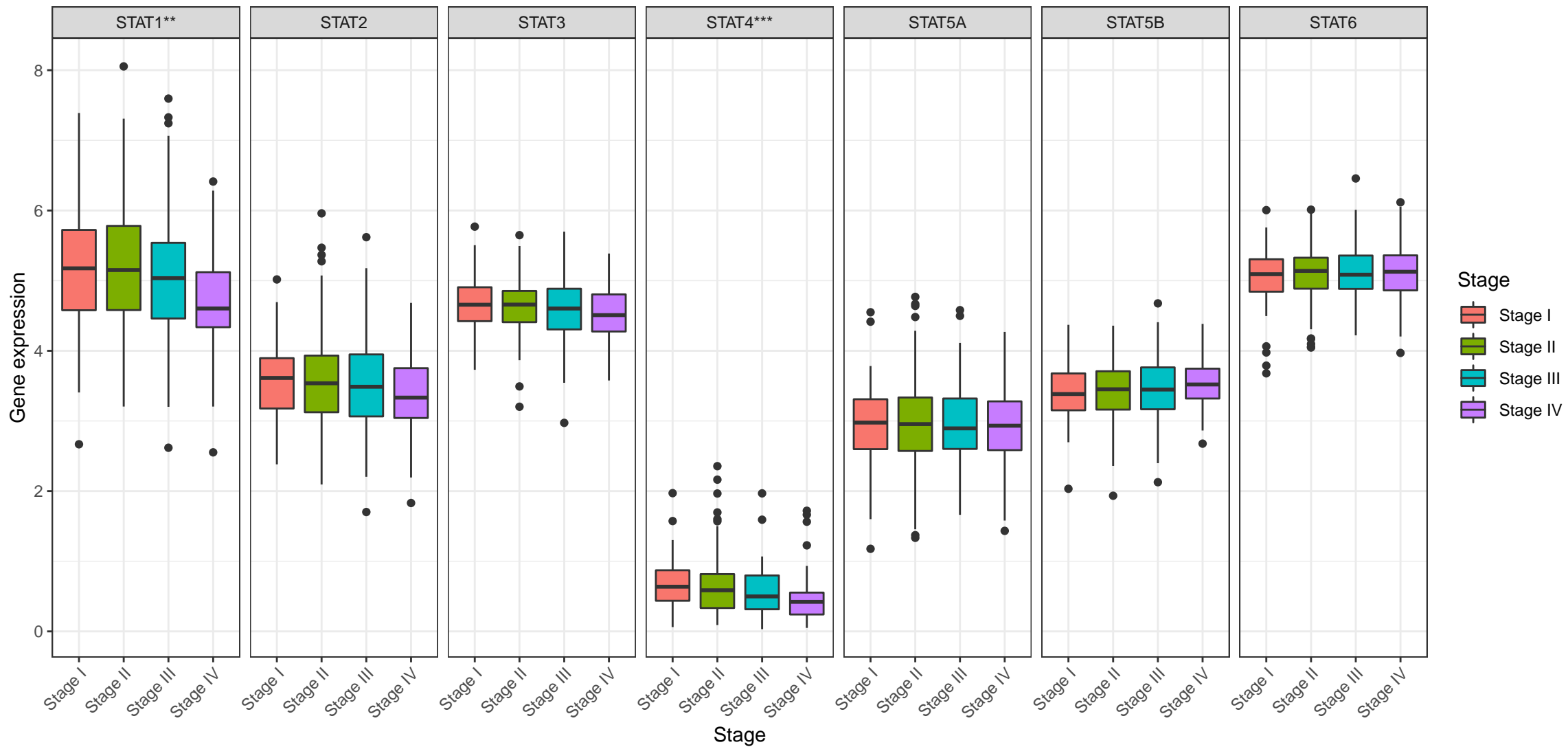

Supplement: Supplementary file 2 [file DataSheet1.ZIP › Source data/COAD cliCor.pdf]

# Cancer: COAD

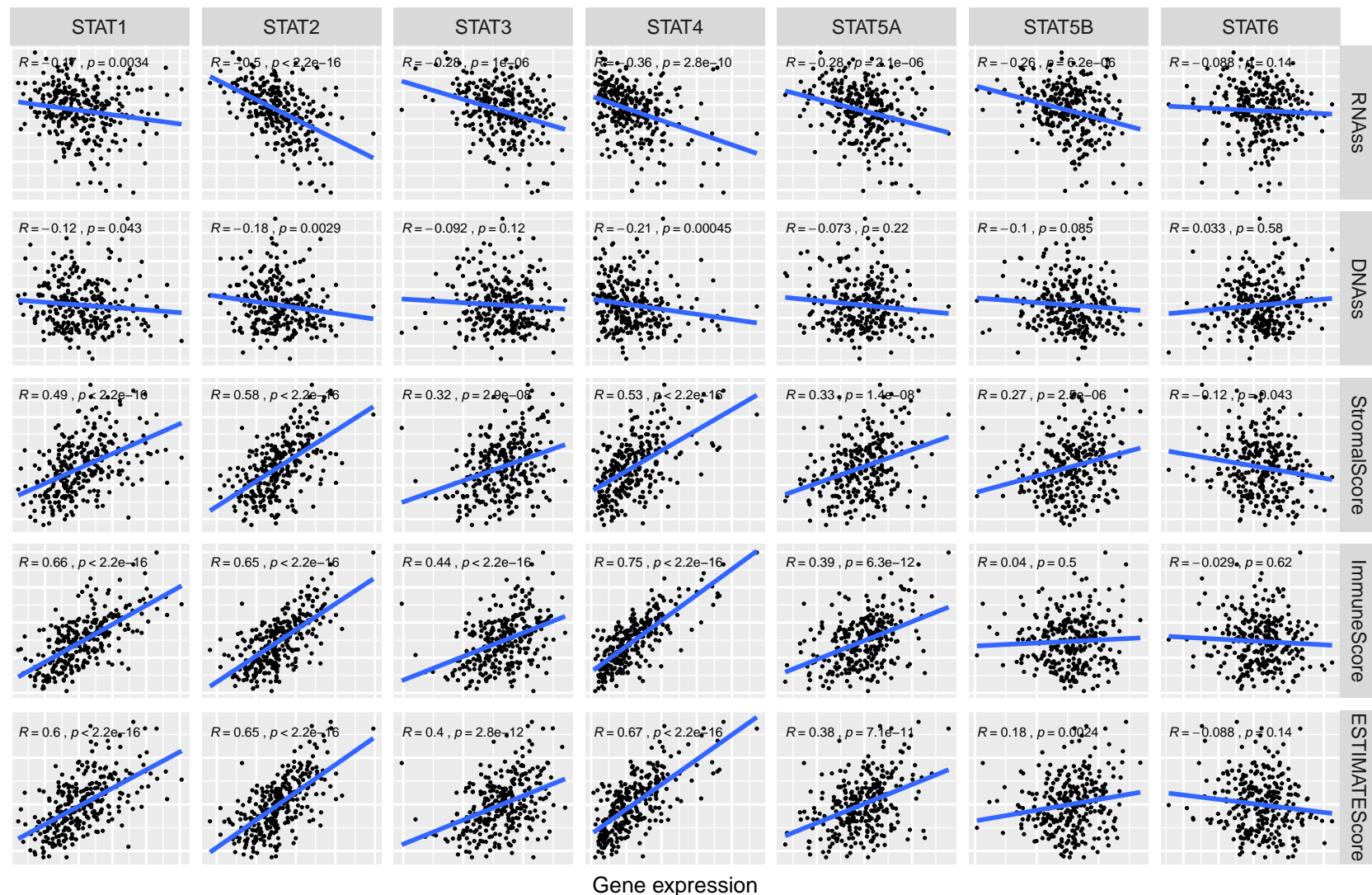

Supplement: Supplementary file 2 [file DataSheet1.ZIP › Source data/COAD cor.pdf]

# DNAss

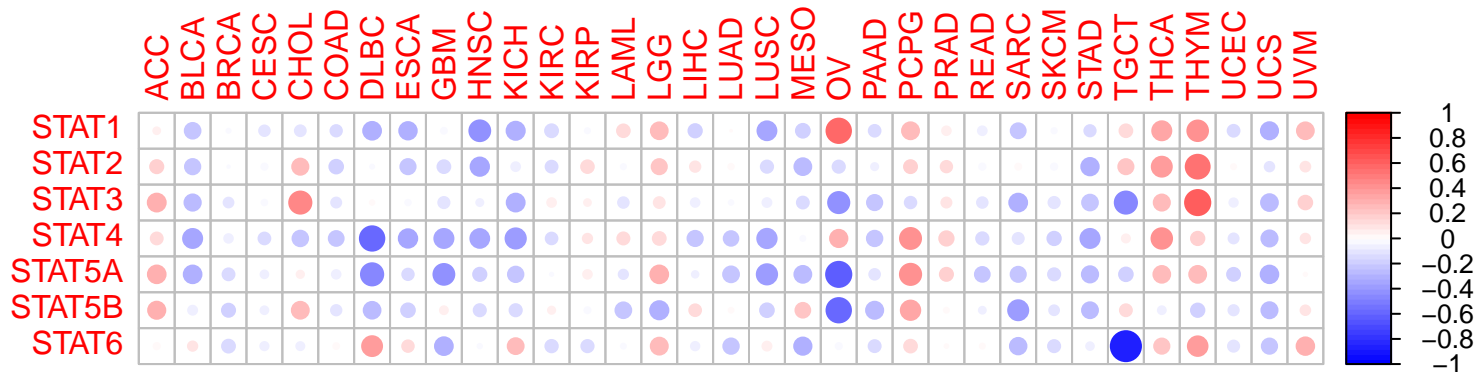

Supplement: Supplementary file 2 [file DataSheet1.ZIP › Source data/DNAssCor.pdf]

# ESTIMATEScore

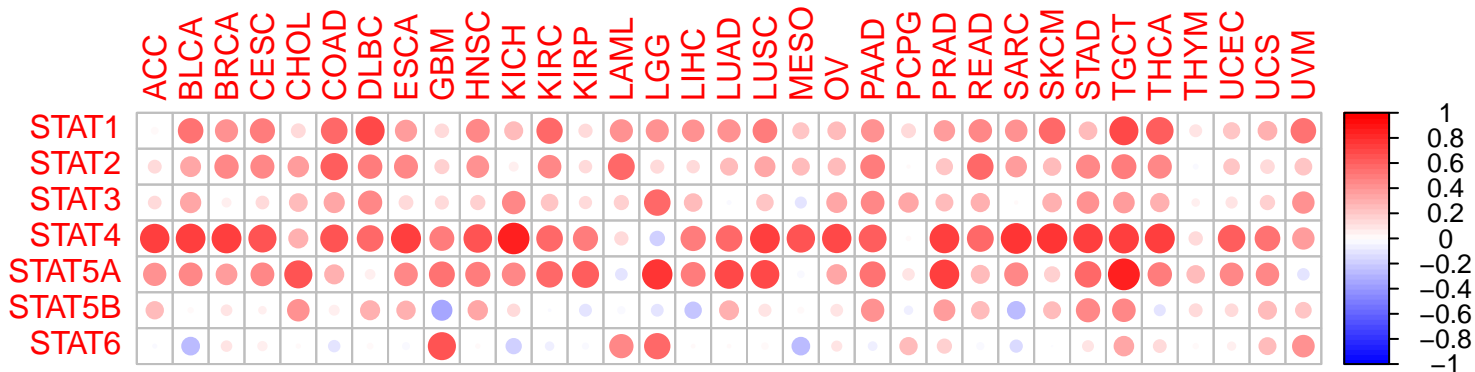

Supplement: Supplementary file 2 [file DataSheet1.ZIP › Source data/ESTIMATEScore.pdf]

Cancer: HNSC

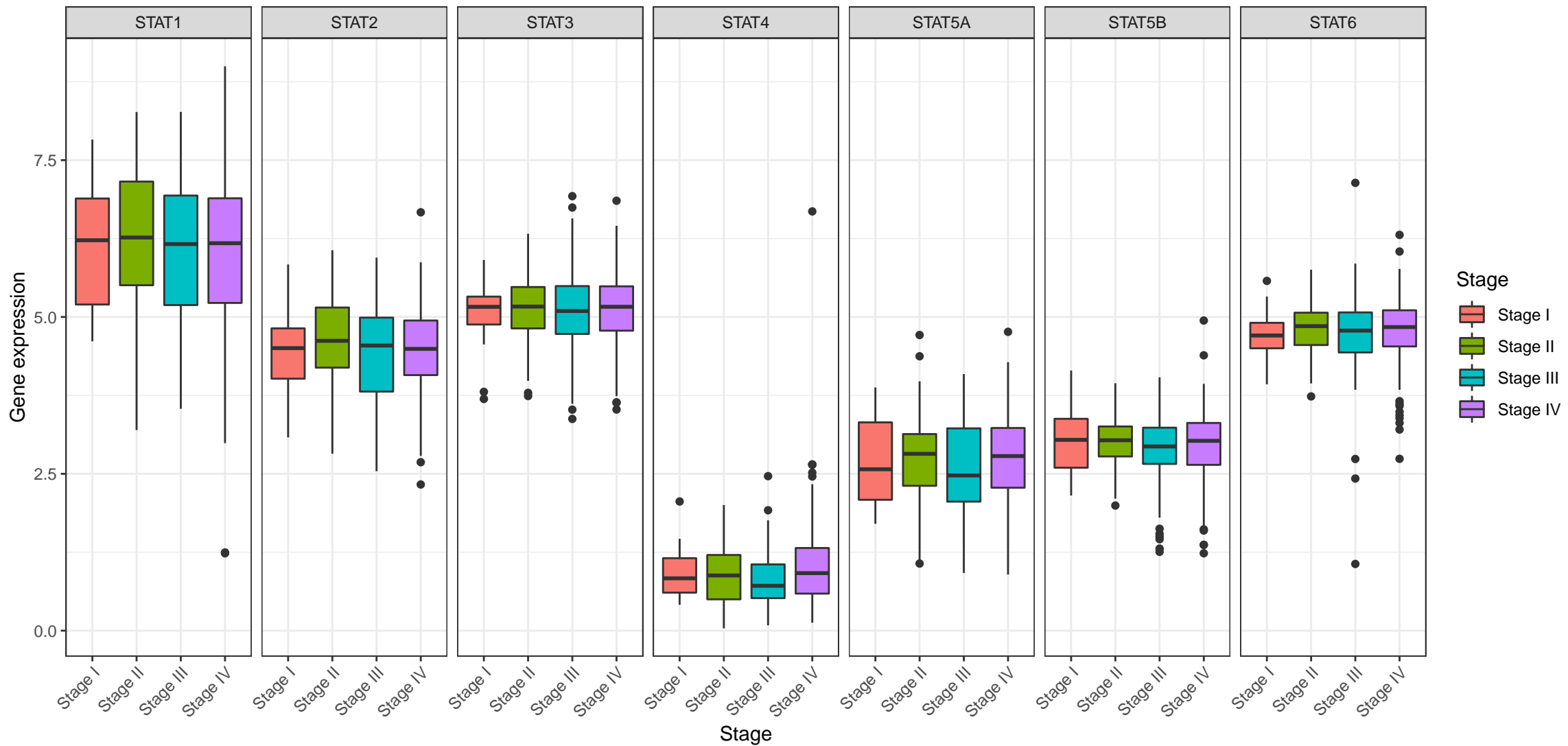

Supplement: Supplementary file 2 [file DataSheet1.ZIP › Source data/HNSC cliCor.pdf]

# Cancer: HNSC

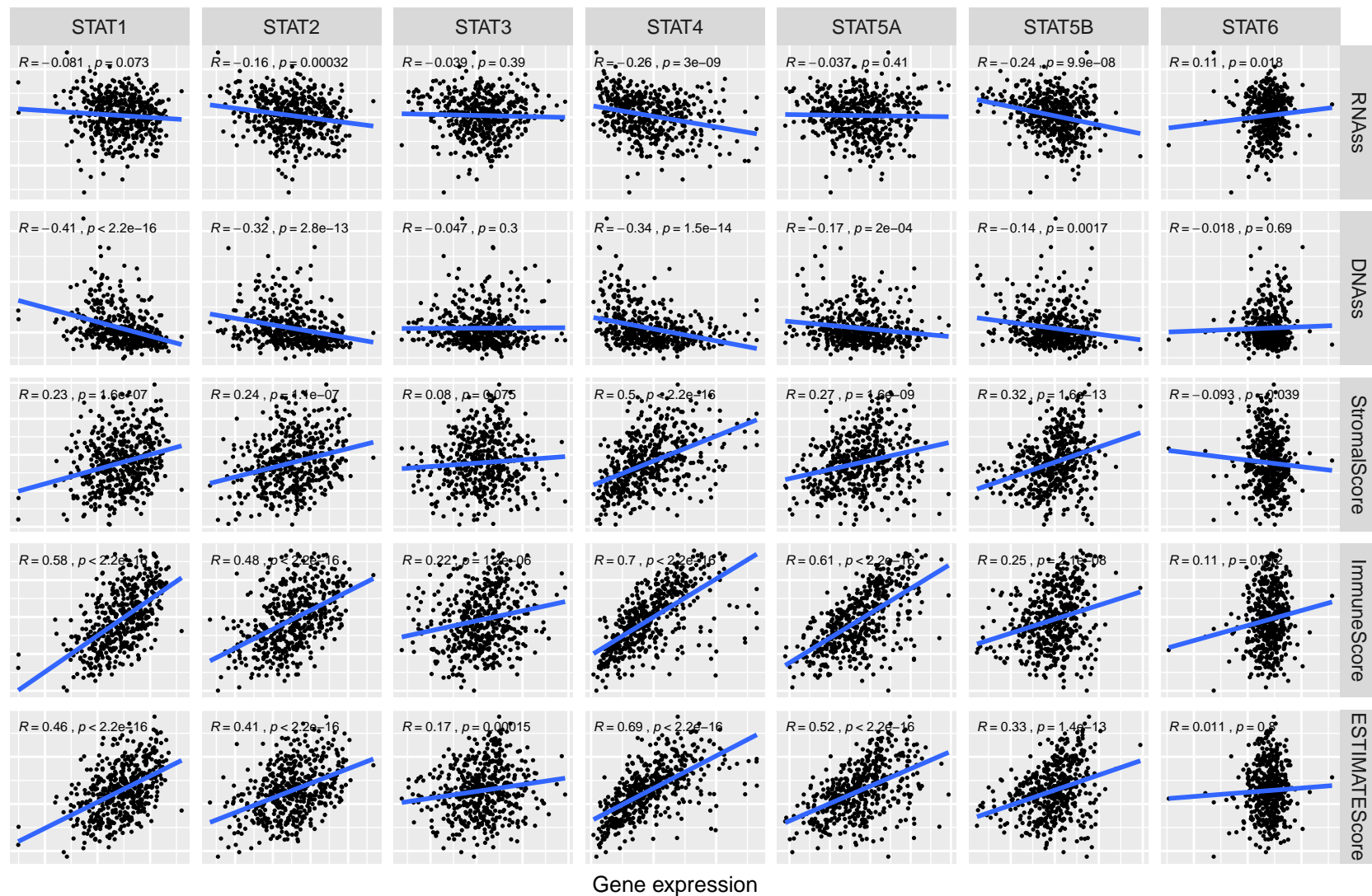

Supplement: Supplementary file 2 [file DataSheet1.ZIP › Source data/HNSC cor.pdf]

# ImmuneScore

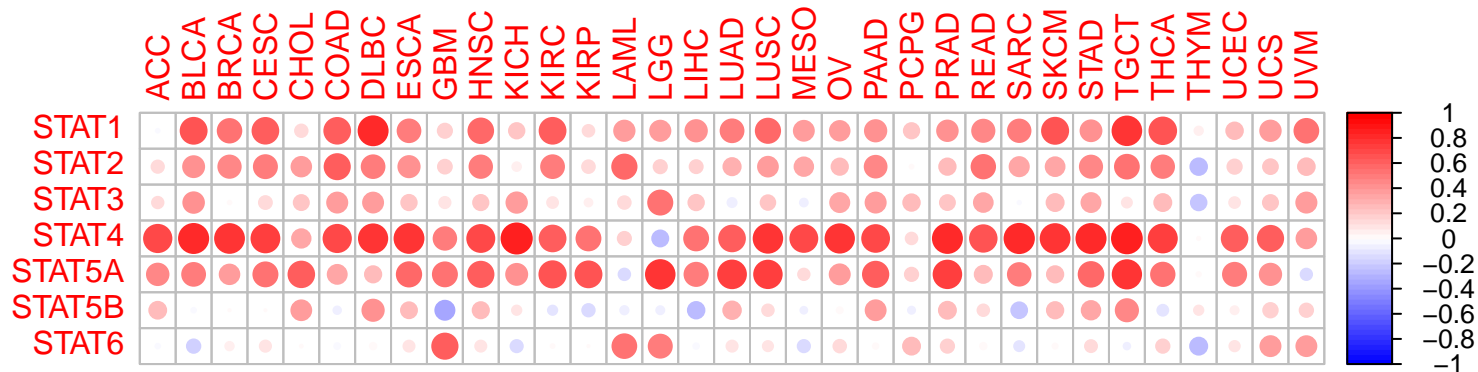

Supplement: Supplementary file 2 [file DataSheet1.ZIP › Source data/ImmuneScore.pdf]

# Cancer: LIHC

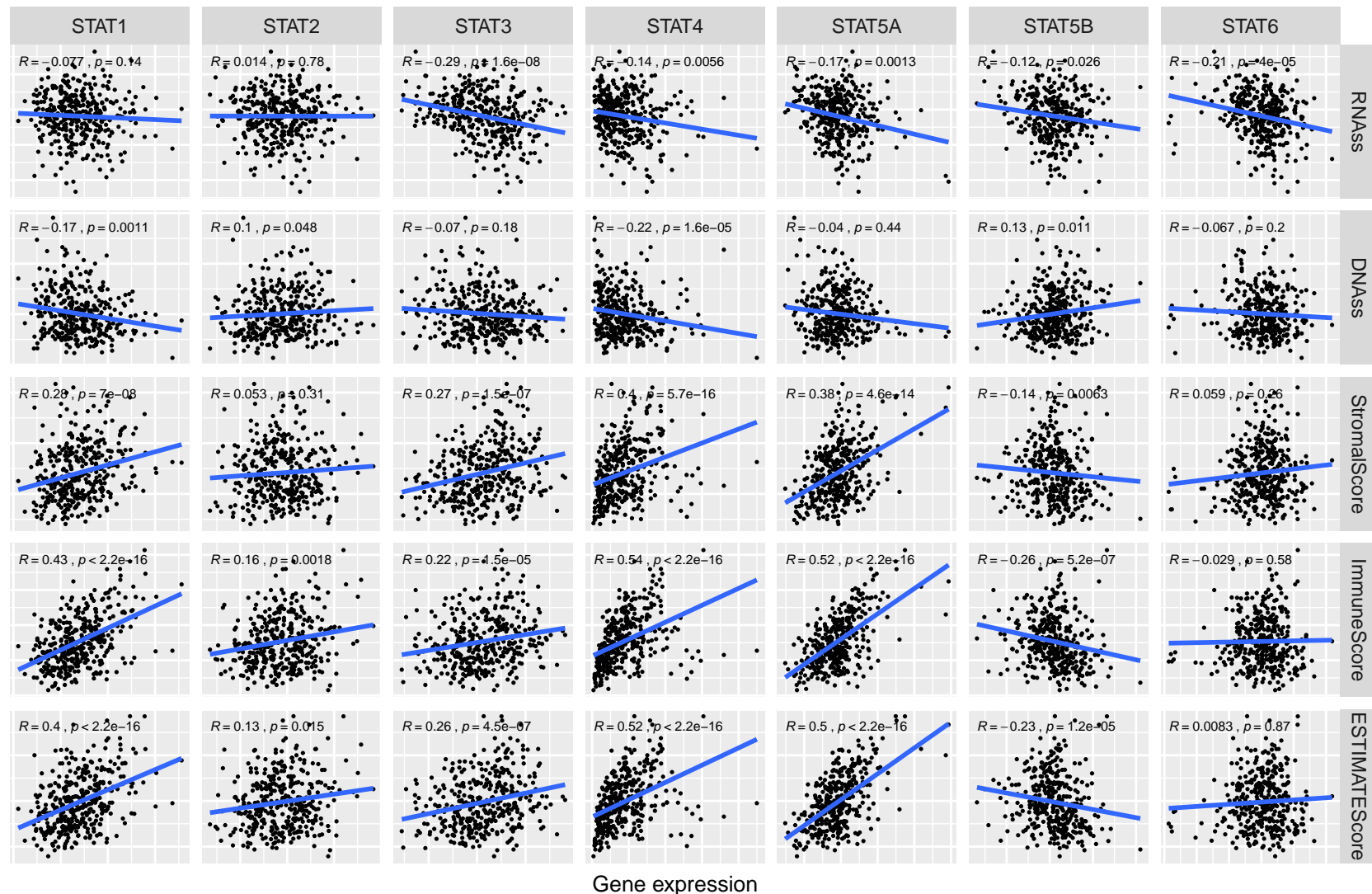

Supplement: Supplementary file 2 [file DataSheet1.ZIP › Source data/LIHC cor.pdf]

# Cancer: LIHC

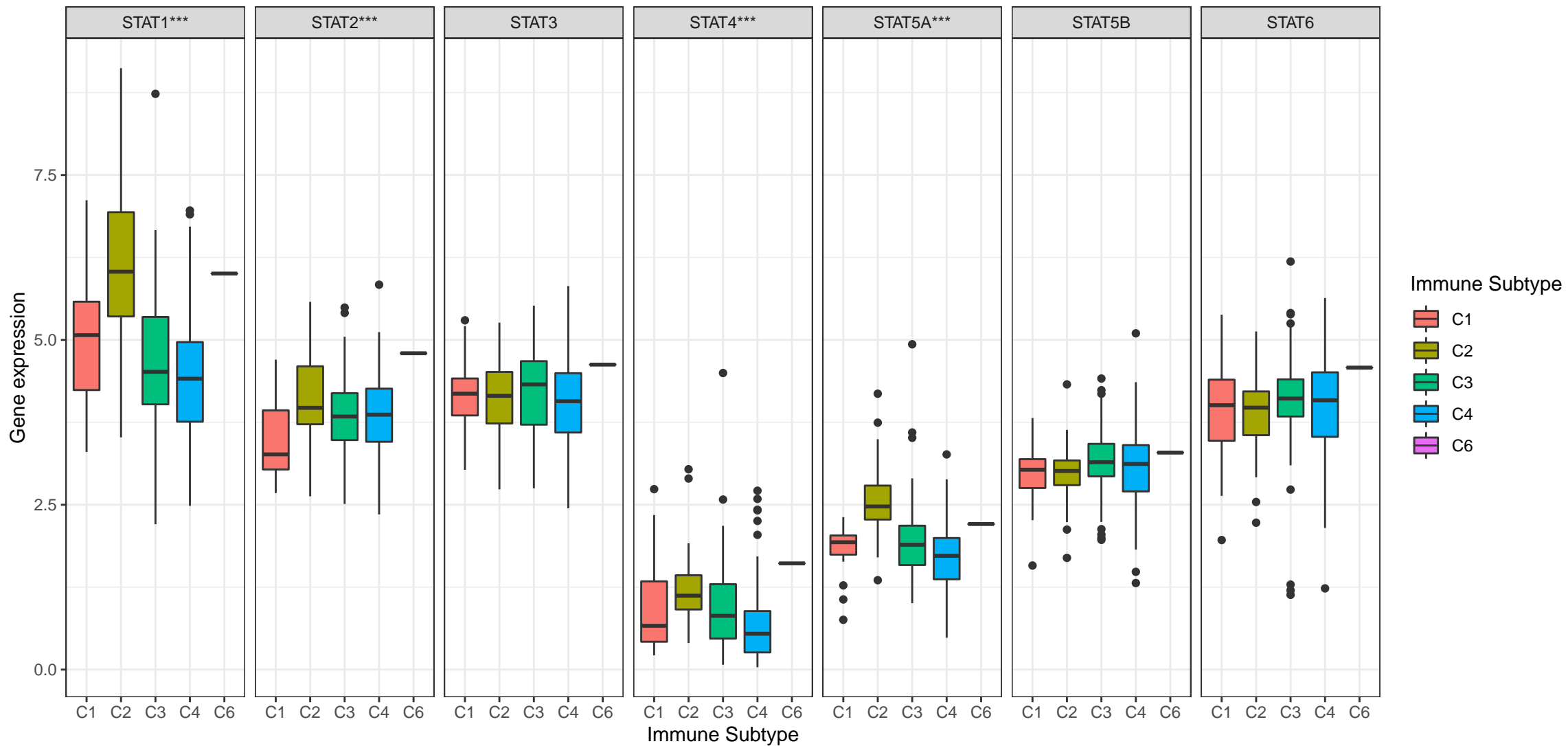

Supplement: Supplementary file 2 [file DataSheet1.ZIP › Source data/LIHC immuneType.pdf]

# Cancer: LUAD

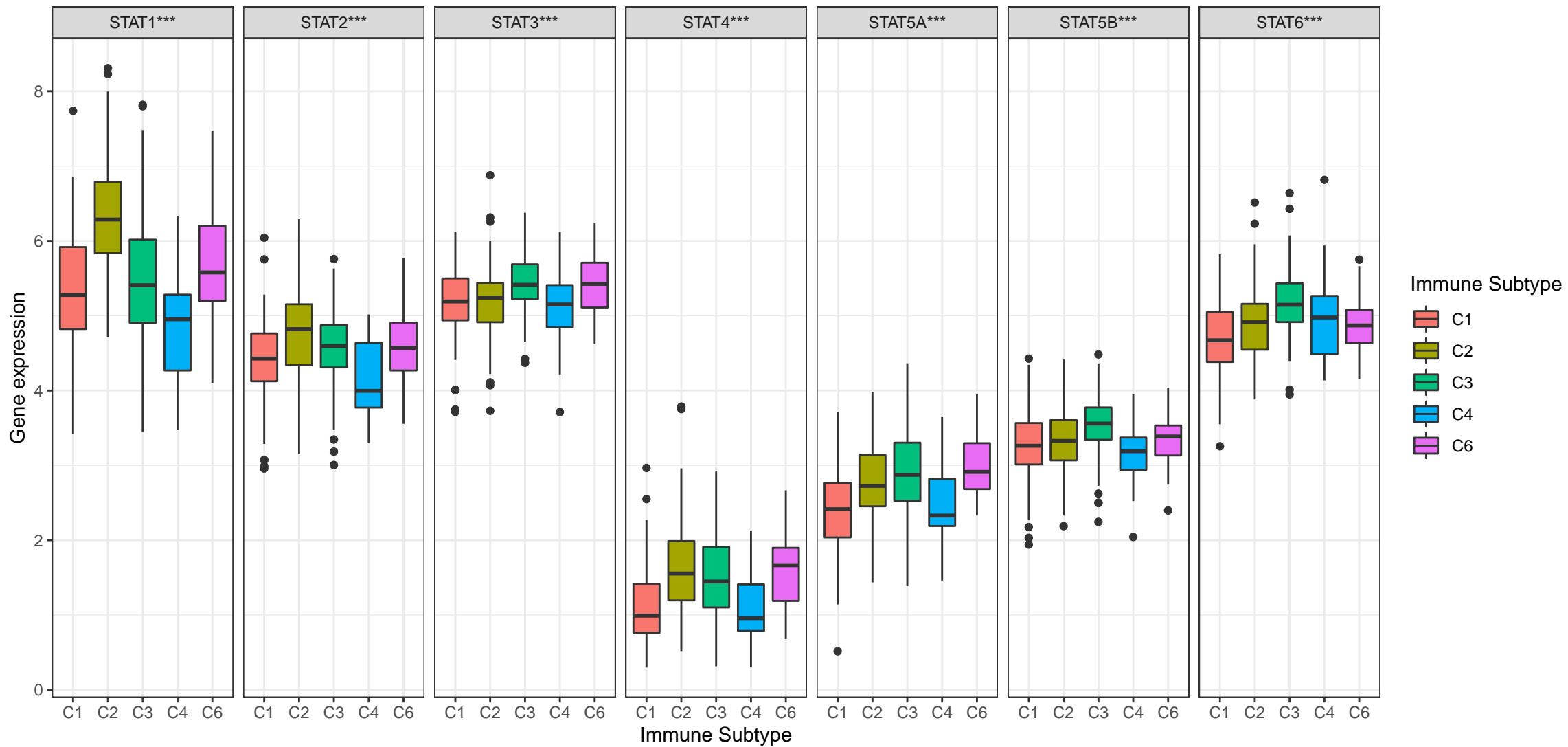

Supplement: Supplementary file 2 [file DataSheet1.ZIP › Source data/LUAD immuneType.pdf]

# RNAss

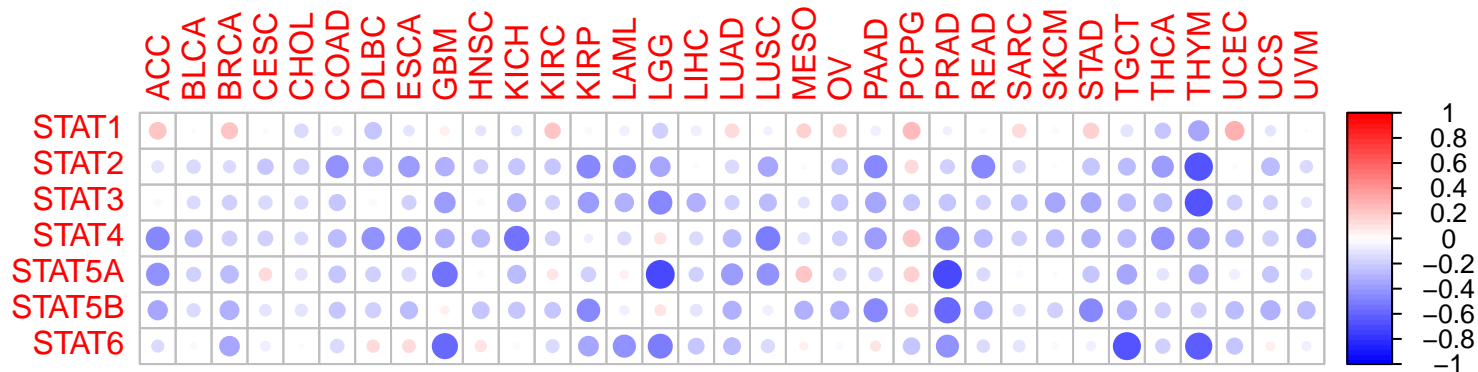

Supplement: Supplementary file 2 [file DataSheet1.ZIP › Source data/RNAssCor.pdf]

Cancer: SARC

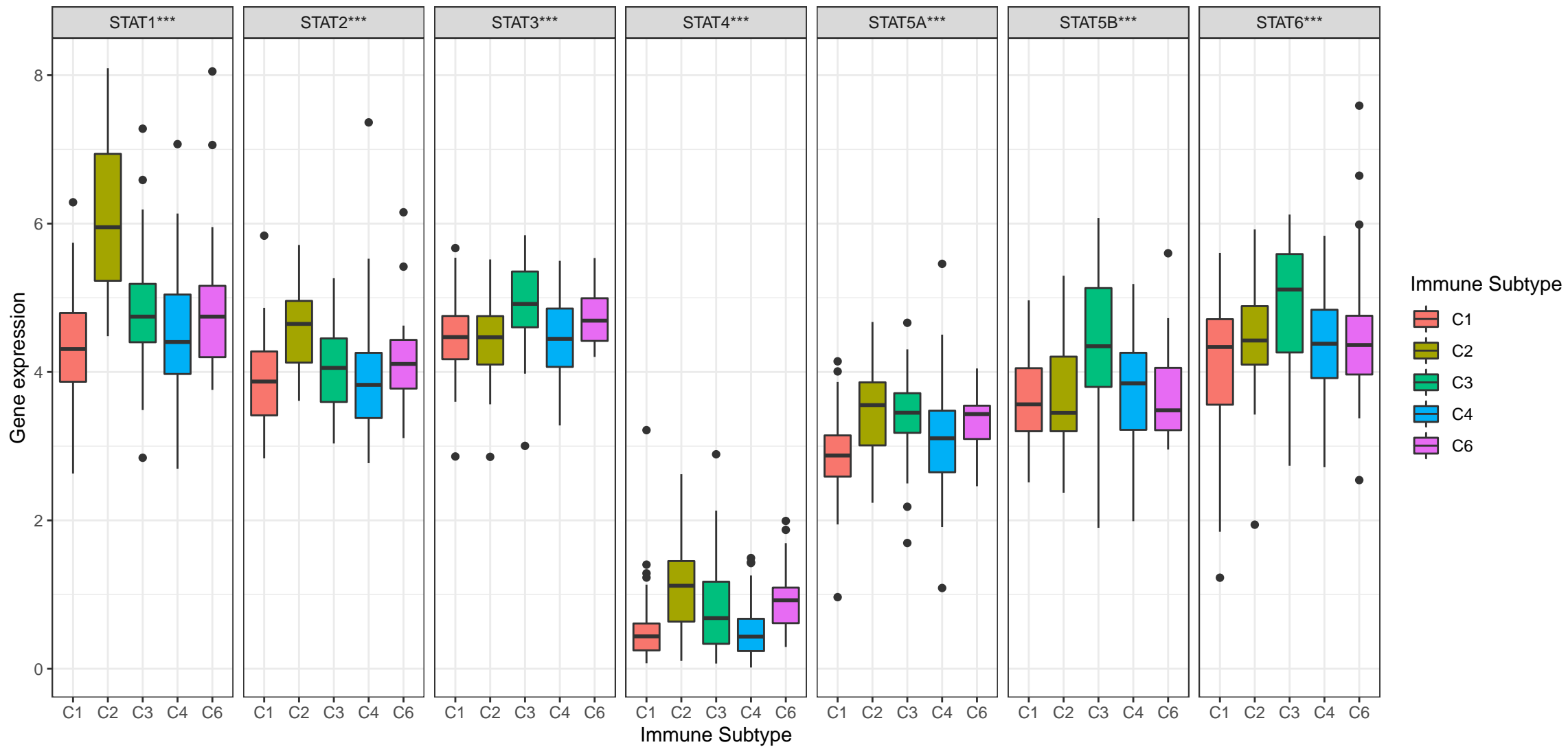

Supplement: Supplementary file 2 [file DataSheet1.ZIP › Source data/SARC immuneType.pdf]

STAT1\*\*\*

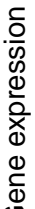

Supplement: Supplementary file 2 [file DataSheet1.ZIP › Source data/SKCM immuneType.pdf]

Cancer: STAD

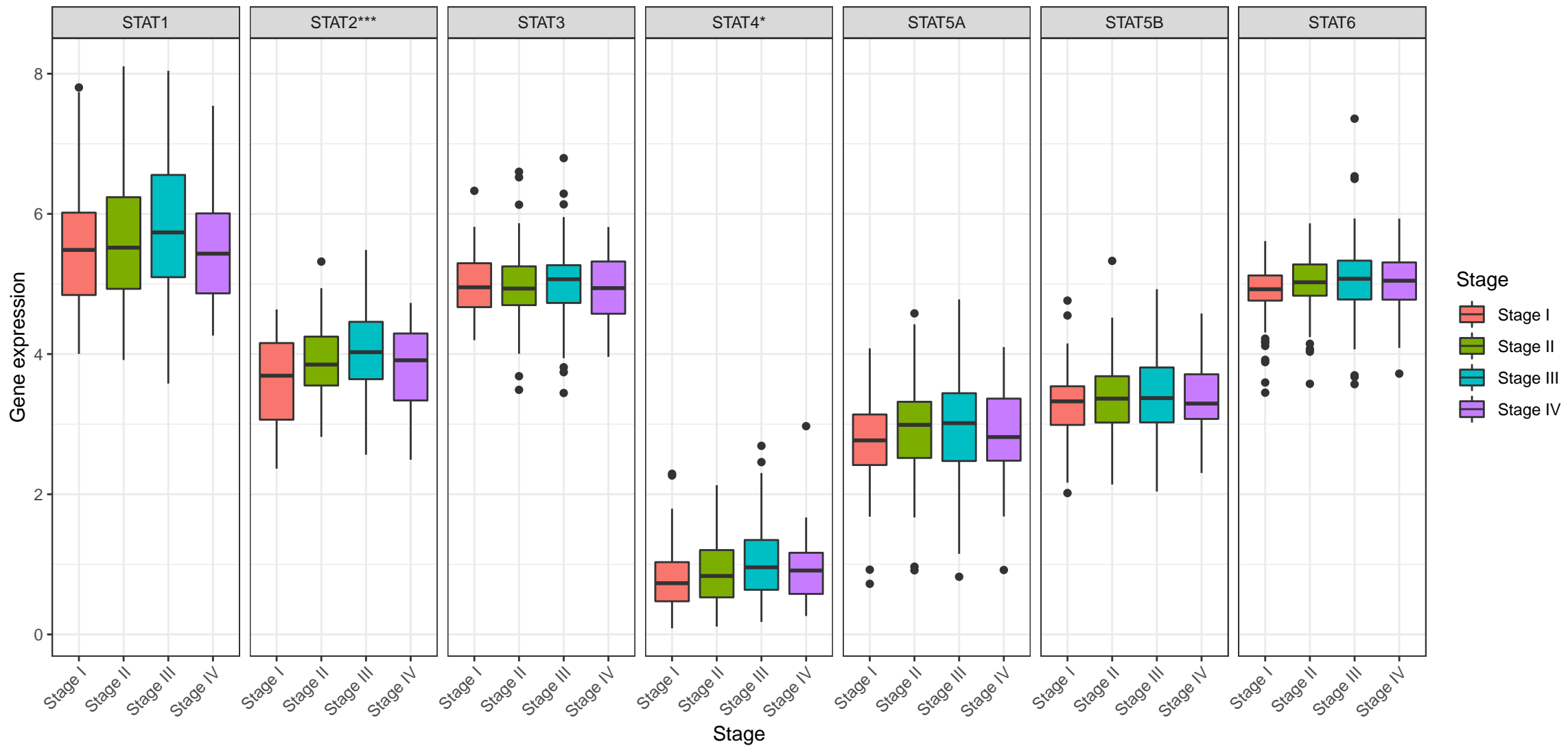

Supplement: Supplementary file 2 [file DataSheet1.ZIP › Source data/STAD cliCor.pdf]

Type 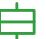 Normal 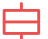 Tumor

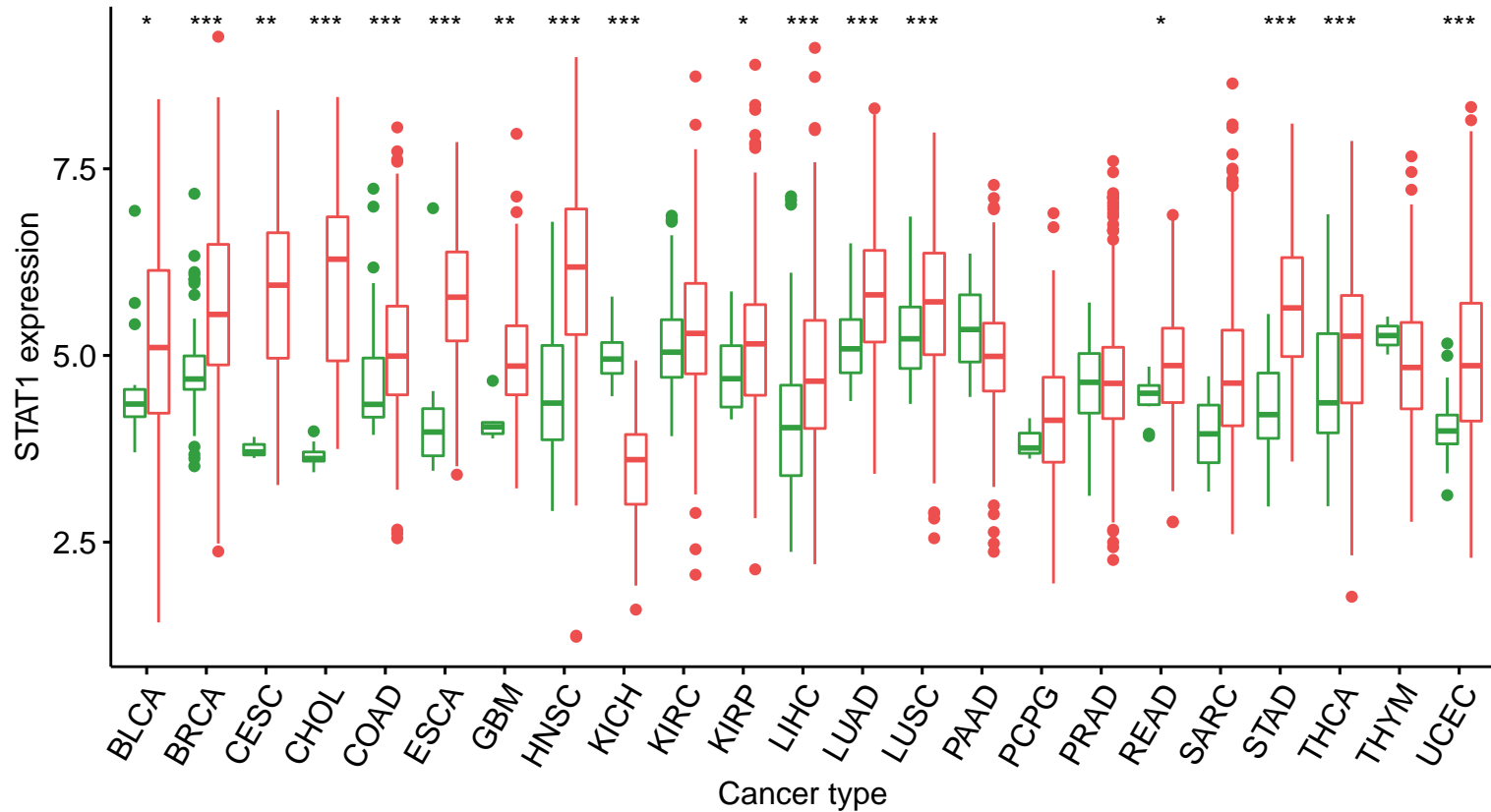

Supplement: Supplementary file 2 [file DataSheet1.ZIP › Source data/STAT1.diff.pdf]

# Cancer: ACC

STAT1 levels + high + low

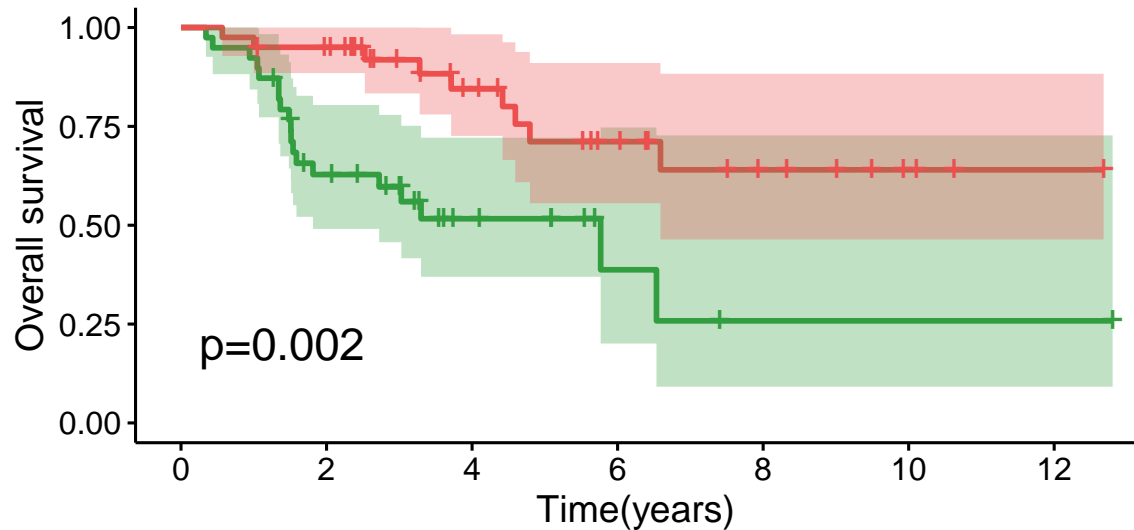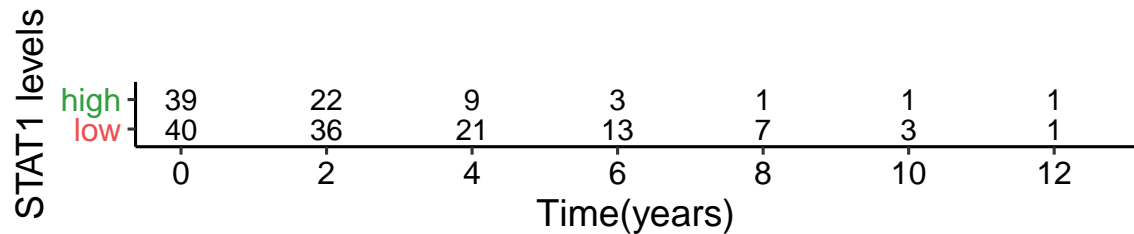

Supplement: Supplementary file 2 [file DataSheet1.ZIP › Source data/STAT1_ACC.pdf]

# Cancer: KIRP

STAT1 levels + high + low

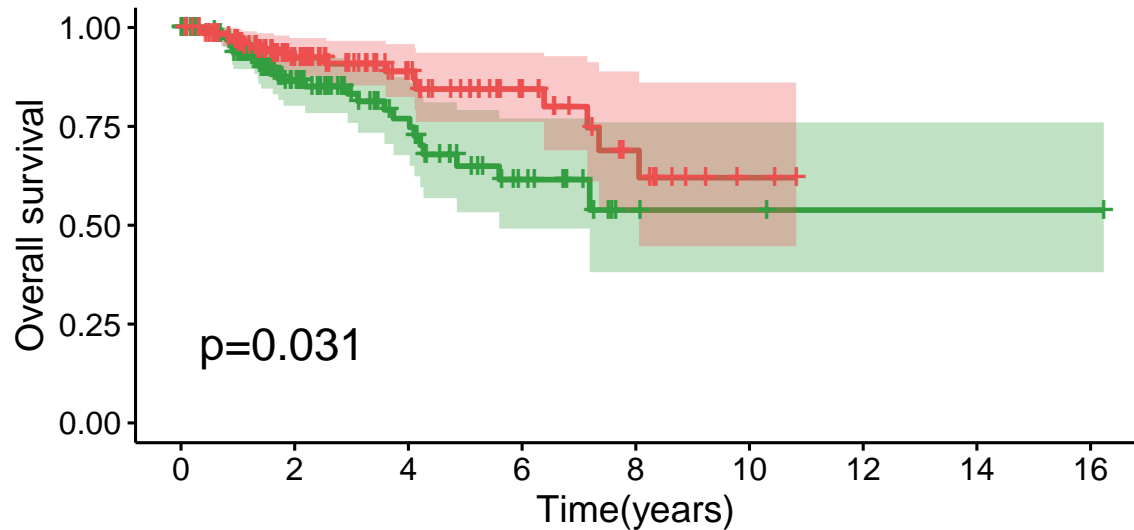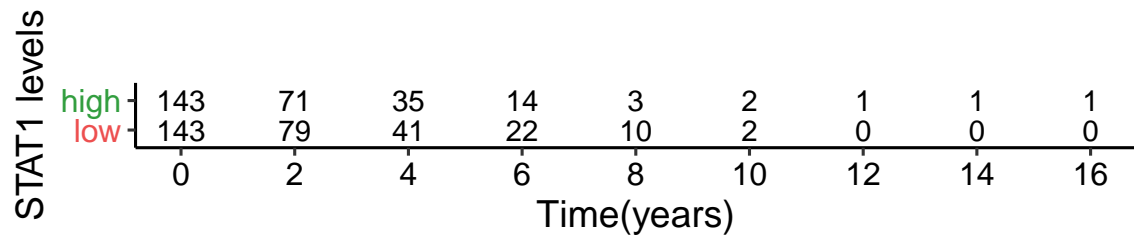

Supplement: Supplementary file 2 [file DataSheet1.ZIP › Source data/STAT1_KIRP.pdf]

# Cancer: LGG

STAT1 levels + high + low

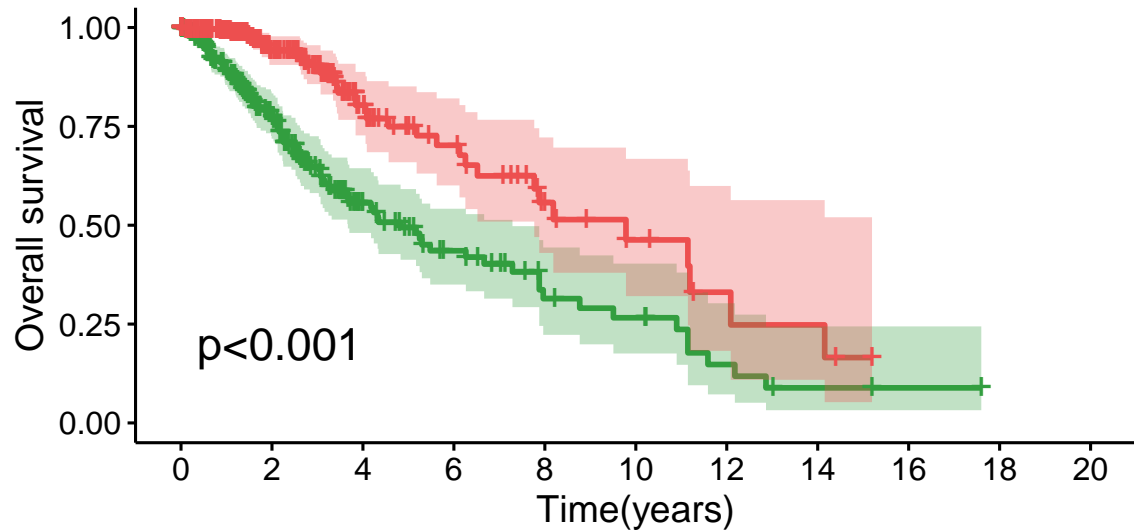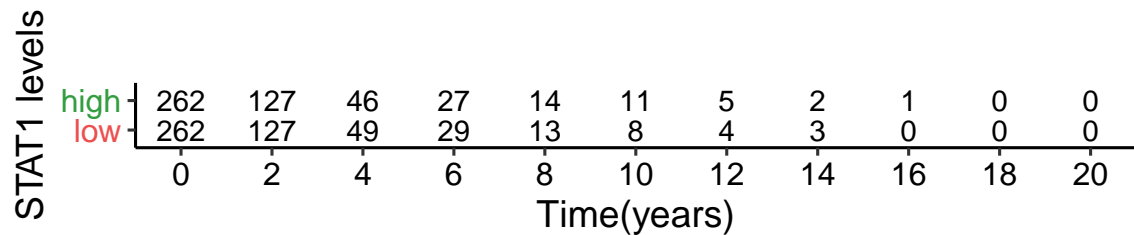

Supplement: Supplementary file 2 [file DataSheet1.ZIP › Source data/STAT1_LGG.pdf]

# Cancer: MESO

STAT1 levels + high + low

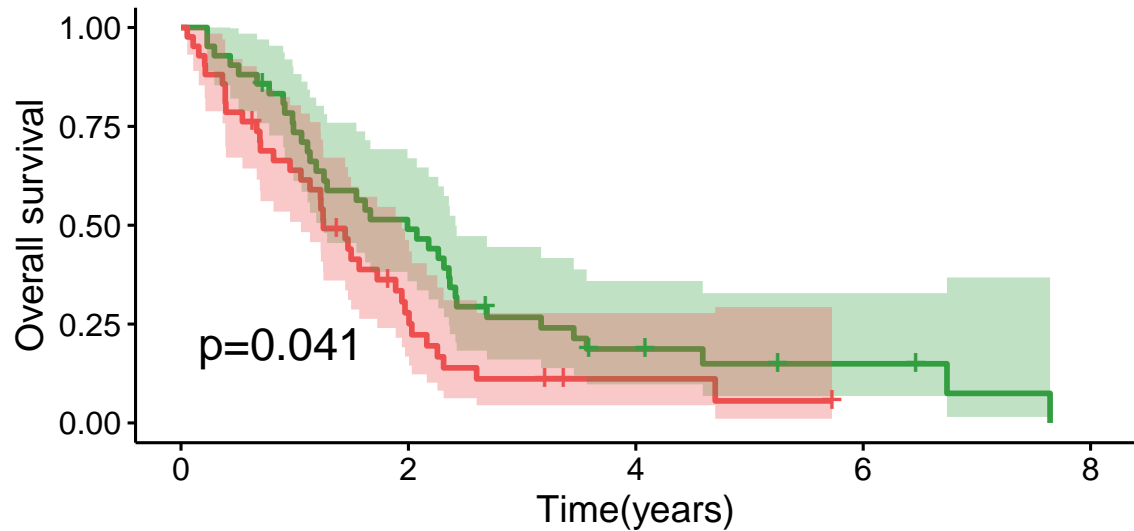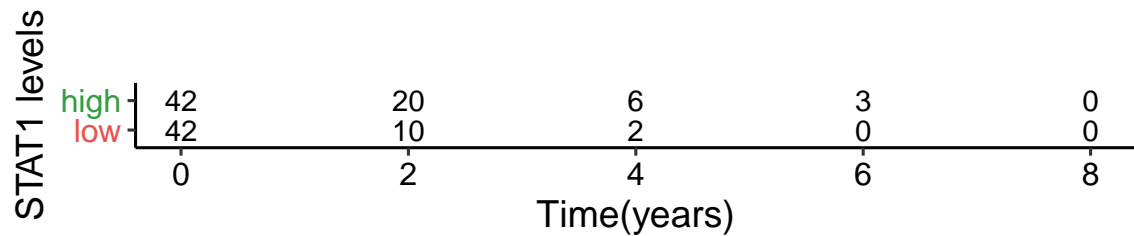

Supplement: Supplementary file 2 [file DataSheet1.ZIP › Source data/STAT1_MESO.pdf]

# Cancer: OV

STAT1 levels + high + low

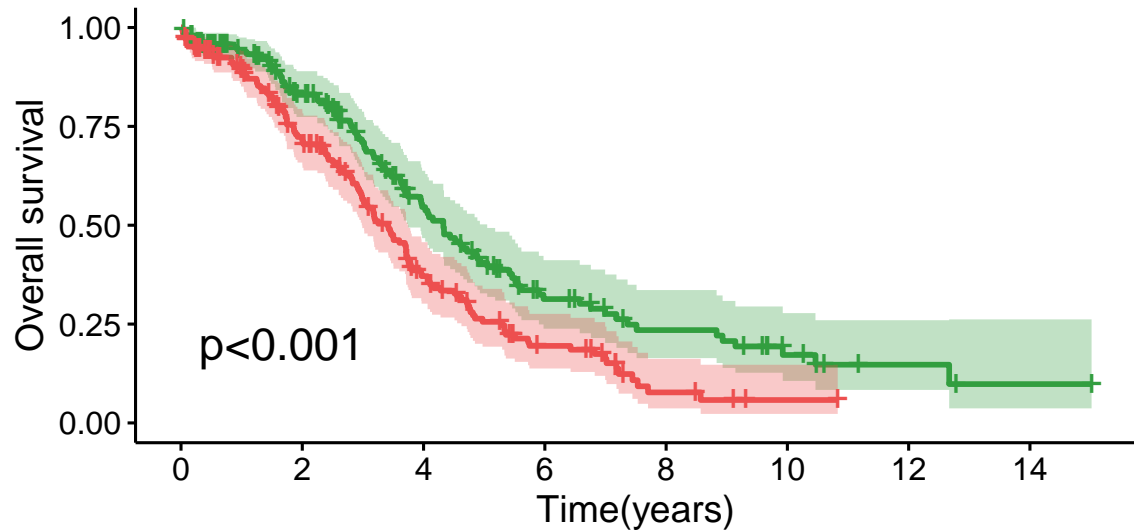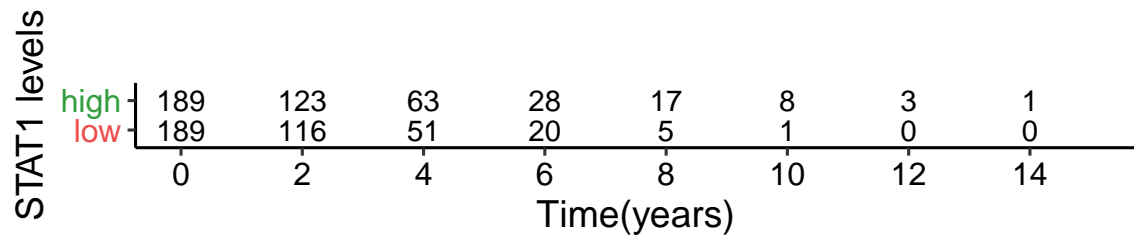

Supplement: Supplementary file 2 [file DataSheet1.ZIP › Source data/STAT1_OV.pdf]

# Cancer: PAAD

STAT1 levels + high + low

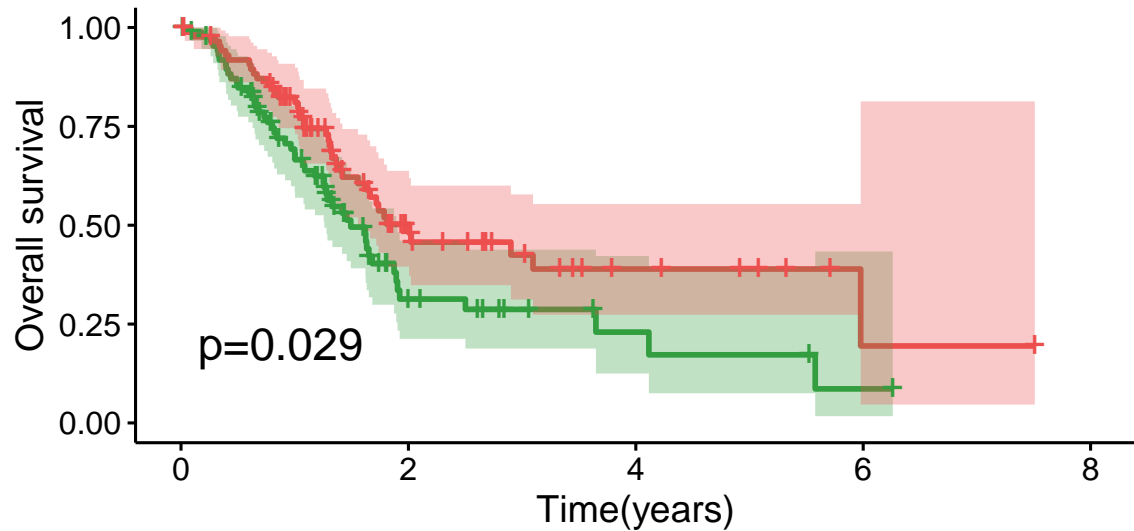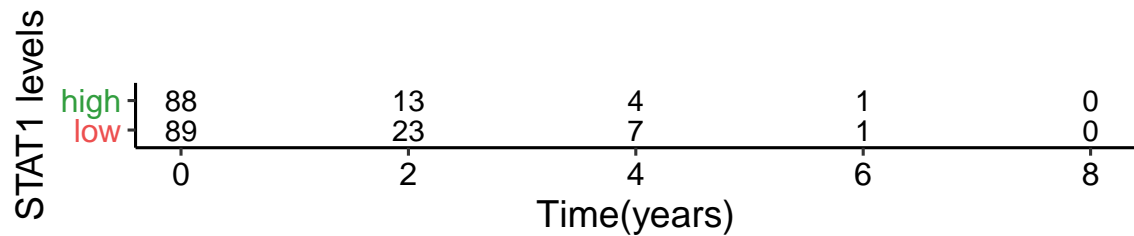

Supplement: Supplementary file 2 [file DataSheet1.ZIP › Source data/STAT1_PAAD.pdf]

# Cancer: READ

STAT1 levels + high + low

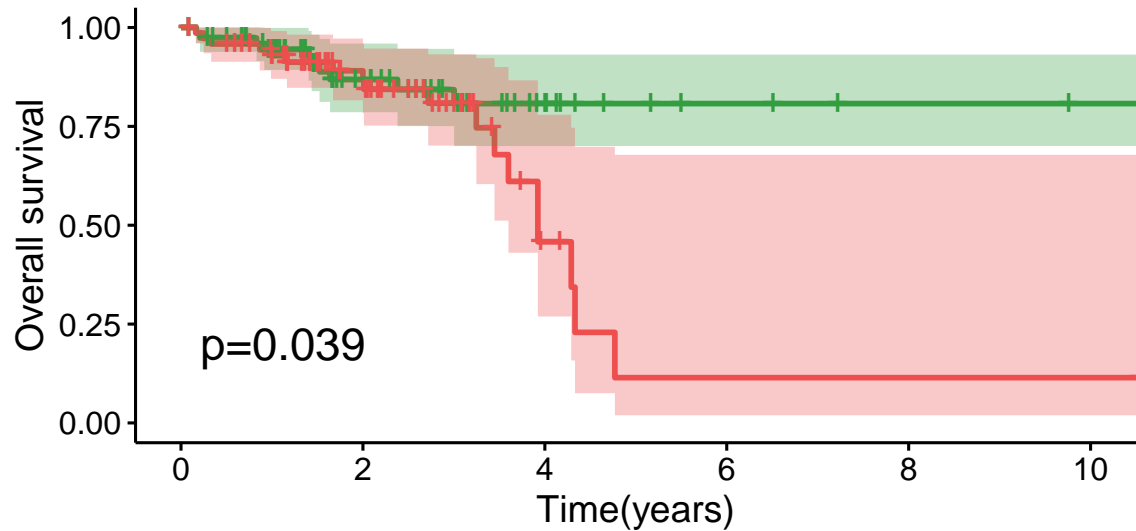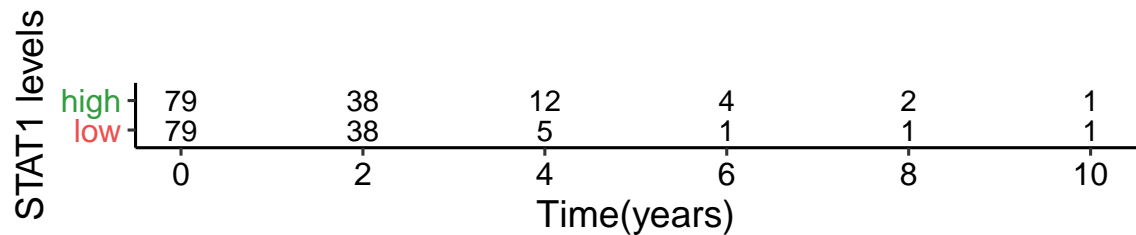

Supplement: Supplementary file 2 [file DataSheet1.ZIP › Source data/STAT1_READ.pdf]

# Cancer: SKCM

STAT1 levels + high + low

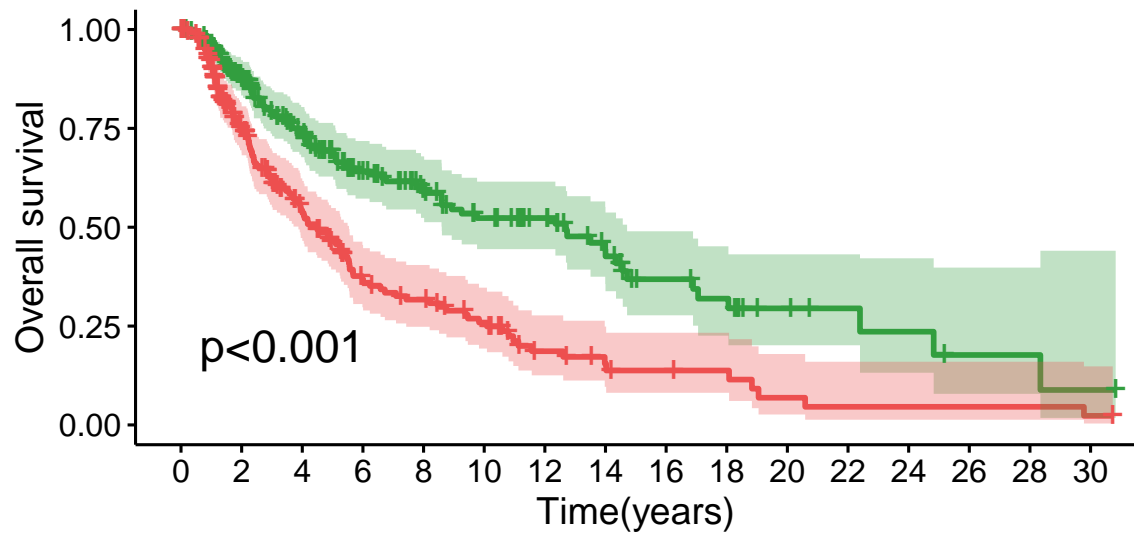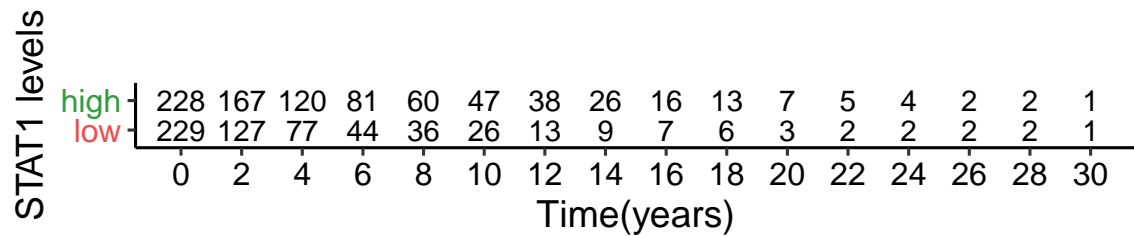

Supplement: Supplementary file 2 [file DataSheet1.ZIP › Source data/STAT1_SKCM.pdf]

Type 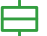 Normal 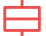 Tumor

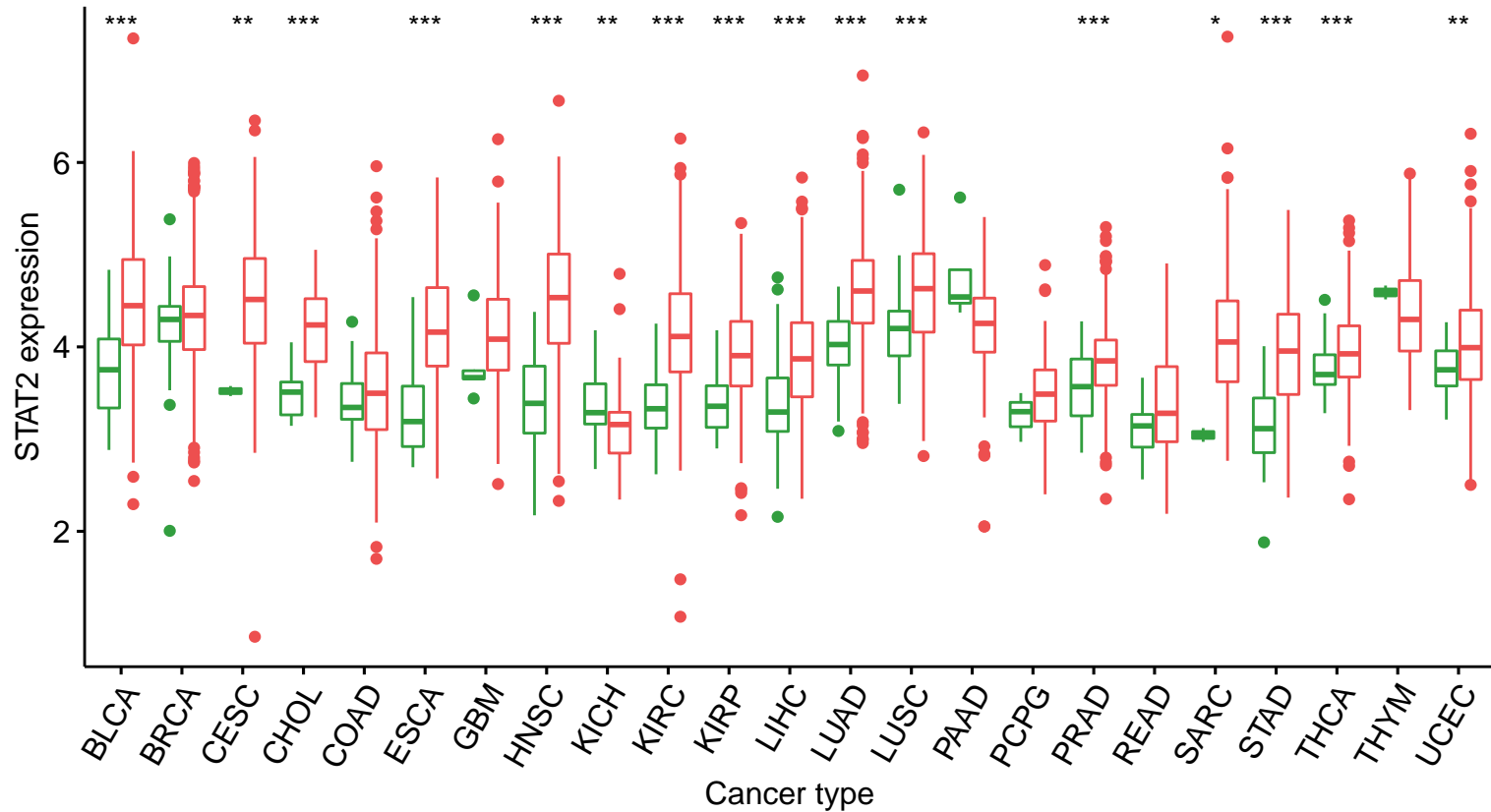

Supplement: Supplementary file 2 [file DataSheet1.ZIP › Source data/STAT2.diff.pdf]

# Cancer: KIRC

STAT2 levels + high + low

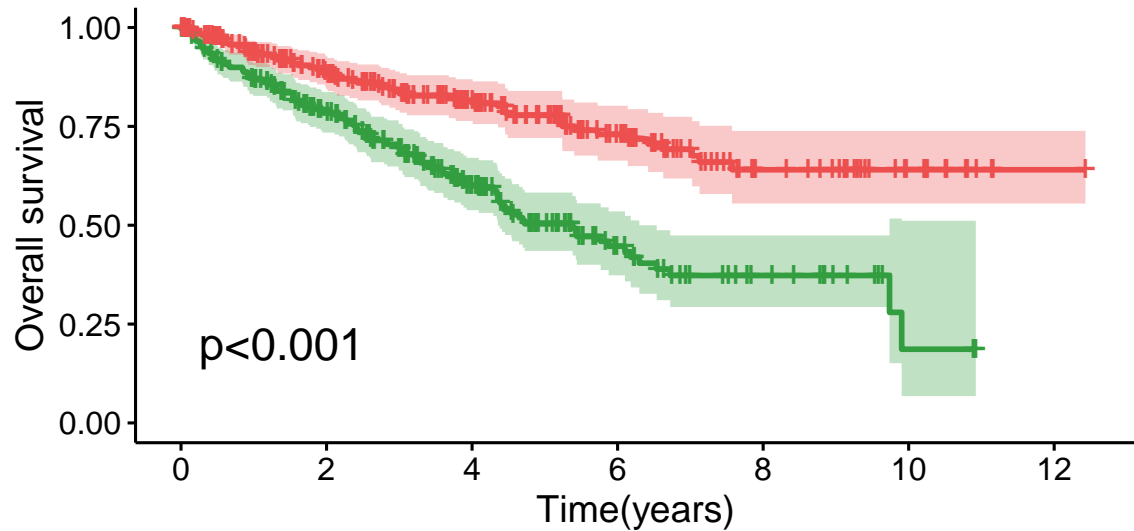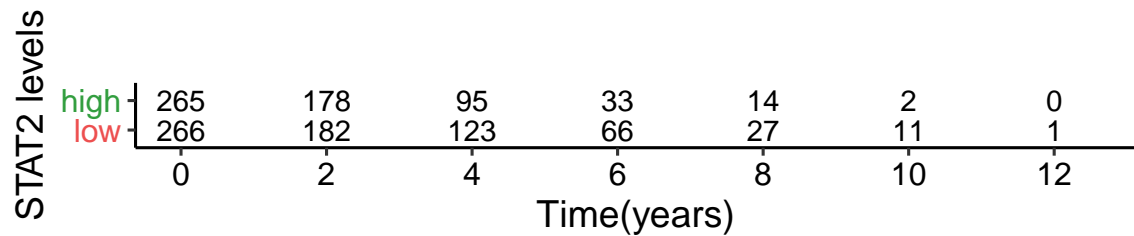

Supplement: Supplementary file 2 [file DataSheet1.ZIP › Source data/STAT2_KIRC.pdf]

# Cancer: LGG

STAT2 levels + high + low

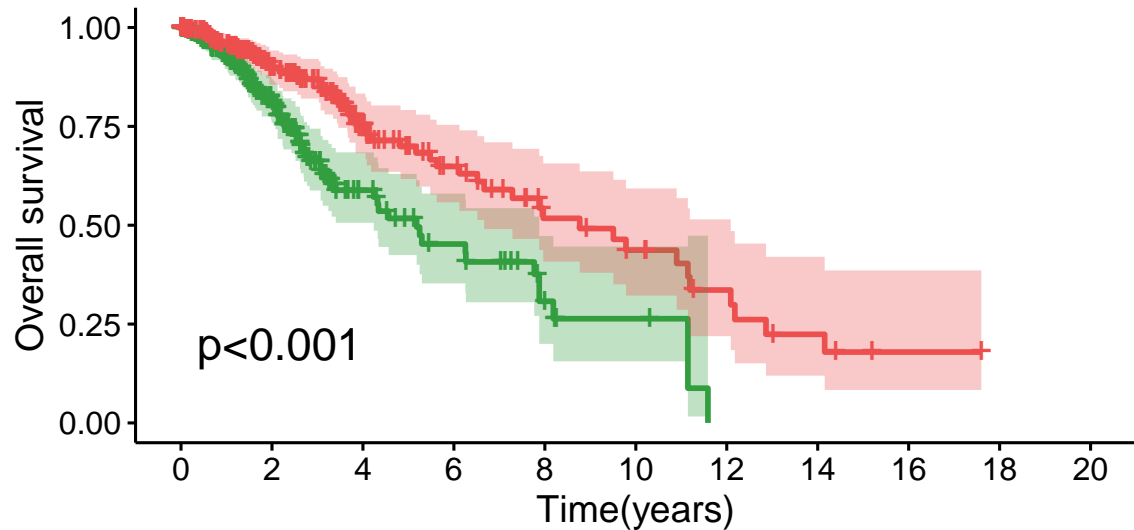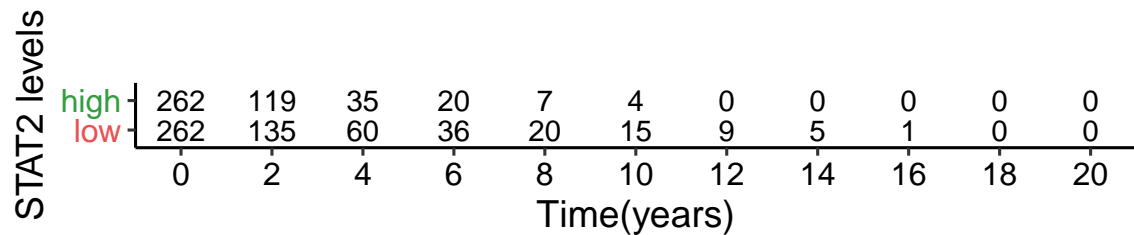

Supplement: Supplementary file 2 [file DataSheet1.ZIP › Source data/STAT2_LGG.pdf]

Type 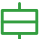 Normal 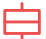 Tumor

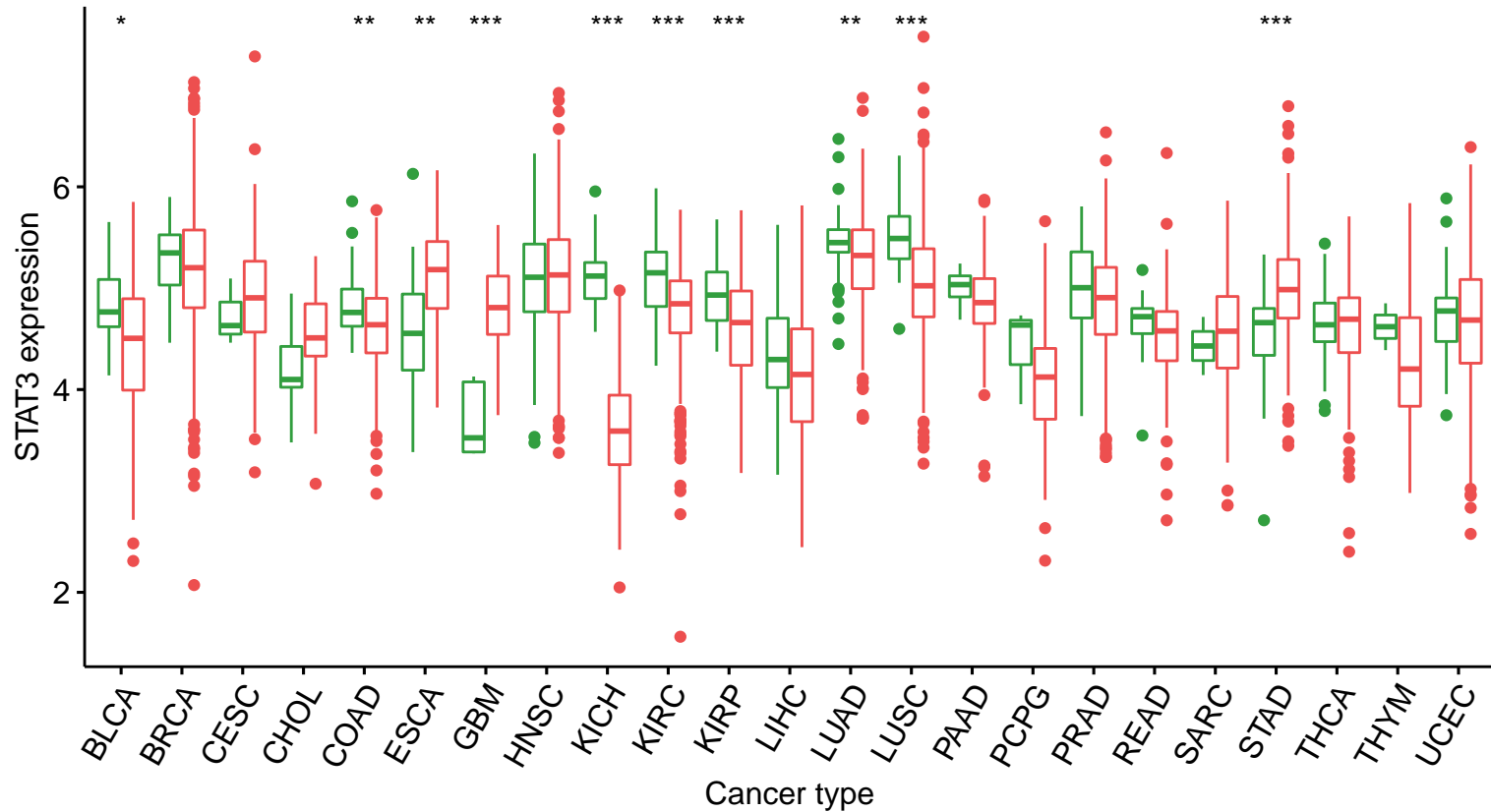

Supplement: Supplementary file 2 [file DataSheet1.ZIP › Source data/STAT3.diff.pdf]

# Cancer: LGG

STAT3 levels + high + low

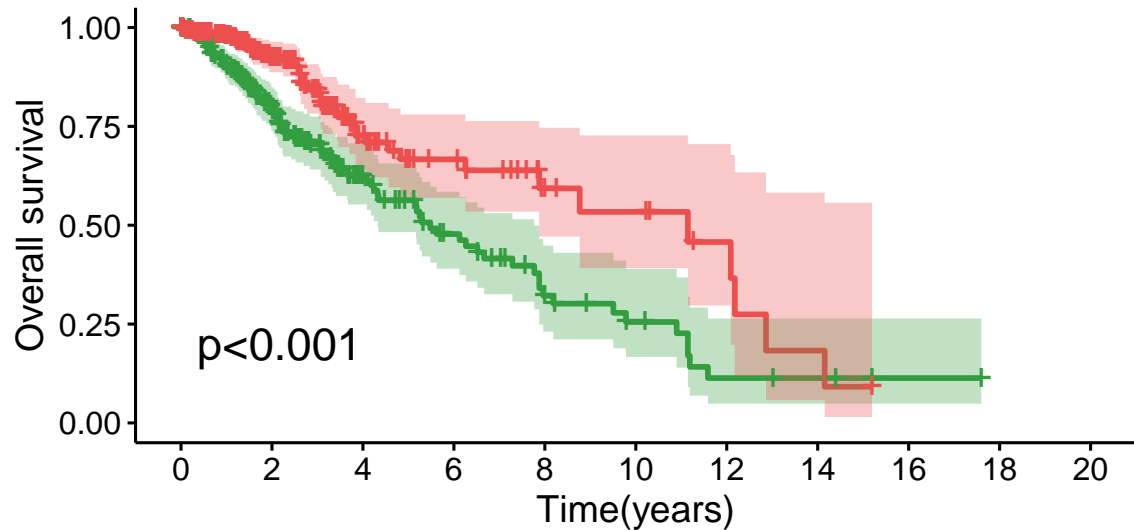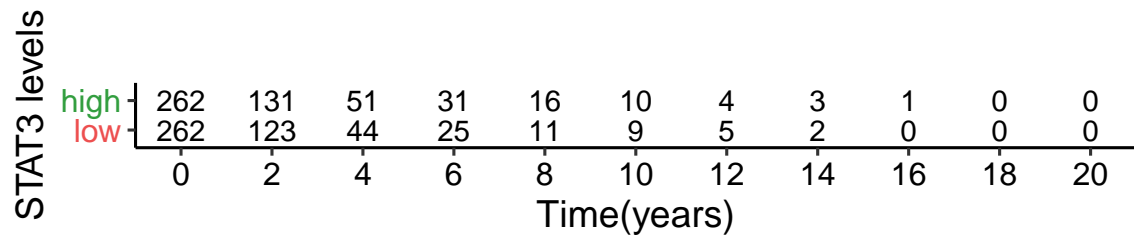

Supplement: Supplementary file 2 [file DataSheet1.ZIP › Source data/STAT3_LGG.pdf]

# Cancer: SKCM

STAT3 levels + high + low

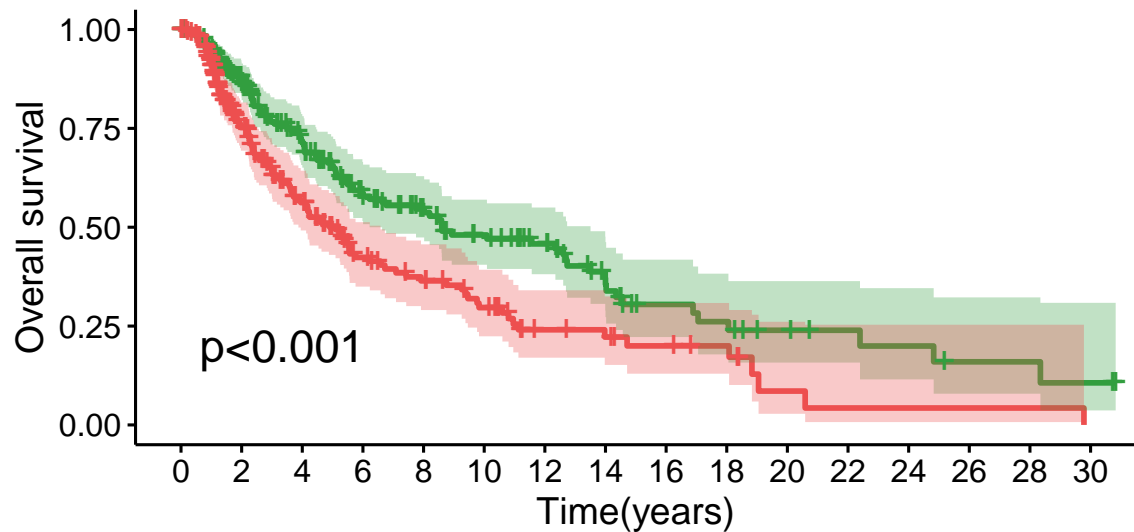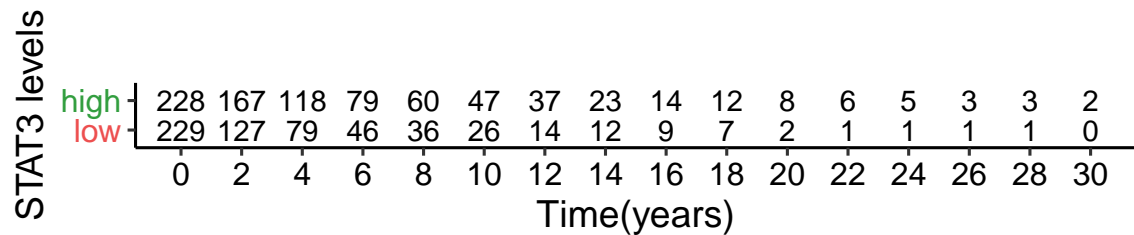

Supplement: Supplementary file 2 [file DataSheet1.ZIP › Source data/STAT3_SKCM.pdf]

# Cancer: TGCT

STAT3 levels + high + low

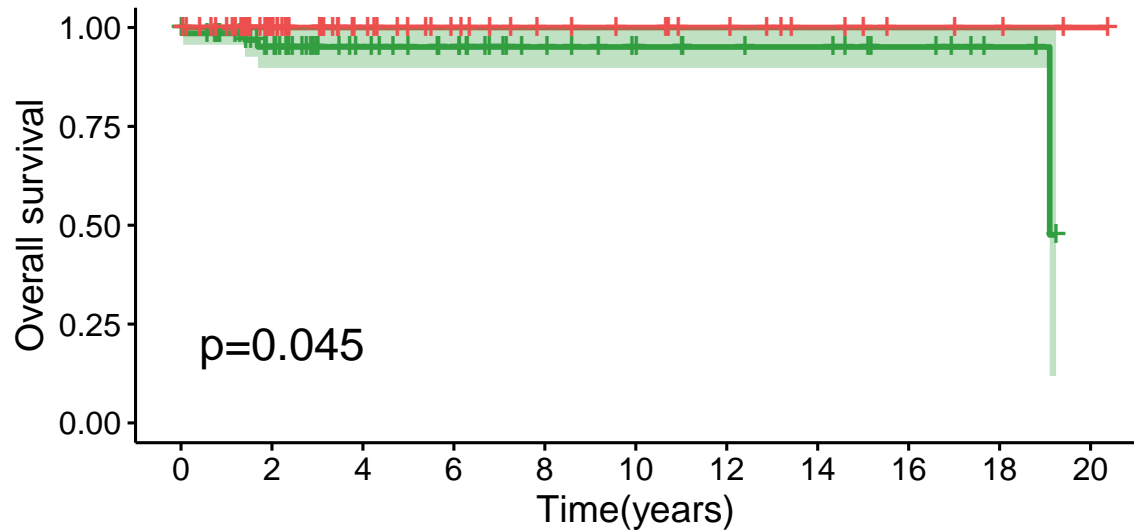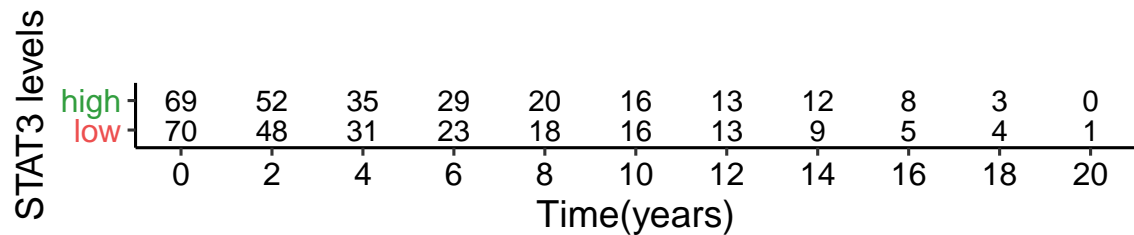

Supplement: Supplementary file 2 [file DataSheet1.ZIP › Source data/STAT3_TGCT.pdf]

Type 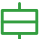 Normal 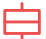 Tumor

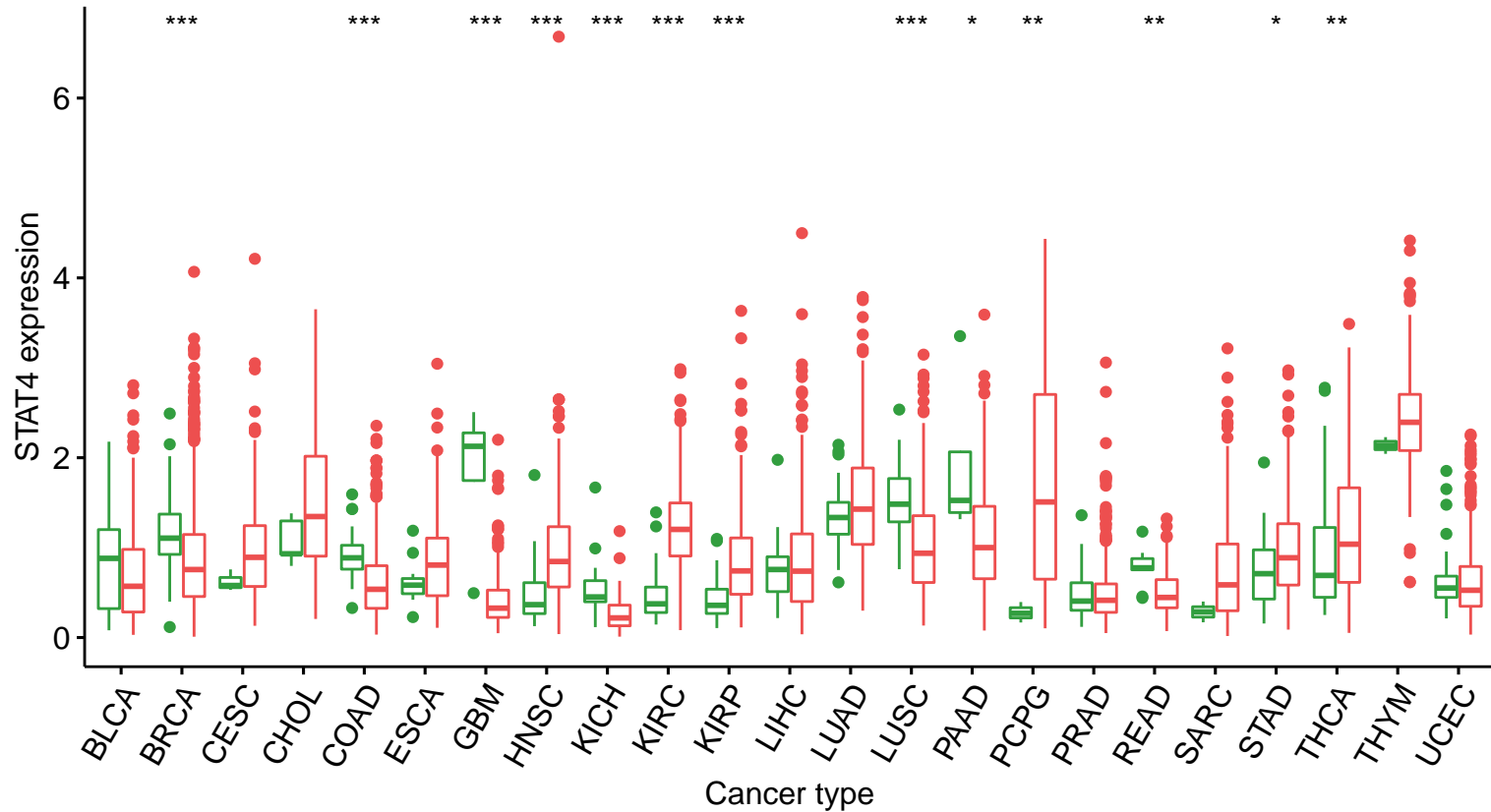

Supplement: Supplementary file 2 [file DataSheet1.ZIP › Source data/STAT4.diff.pdf]

# Cancer: BRCA

STAT4 levels + high + low

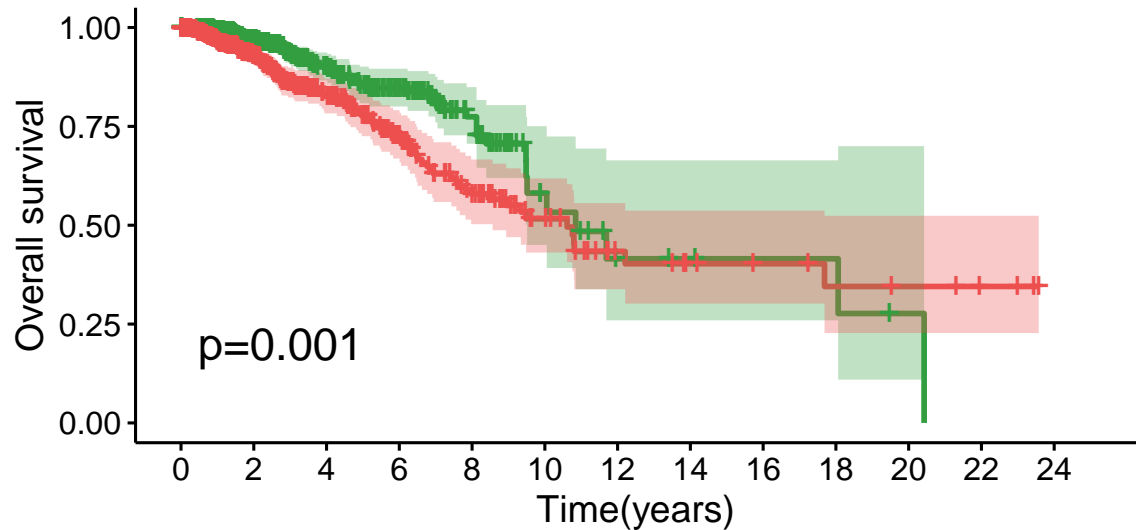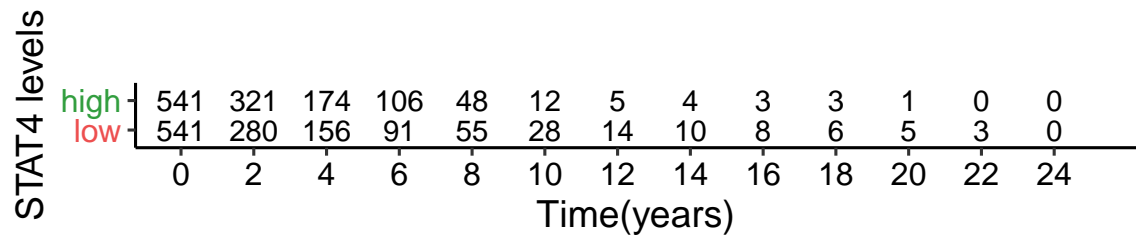

Supplement: Supplementary file 2 [file DataSheet1.ZIP › Source data/STAT4_BRCA.pdf]

# Cancer: KIRC

STAT4 levels + high + low

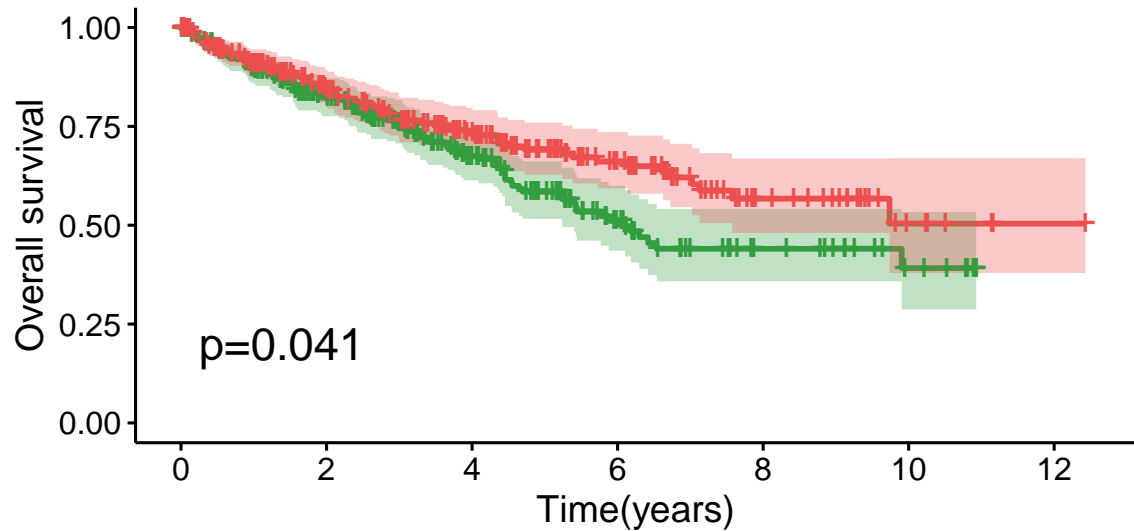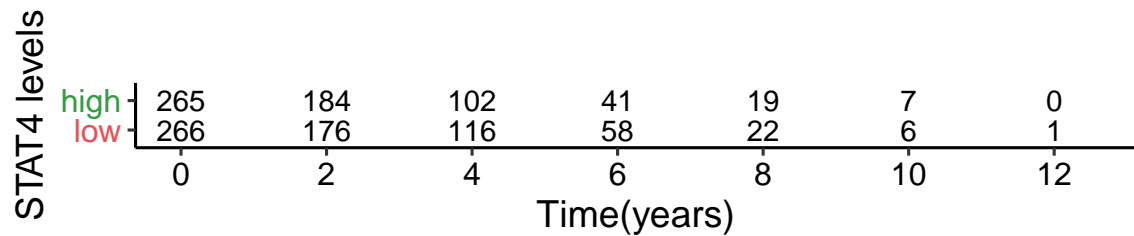

Supplement: Supplementary file 2 [file DataSheet1.ZIP › Source data/STAT4_KIRC.pdf]

# Cancer: KIRP

STAT4 levels + high + low

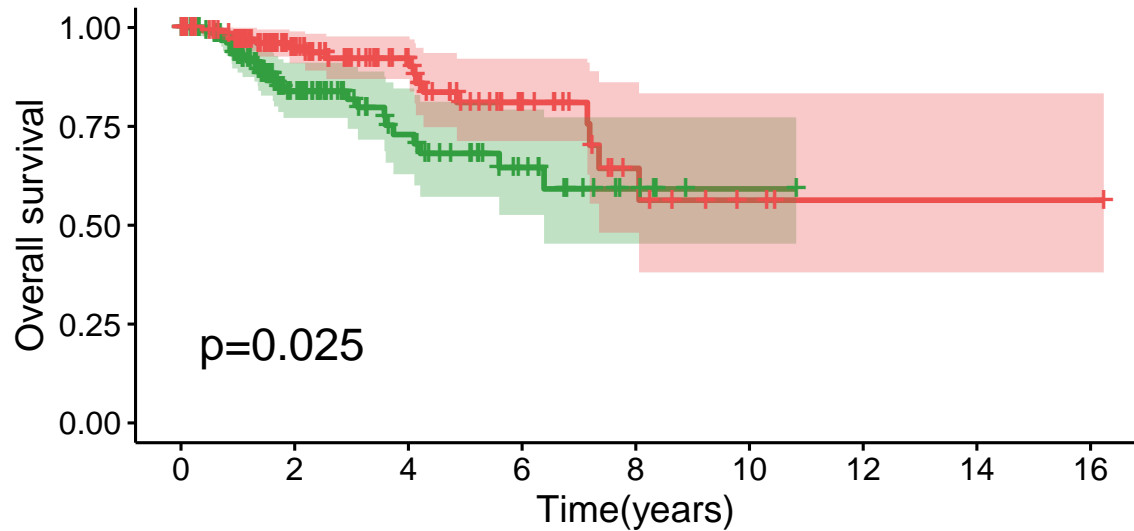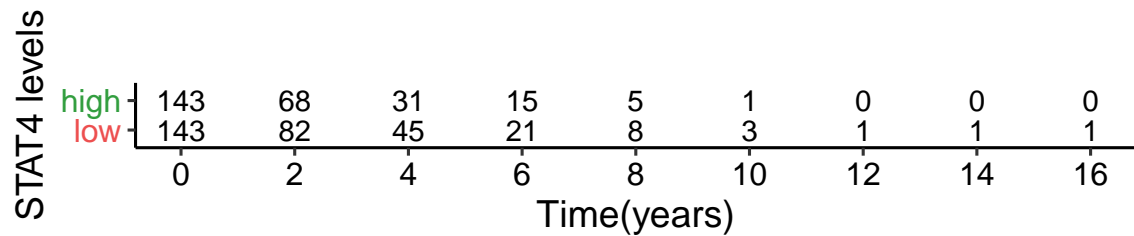

Supplement: Supplementary file 2 [file DataSheet1.ZIP › Source data/STAT4_KIRP.pdf]

# Cancer: OV

STAT4 levels + high + low

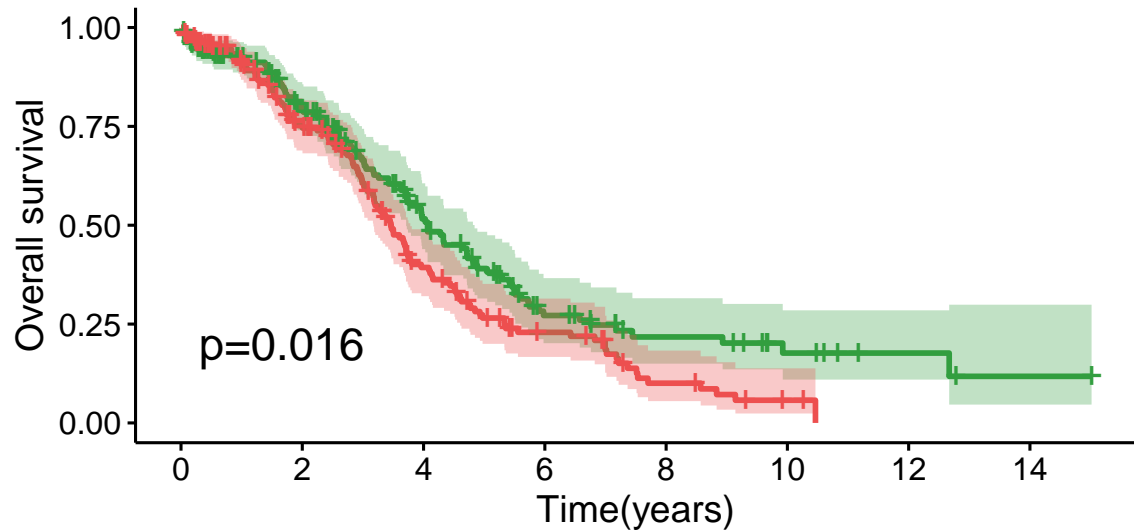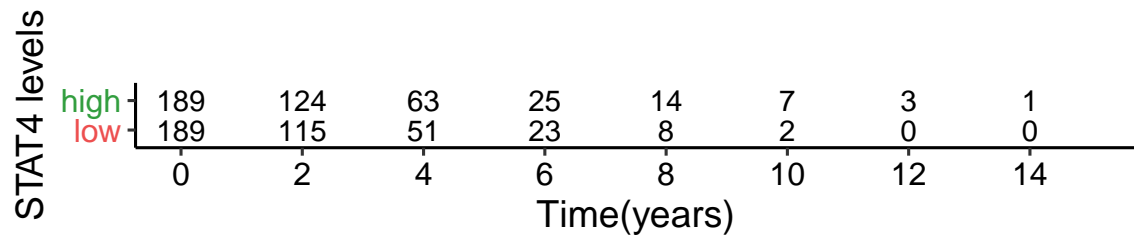

Supplement: Supplementary file 2 [file DataSheet1.ZIP › Source data/STAT4_OV.pdf]

# Cancer: PAAD

STAT4 levels + high + low

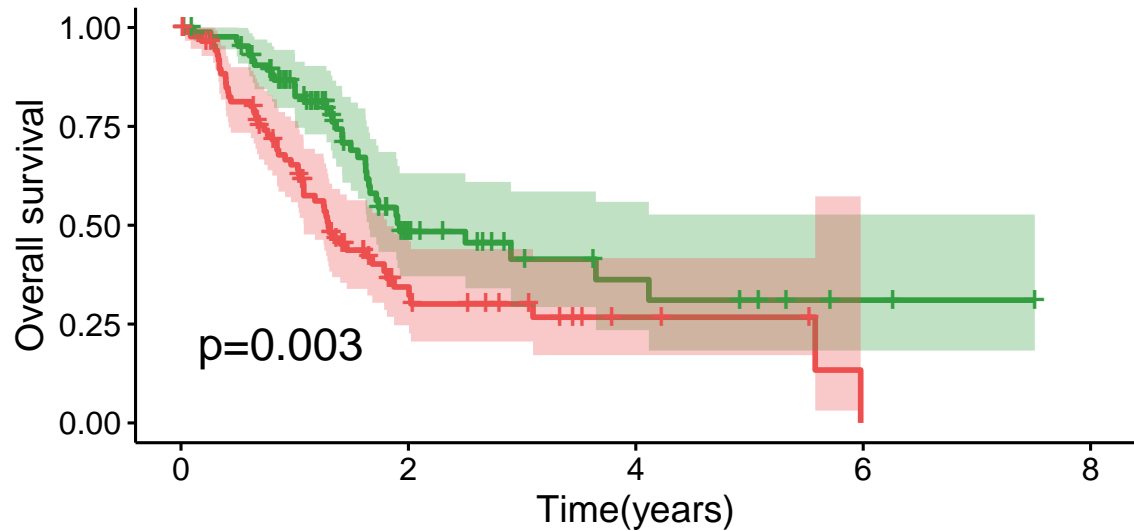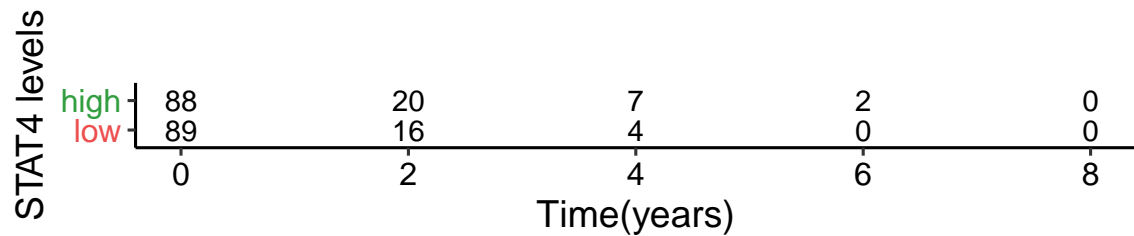

Supplement: Supplementary file 2 [file DataSheet1.ZIP › Source data/STAT4_PAAD.pdf]

# Cancer: SKCM

STAT4 levels + high + low

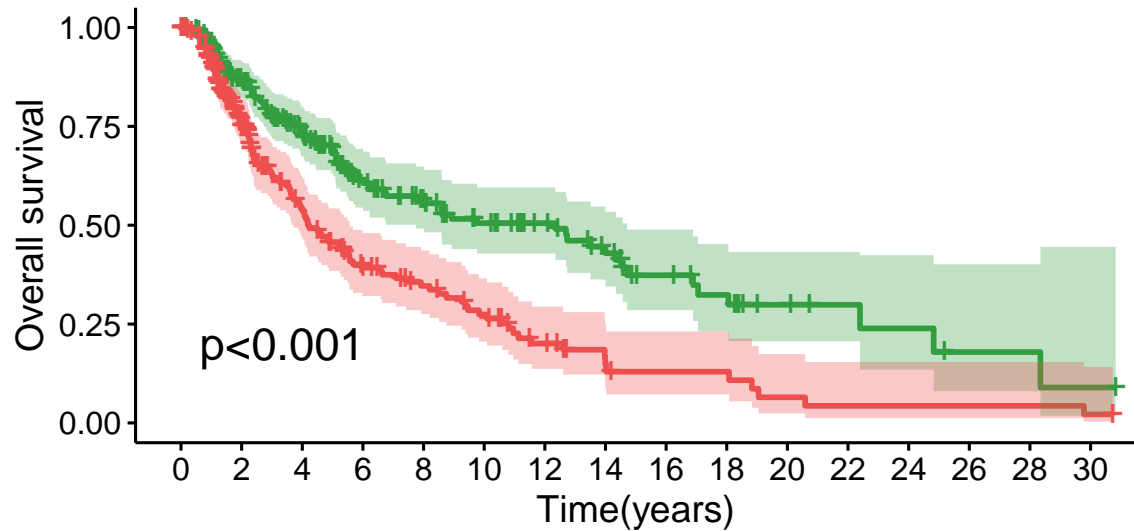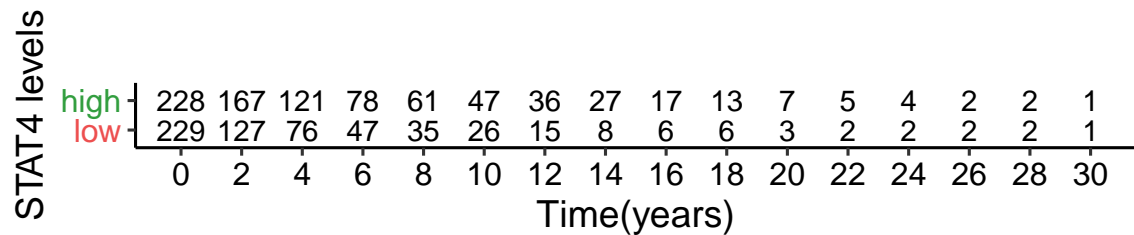

Supplement: Supplementary file 2 [file DataSheet1.ZIP › Source data/STAT4_SKCM.pdf]

Type 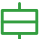 Normal 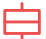 Tumor

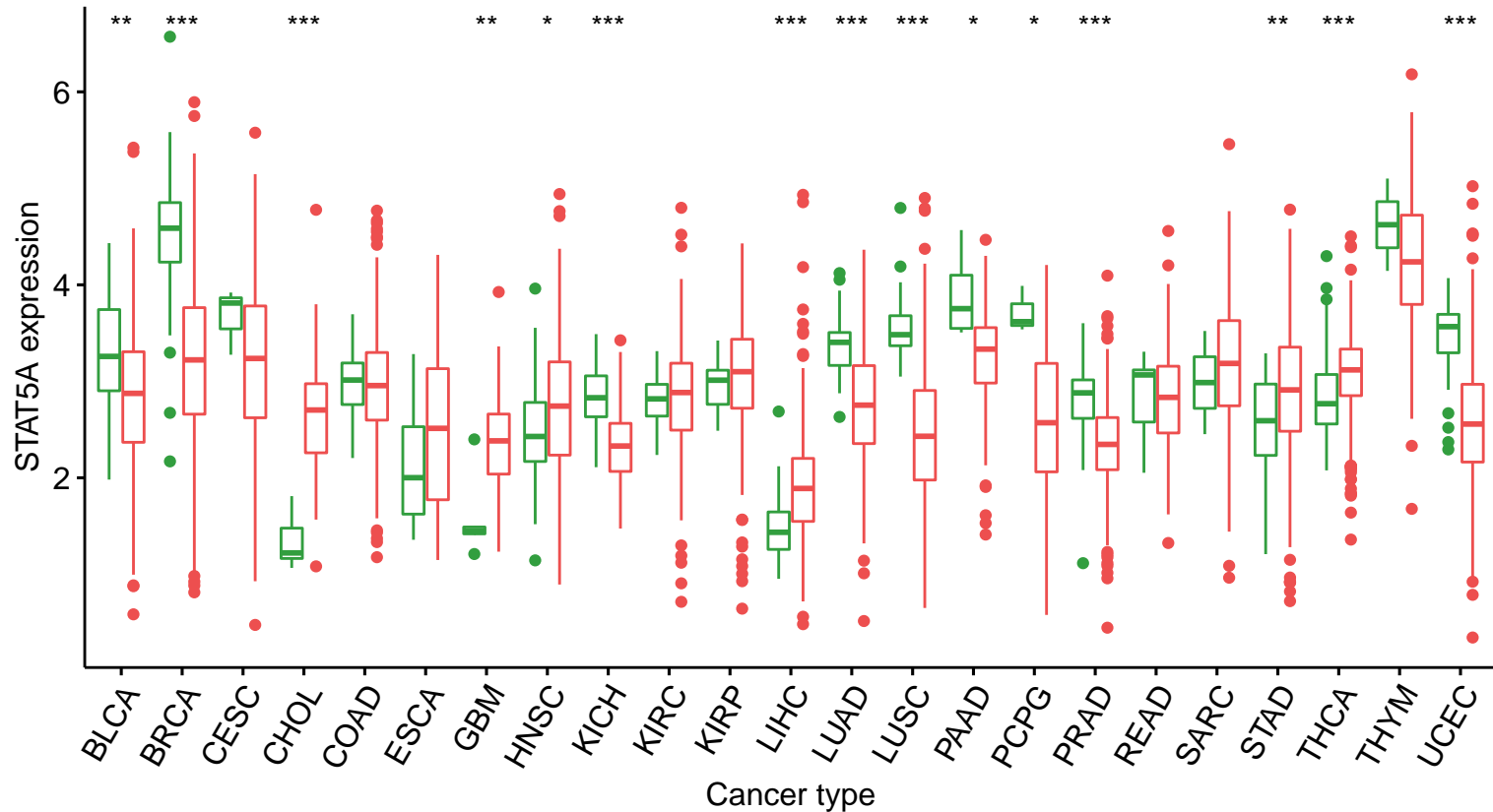

Supplement: Supplementary file 2 [file DataSheet1.ZIP › Source data/STAT5A.diff.pdf]

# Cancer: HNSC

STAT5A levels + high + low

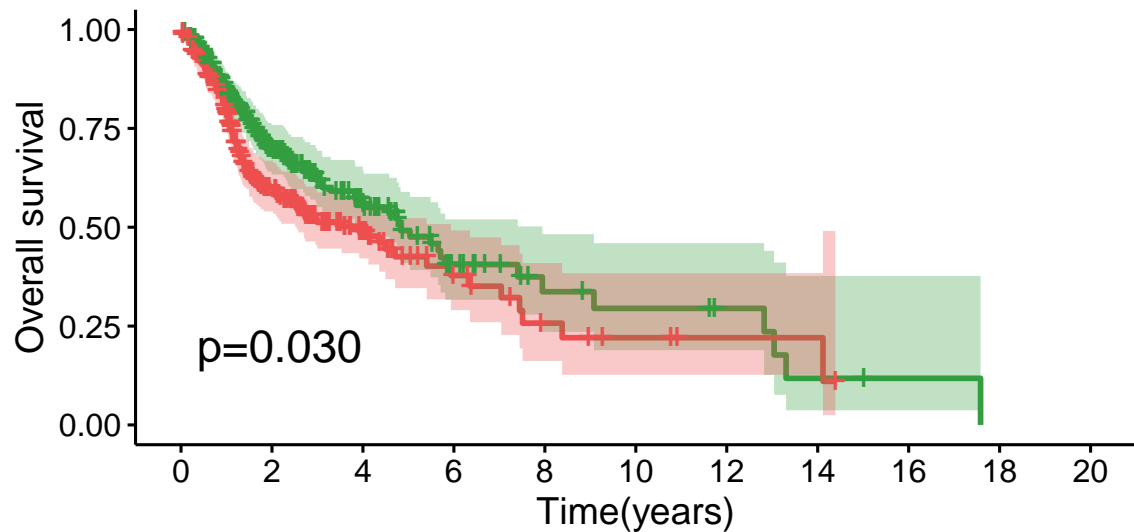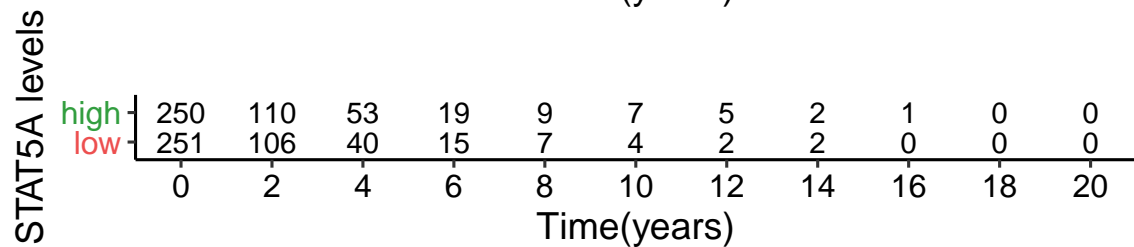

Supplement: Supplementary file 2 [file DataSheet1.ZIP › Source data/STAT5A_HNSC.pdf]

# Cancer: LGG

STAT5A levels + high + low

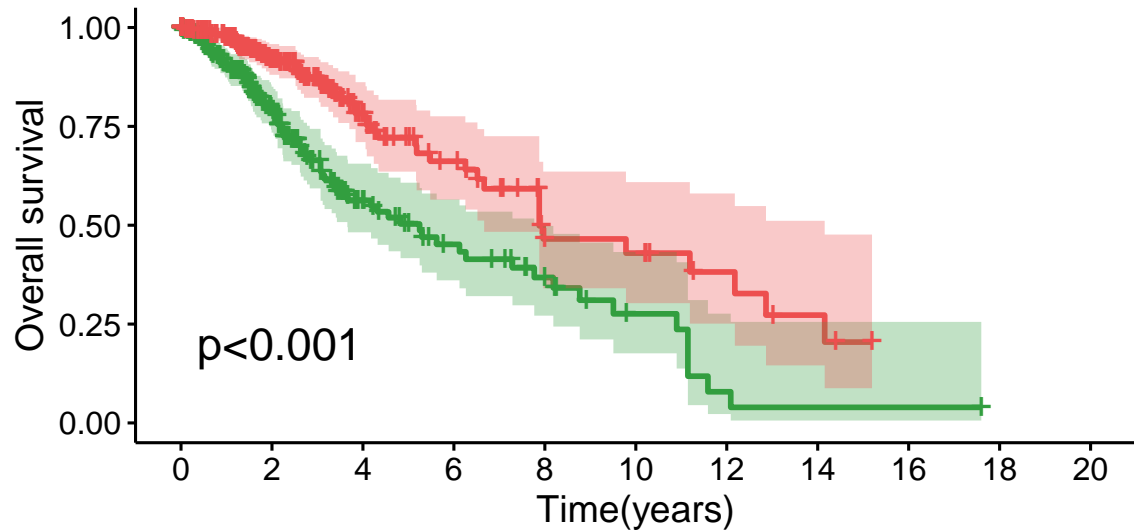

STAT5A levels

high

low

|     |     |    |    |    |    |    |    |    |    |    |
|-----|-----|----|----|----|----|----|----|----|----|----|
| 262 | 115 | 41 | 24 | 14 | 7  | 2  | 1  | 1  | 0  | 0  |
| 262 | 139 | 54 | 32 | 13 | 12 | 7  | 4  | 0  | 0  | 0  |
| 0   | 2   | 4  | 6  | 8  | 10 | 12 | 14 | 16 | 18 | 20 |

Time(years)

Supplement: Supplementary file 2 [file DataSheet1.ZIP › Source data/STAT5A_LGG.pdf]

# Cancer: MESO

STAT5A levels + high + low

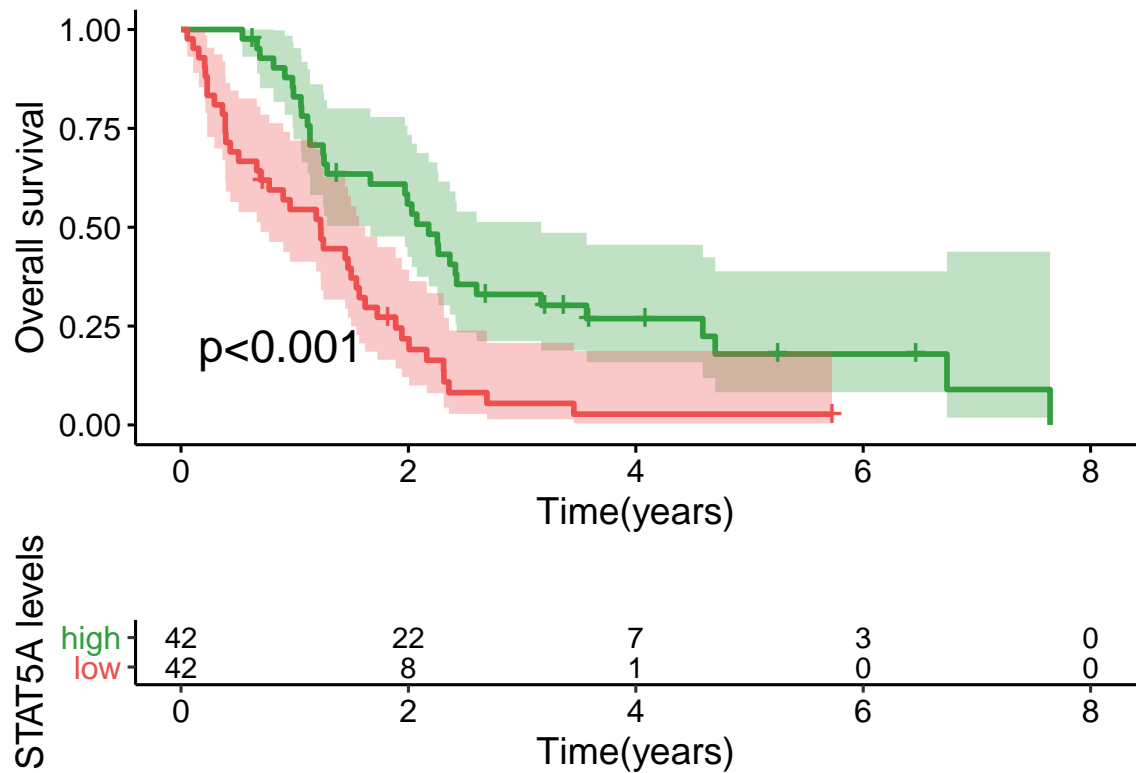

Supplement: Supplementary file 2 [file DataSheet1.ZIP › Source data/STAT5A_MESO.pdf]

# Cancer: SKCM

STAT5A levels + high + low

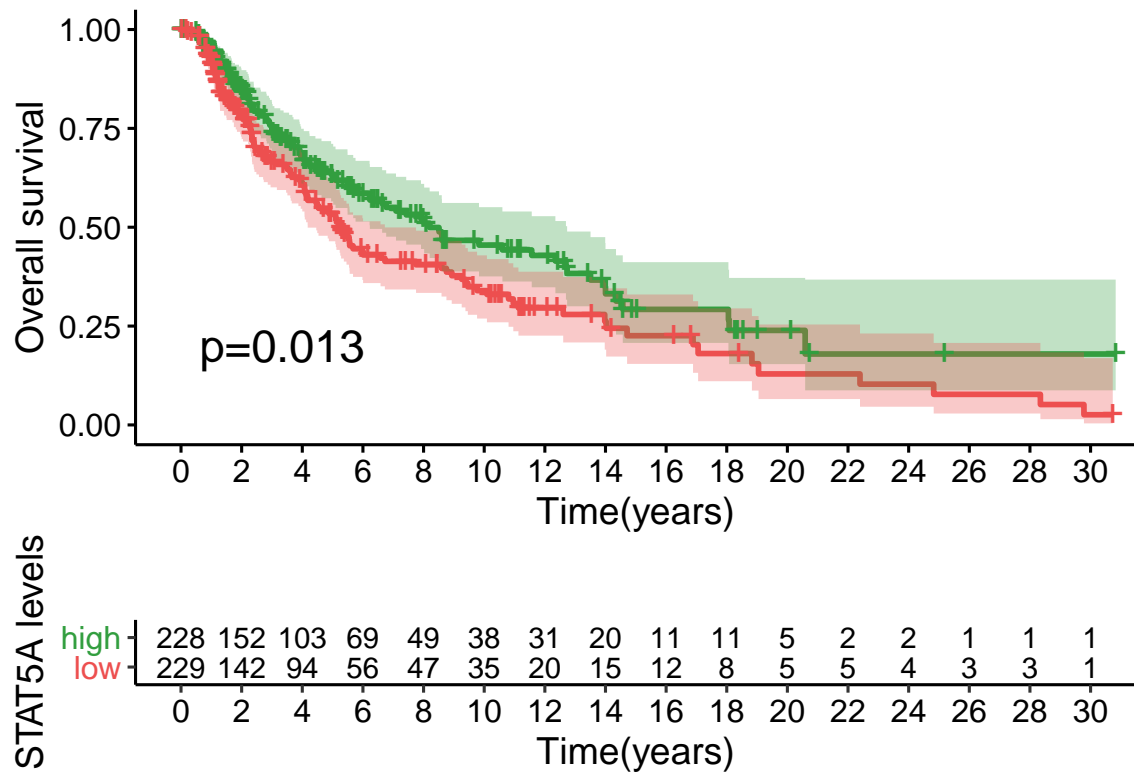

Supplement: Supplementary file 2 [file DataSheet1.ZIP › Source data/STAT5A_SKCM.pdf]

Type 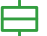 Normal 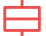 Tumor

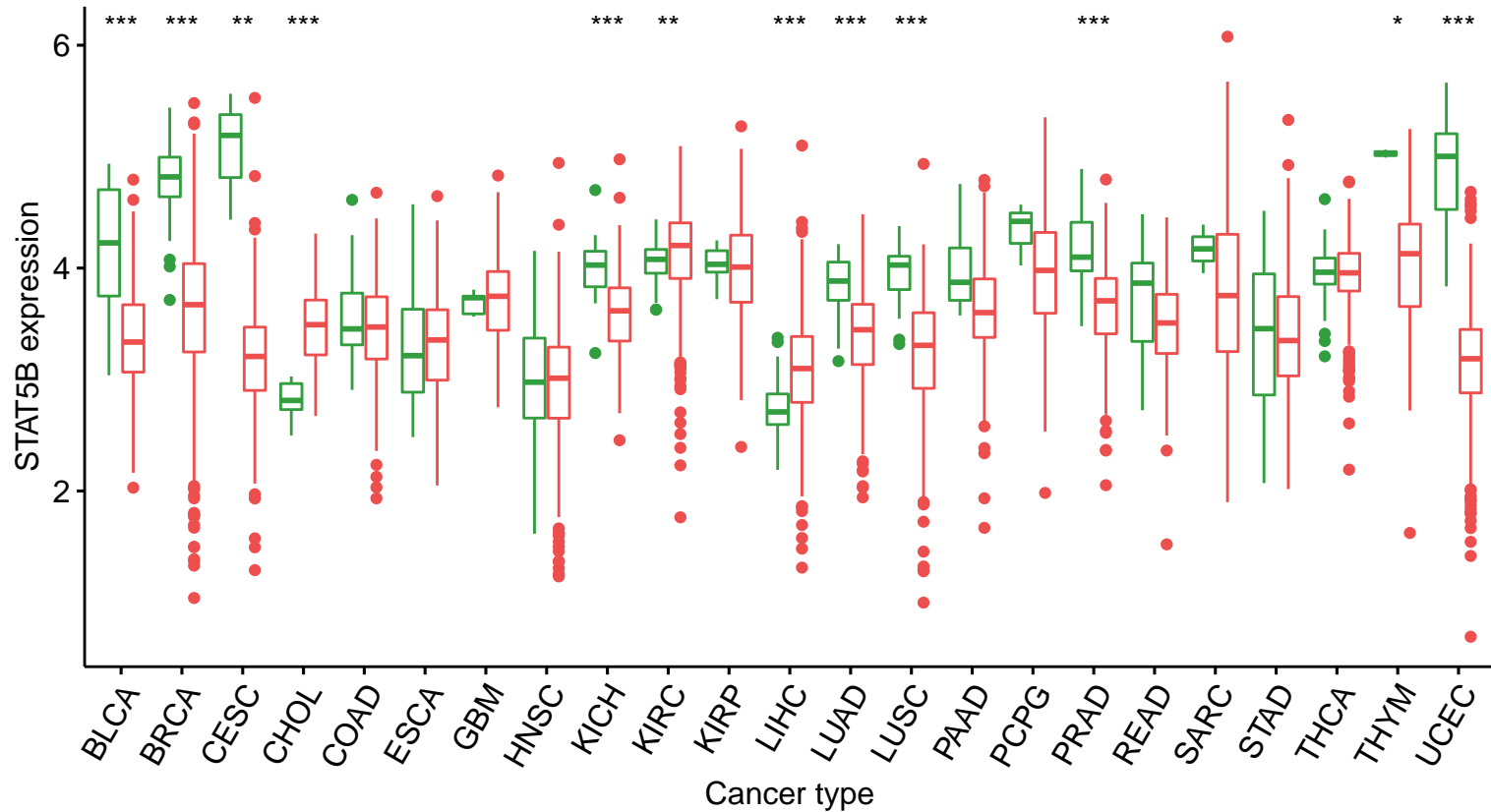

Supplement: Supplementary file 2 [file DataSheet1.ZIP › Source data/STAT5B.diff.pdf]

# Cancer: ACC

STAT5B levels + high + low

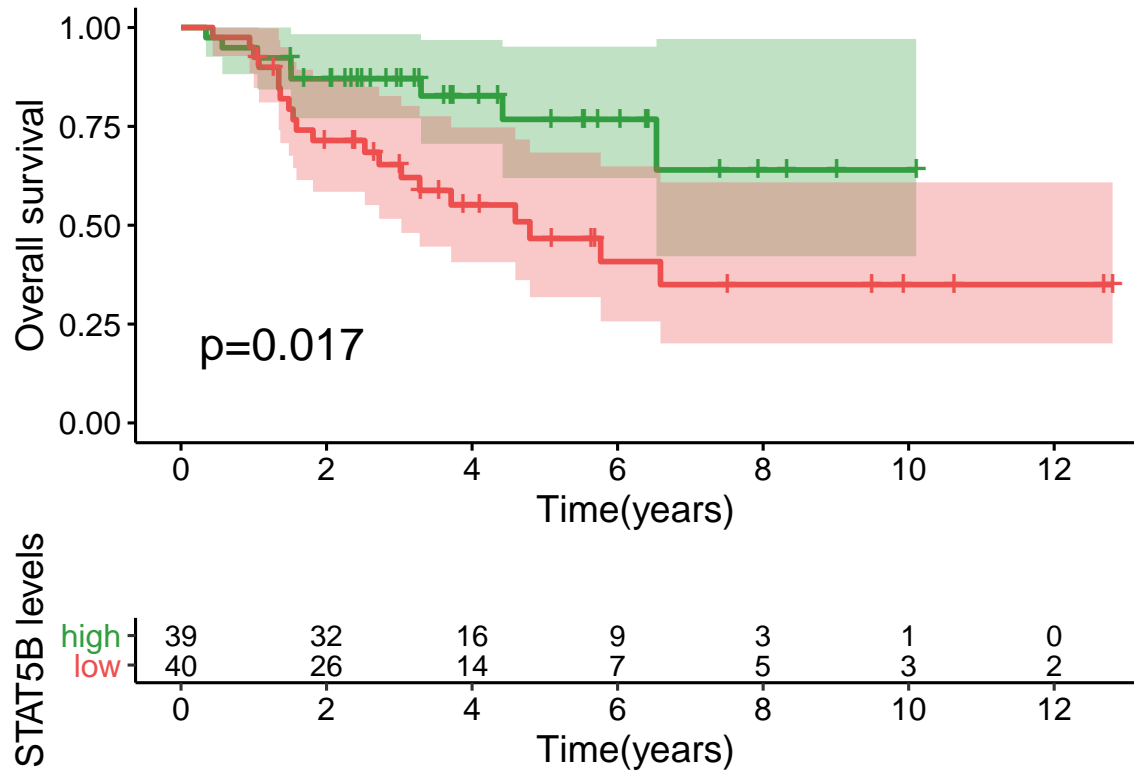

Supplement: Supplementary file 2 [file DataSheet1.ZIP › Source data/STAT5B_ACC.pdf]

# Cancer: KIRC

STAT5B levels + high + low

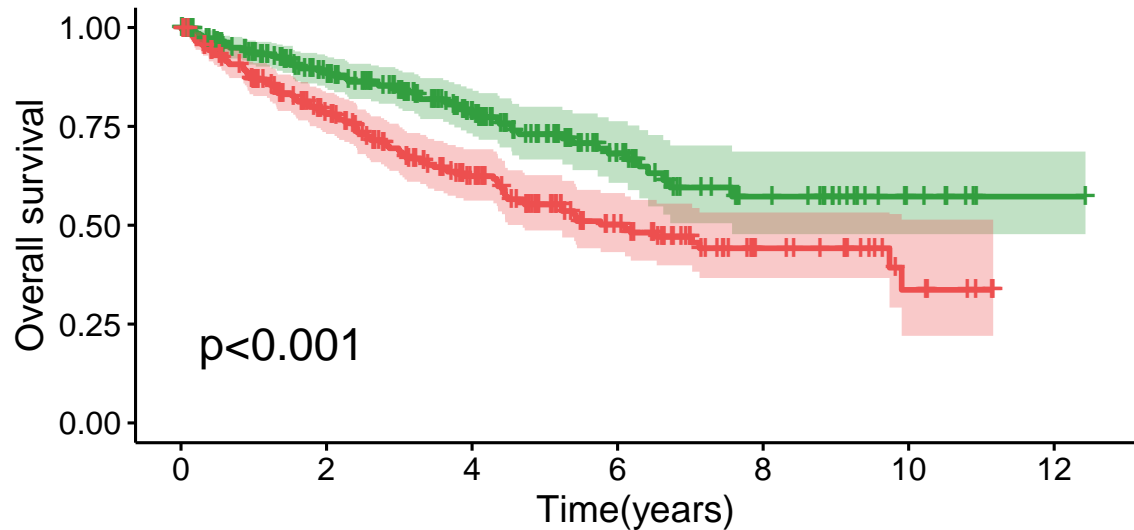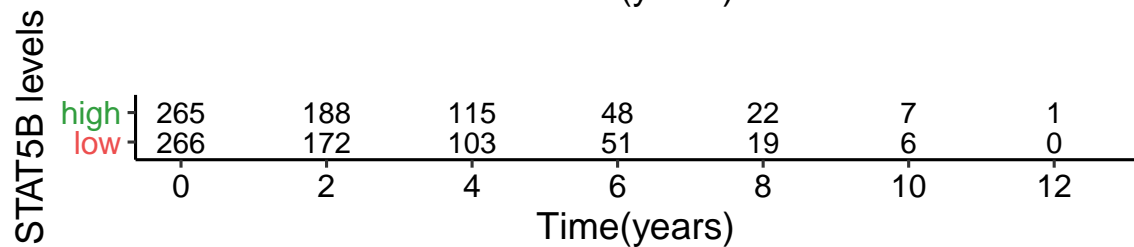

Supplement: Supplementary file 2 [file DataSheet1.ZIP › Source data/STAT5B_KIRC.pdf]

# Cancer: KIRP

STAT5B levels + high + low

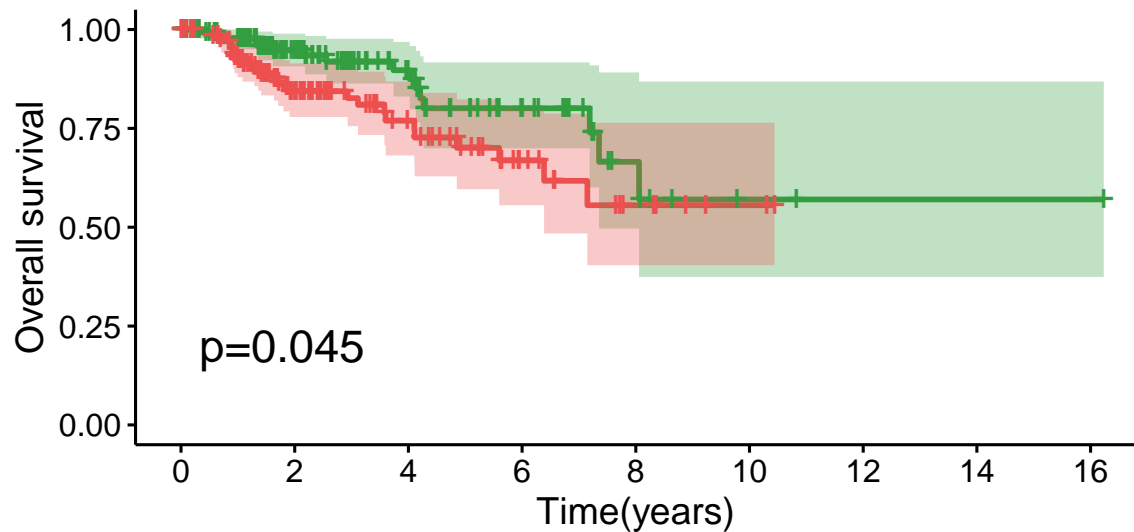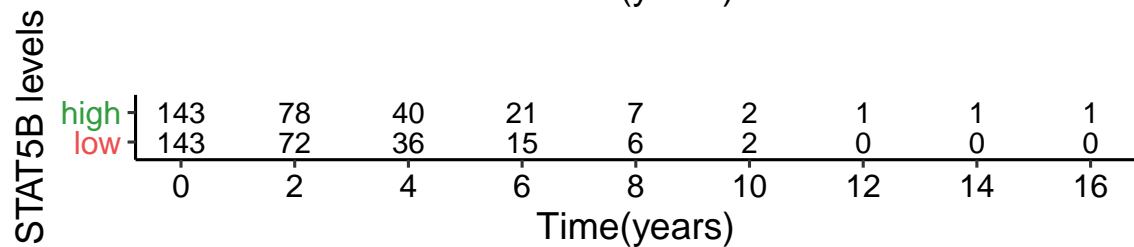

Supplement: Supplementary file 2 [file DataSheet1.ZIP › Source data/STAT5B_KIRP.pdf]

# Cancer: LAML

STAT5B levels + high + low

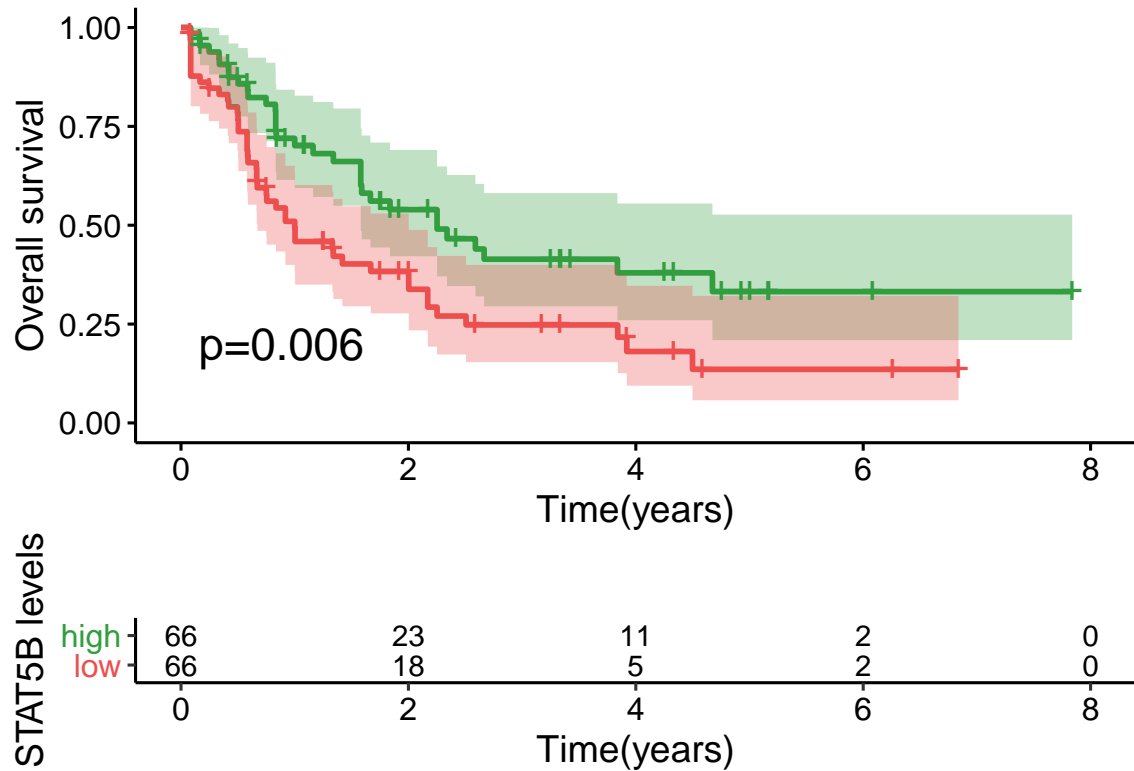

Supplement: Supplementary file 2 [file DataSheet1.ZIP › Source data/STAT5B_LAML.pdf]

# Cancer: PAAD

STAT5B levels + high + low

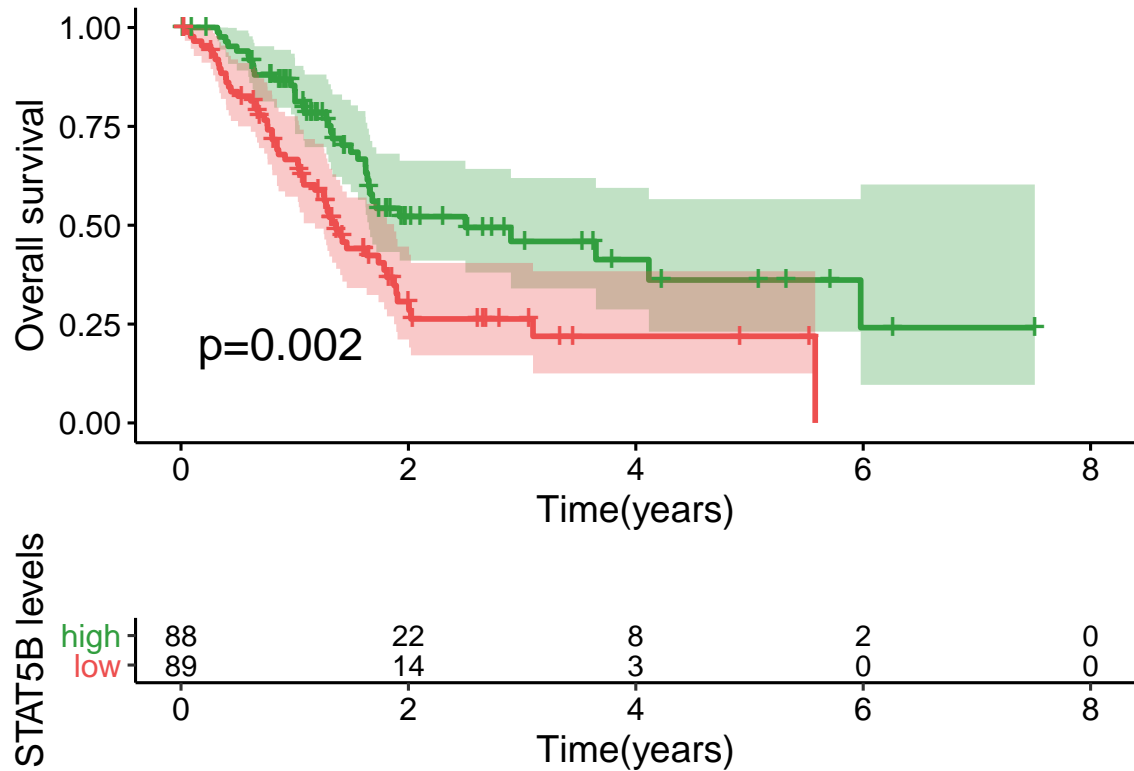

Supplement: Supplementary file 2 [file DataSheet1.ZIP › Source data/STAT5B_PAAD.pdf]

# Cancer: SKCM

STAT5B levels + high + low

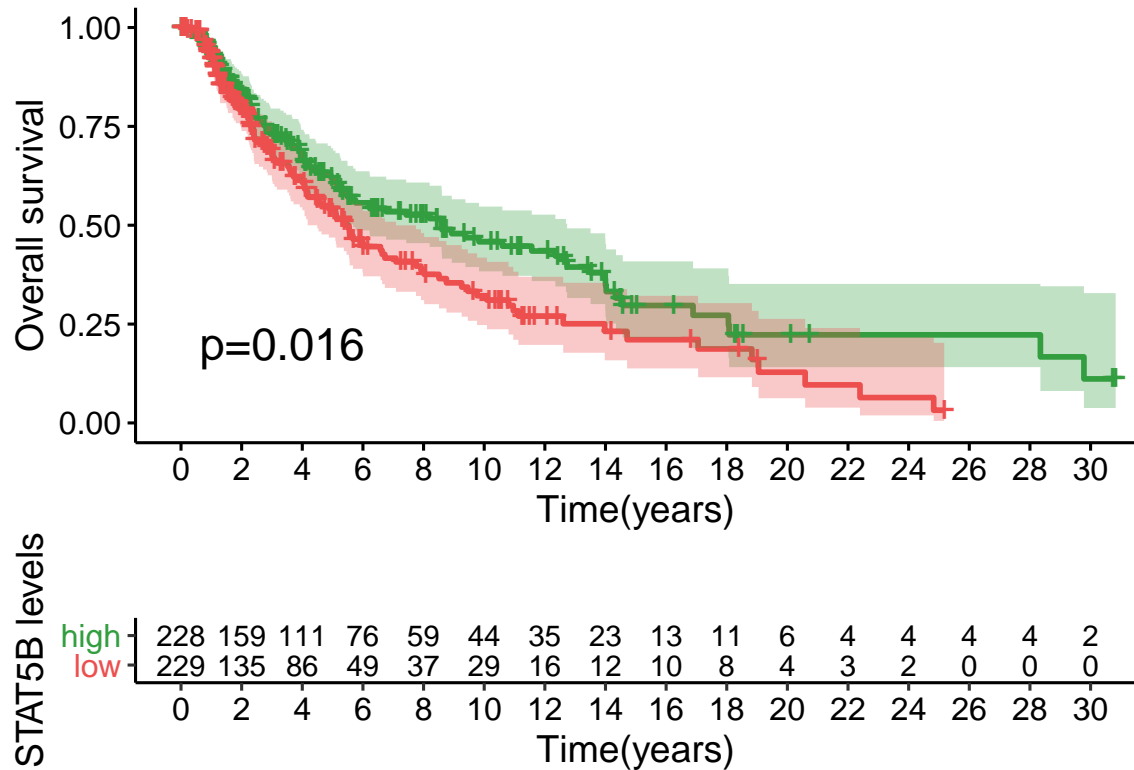

Supplement: Supplementary file 2 [file DataSheet1.ZIP › Source data/STAT5B_SKCM.pdf]

Type 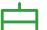 Normal 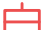 Tumor

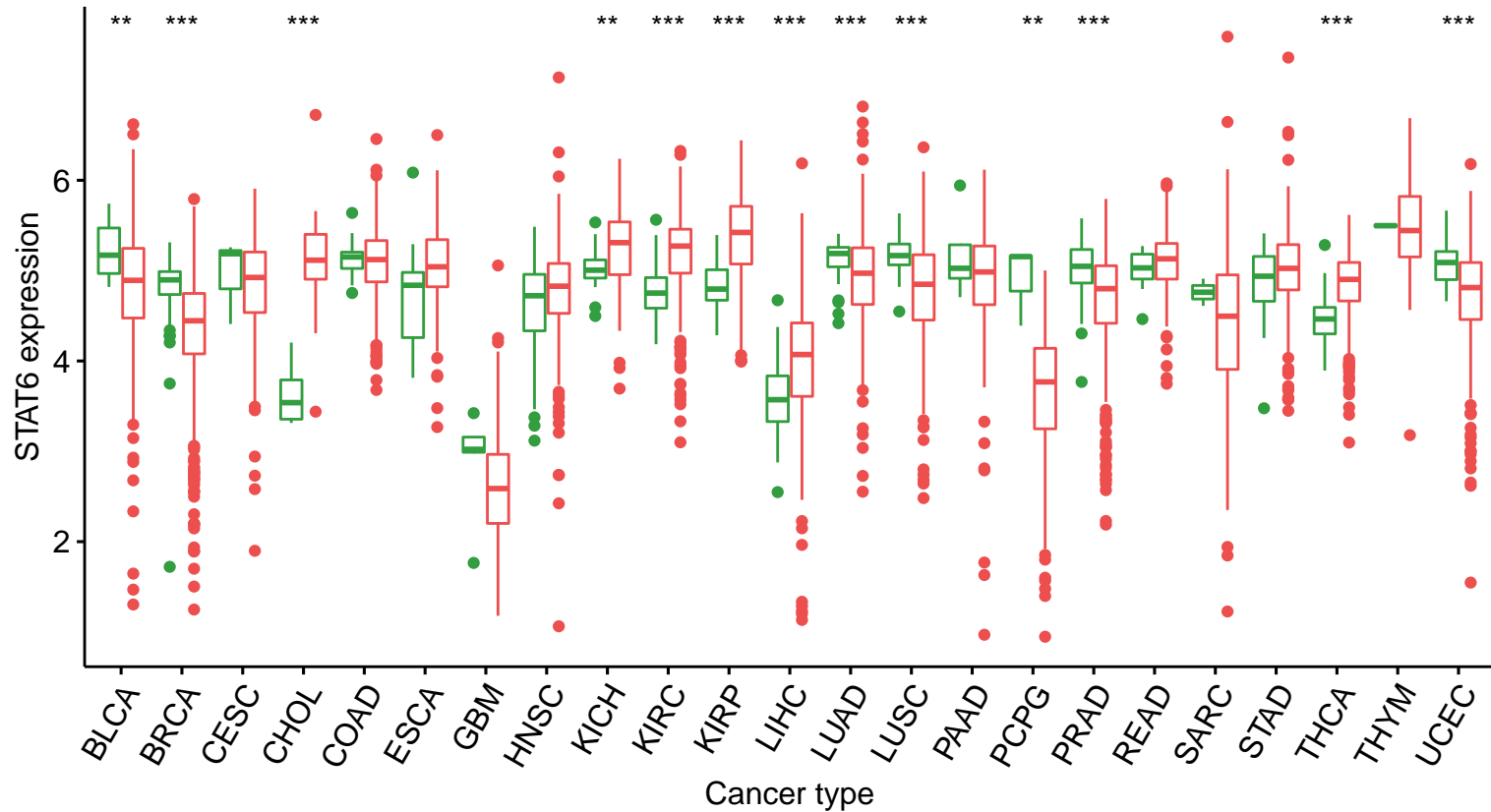

Supplement: Supplementary file 2 [file DataSheet1.ZIP › Source data/STAT6.diff.pdf]

# Cancer: BLCA

STAT6 levels + high + low

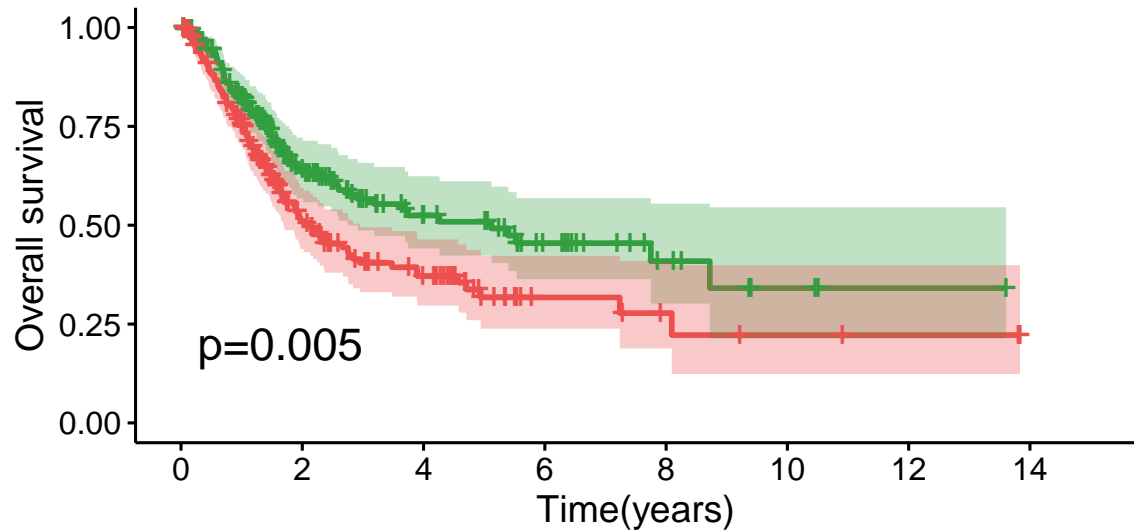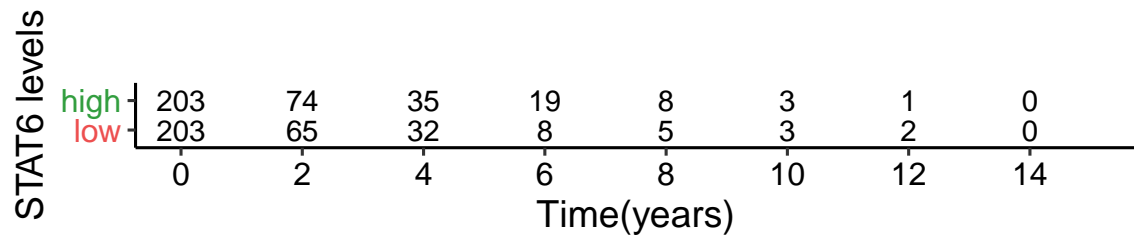

Supplement: Supplementary file 2 [file DataSheet1.ZIP › Source data/STAT6_BLCA.pdf]

# Cancer: GBM

STAT6 levels + high + low

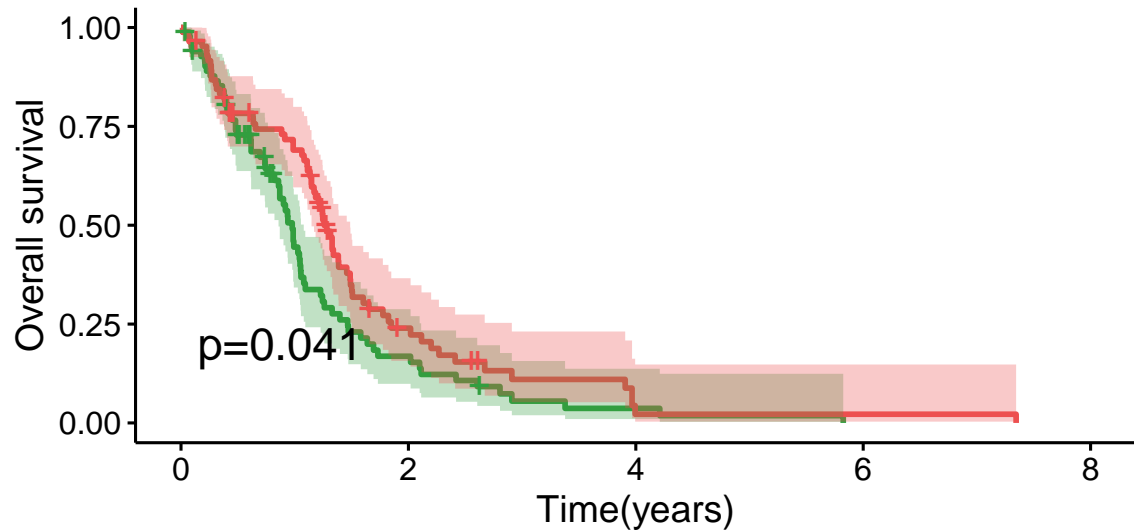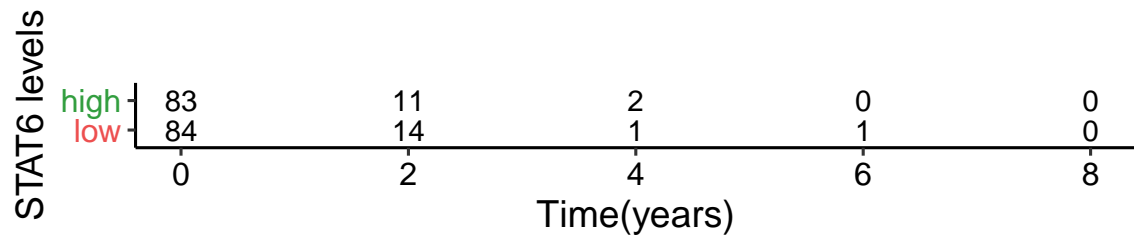

Supplement: Supplementary file 2 [file DataSheet1.ZIP › Source data/STAT6_GBM.pdf]

# Cancer: LAML

STAT6 levels + high + low

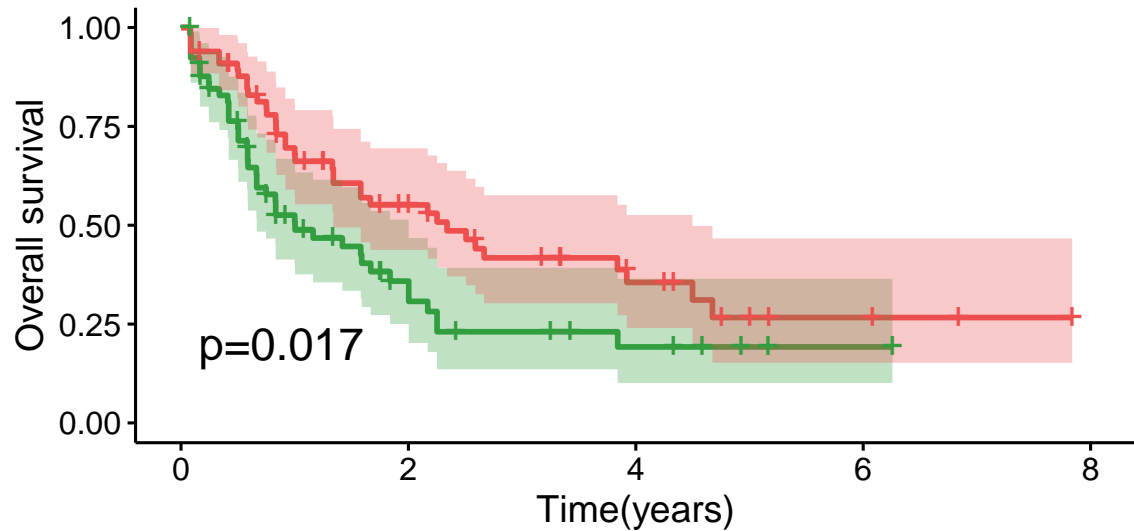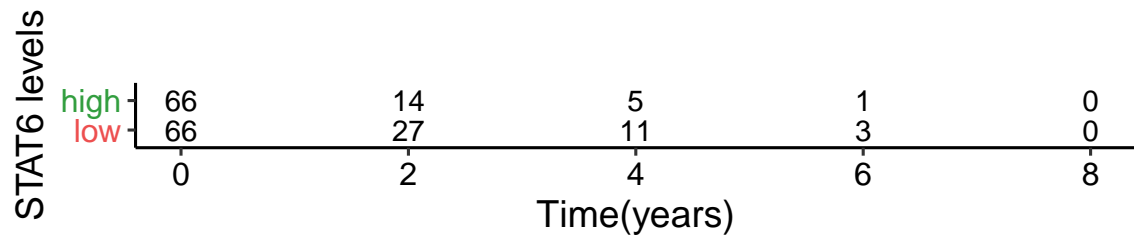

Supplement: Supplementary file 2 [file DataSheet1.ZIP › Source data/STAT6_LAML.pdf]

# Cancer: LGG

STAT6 levels + high + low

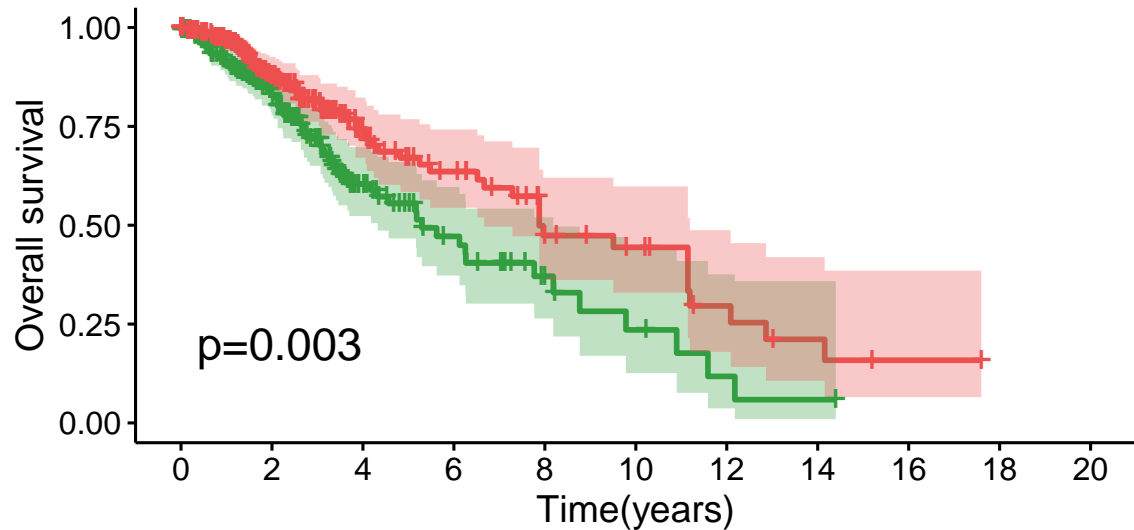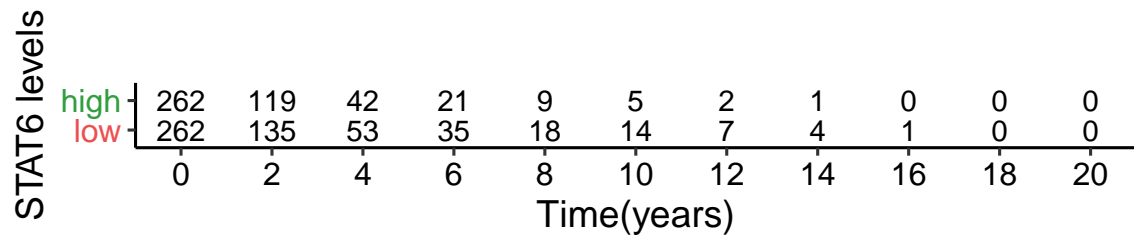

Supplement: Supplementary file 2 [file DataSheet1.ZIP › Source data/STAT6_LGG.pdf]

# Cancer: SARC

STAT6 levels + high + low

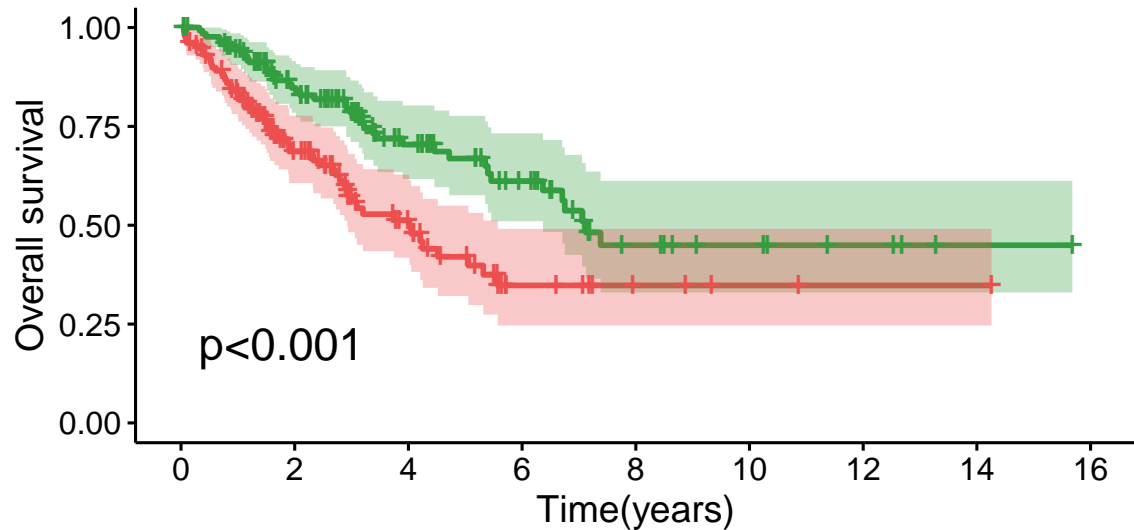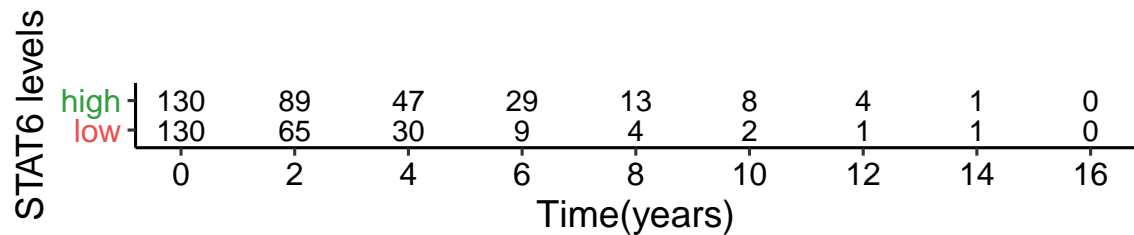

Supplement: Supplementary file 2 [file DataSheet1.ZIP › Source data/STAT6_SARC.pdf]

# Cancer: THCA

STAT6 levels + high + low

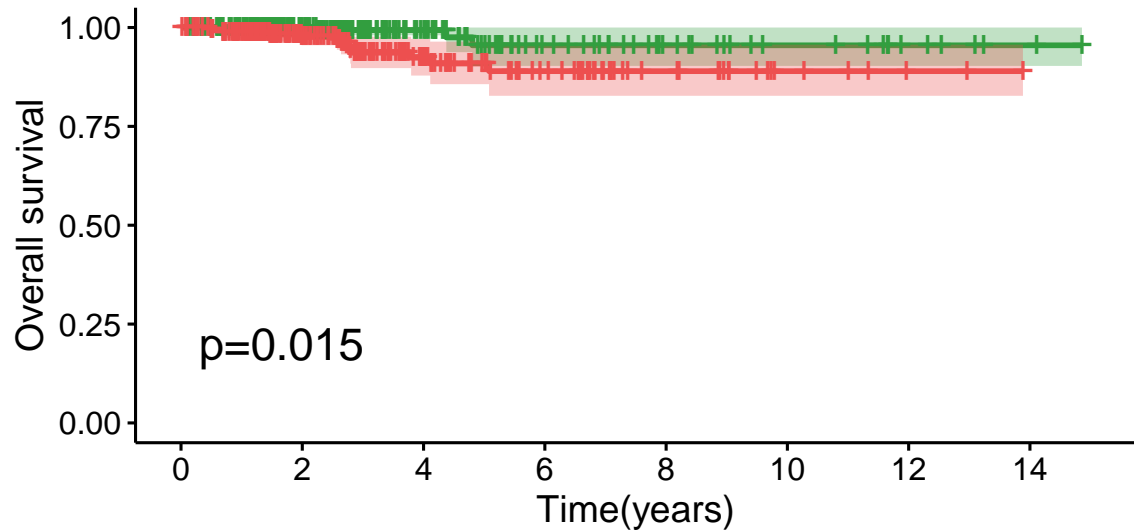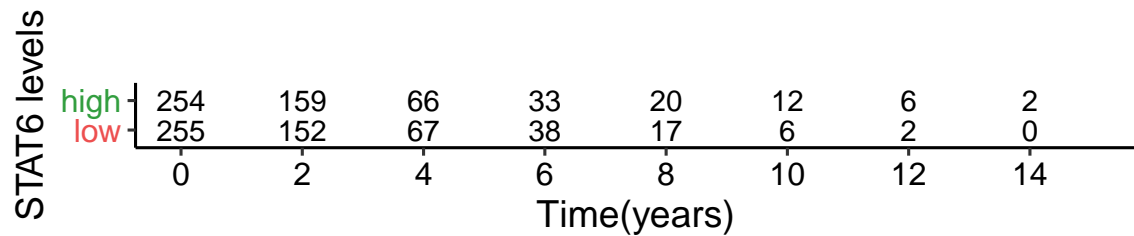

Supplement: Supplementary file 2 [file DataSheet1.ZIP › Source data/STAT6_THCA.pdf]

# Cancer: UCEC

STAT6 levels + high + low

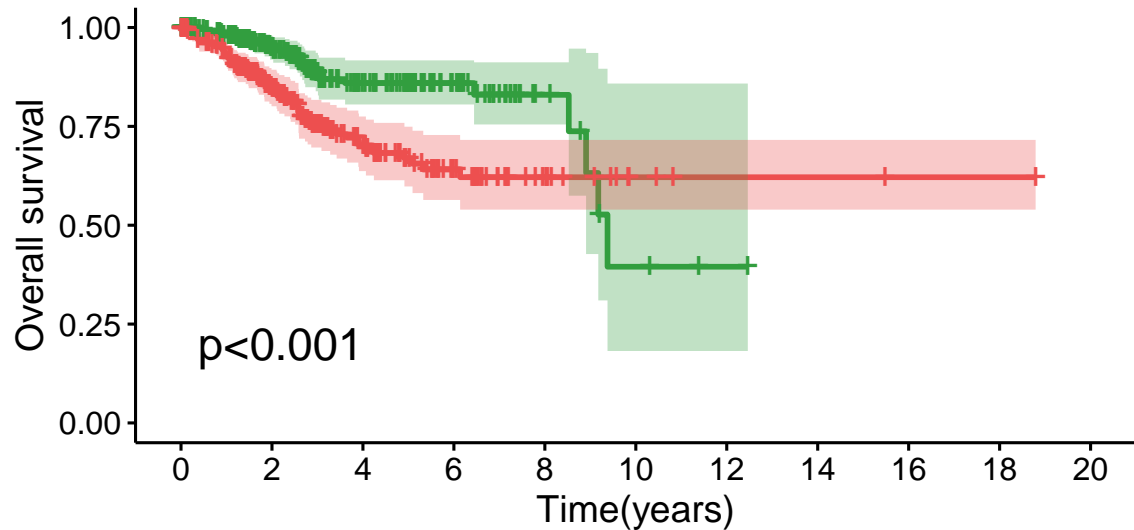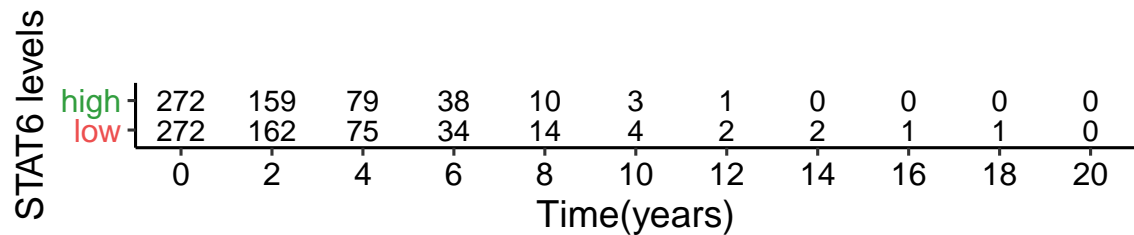

Supplement: Supplementary file 2 [file DataSheet1.ZIP › Source data/STAT6_UCEC.pdf]

# Cancer: UVM

STAT6 levels + high + low

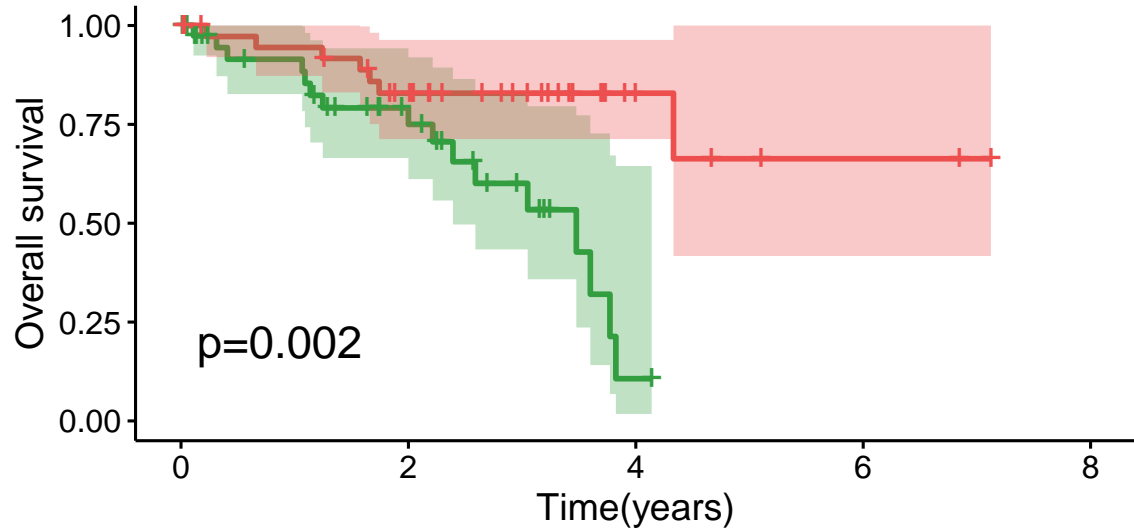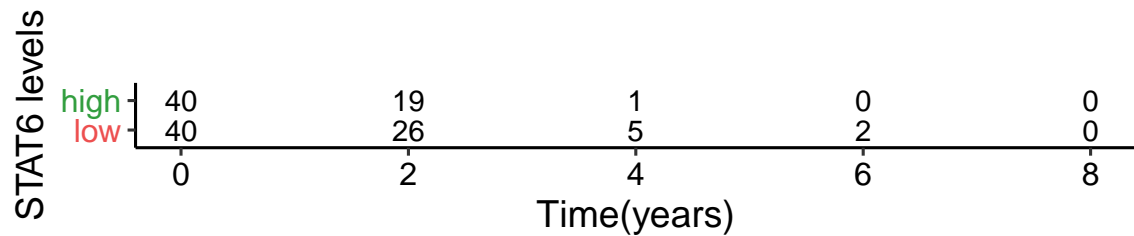

Supplement: Supplementary file 2 [file DataSheet1.ZIP › Source data/STAT6_UVM.pdf]

## StromalScore

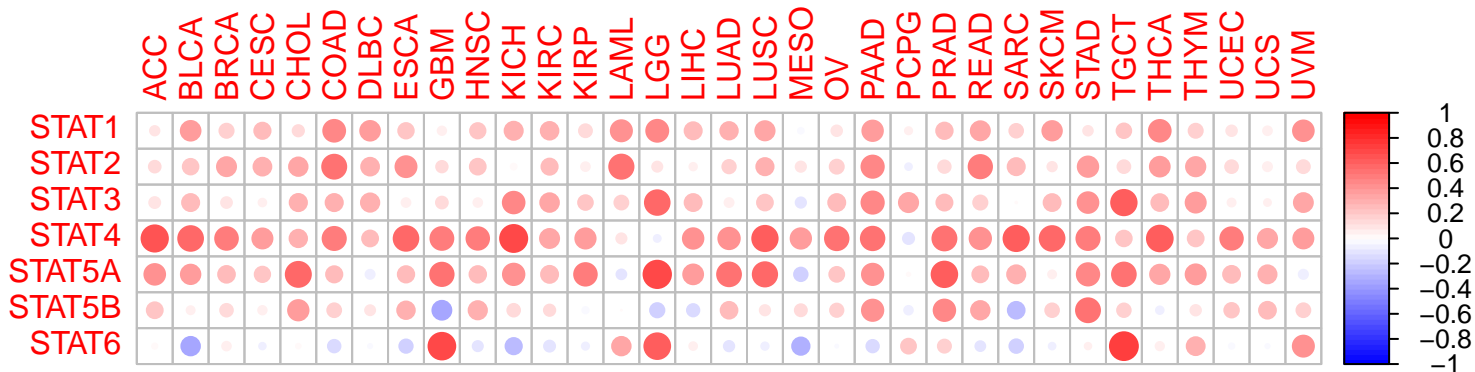

Supplement: Supplementary file 2 [file DataSheet1.ZIP › Source data/StromalScore.pdf]

# TMB

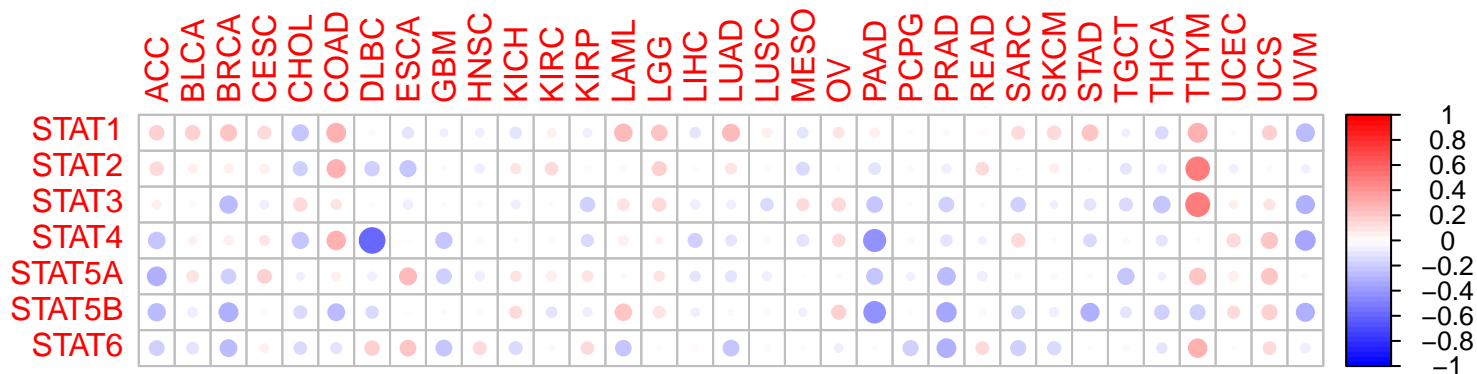

Supplement: Supplementary file 2 [file DataSheet1.ZIP › Source data/TMBCor.pdf]

## TumorPurity

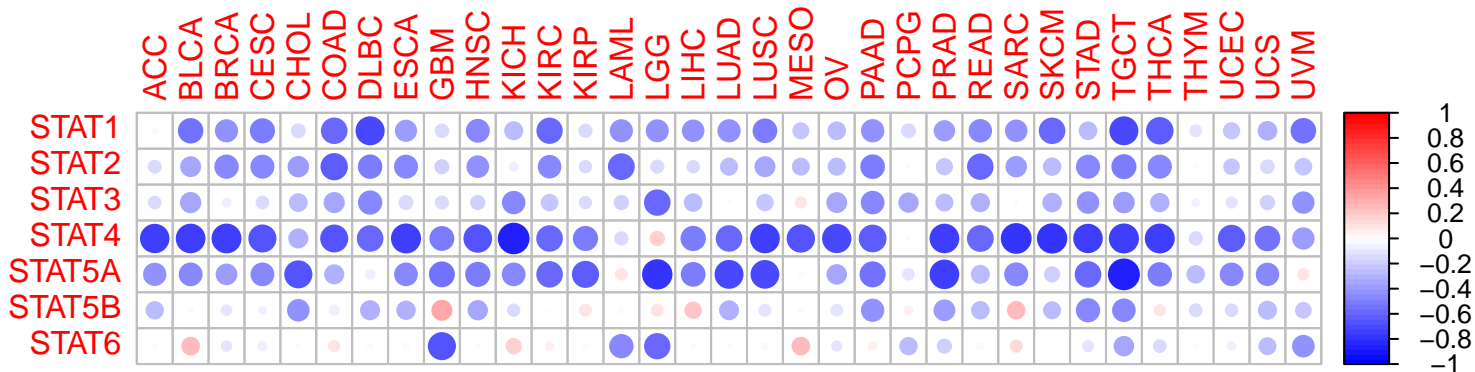

Supplement: Supplementary file 2 [file DataSheet1.ZIP › Source data/TumorPurity.pdf]

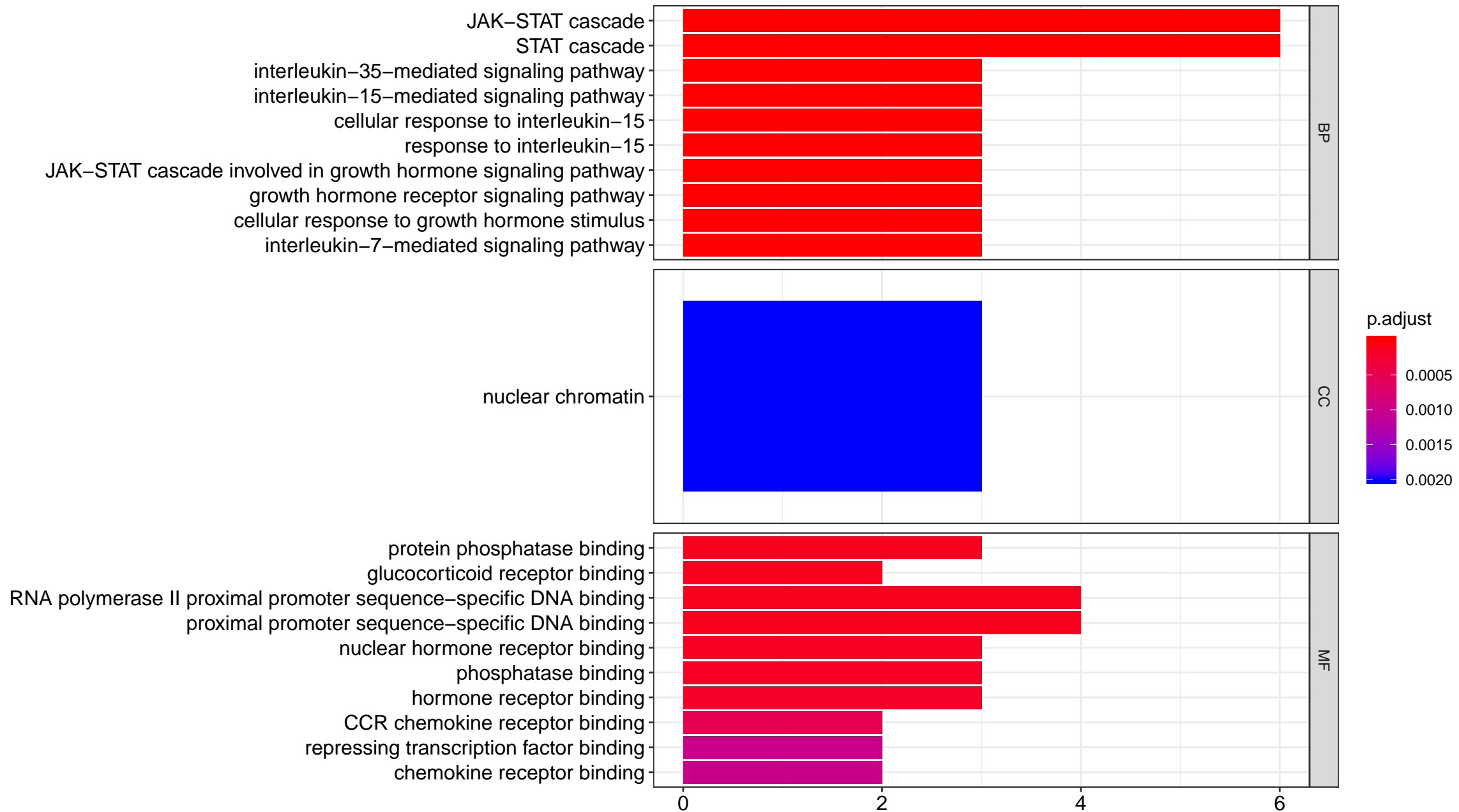

Supplement: Supplementary file 2 [file DataSheet1.ZIP › Source data/alteration/GO_barplot_keygene.pdf]

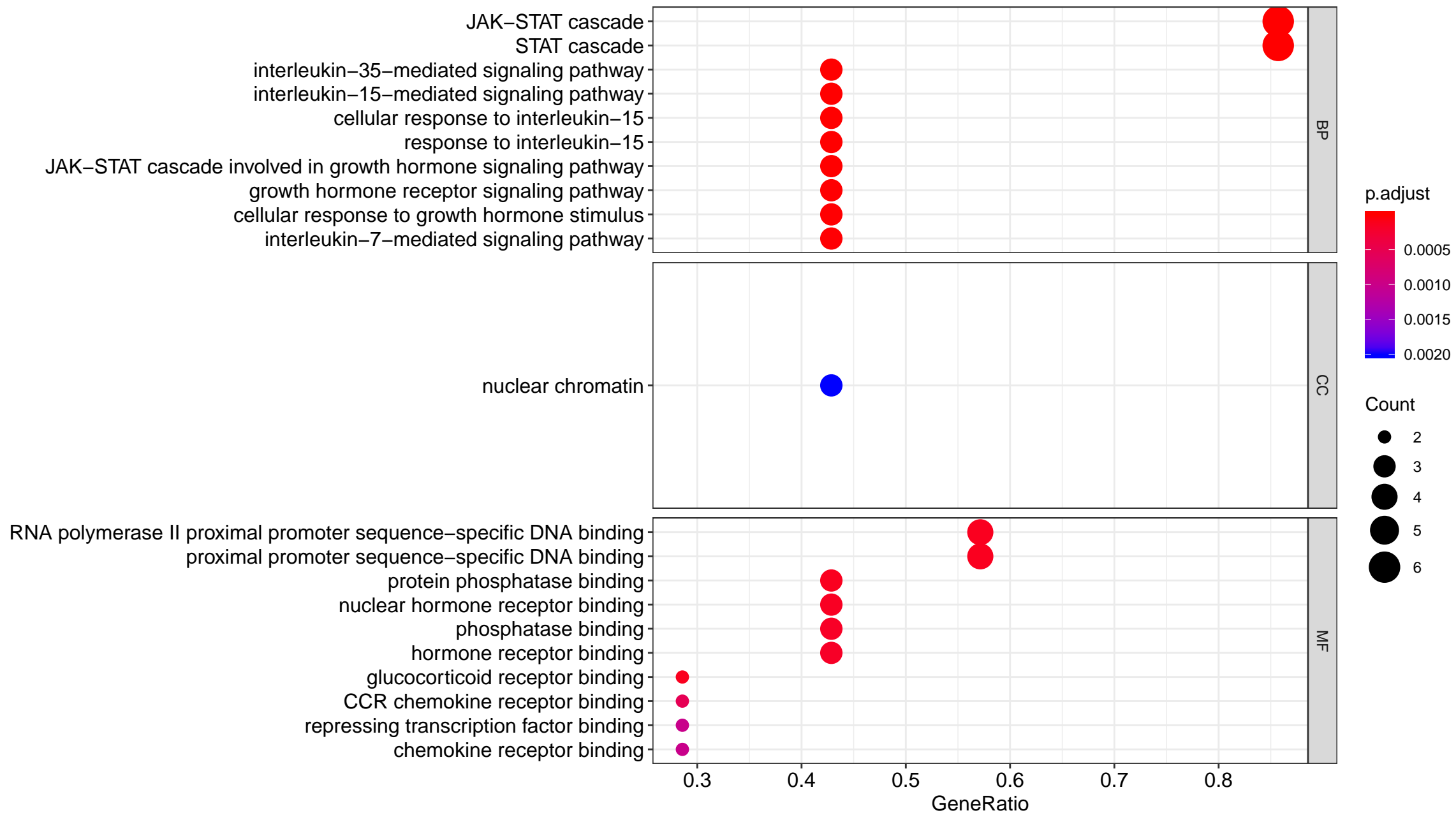

Supplement: Supplementary file 2 [file DataSheet1.ZIP › Source data/alteration/GO_dotplot_keygene.pdf]

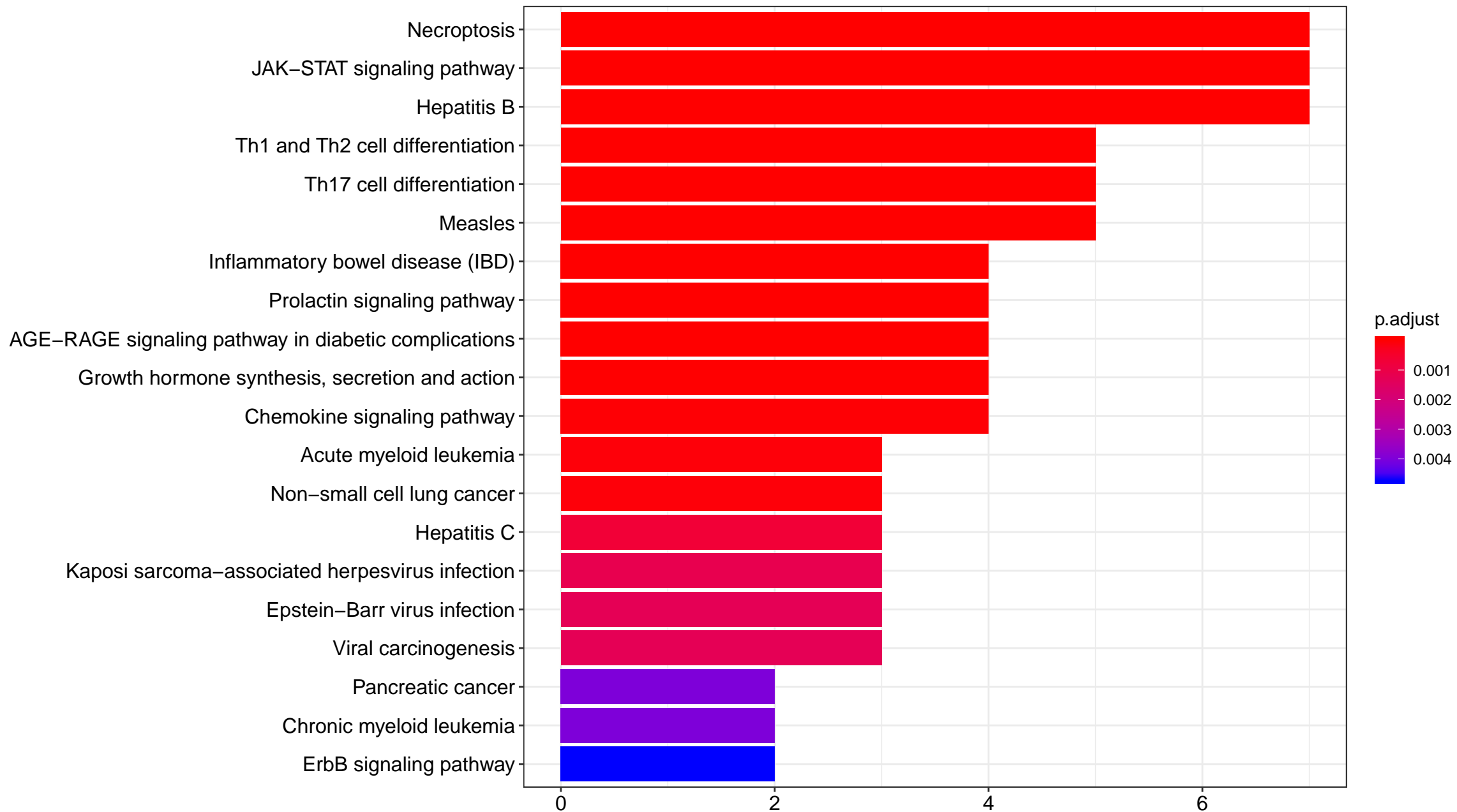

Supplement: Supplementary file 2 [file DataSheet1.ZIP › Source data/alteration/KEGG_barplot_keygene.pdf]

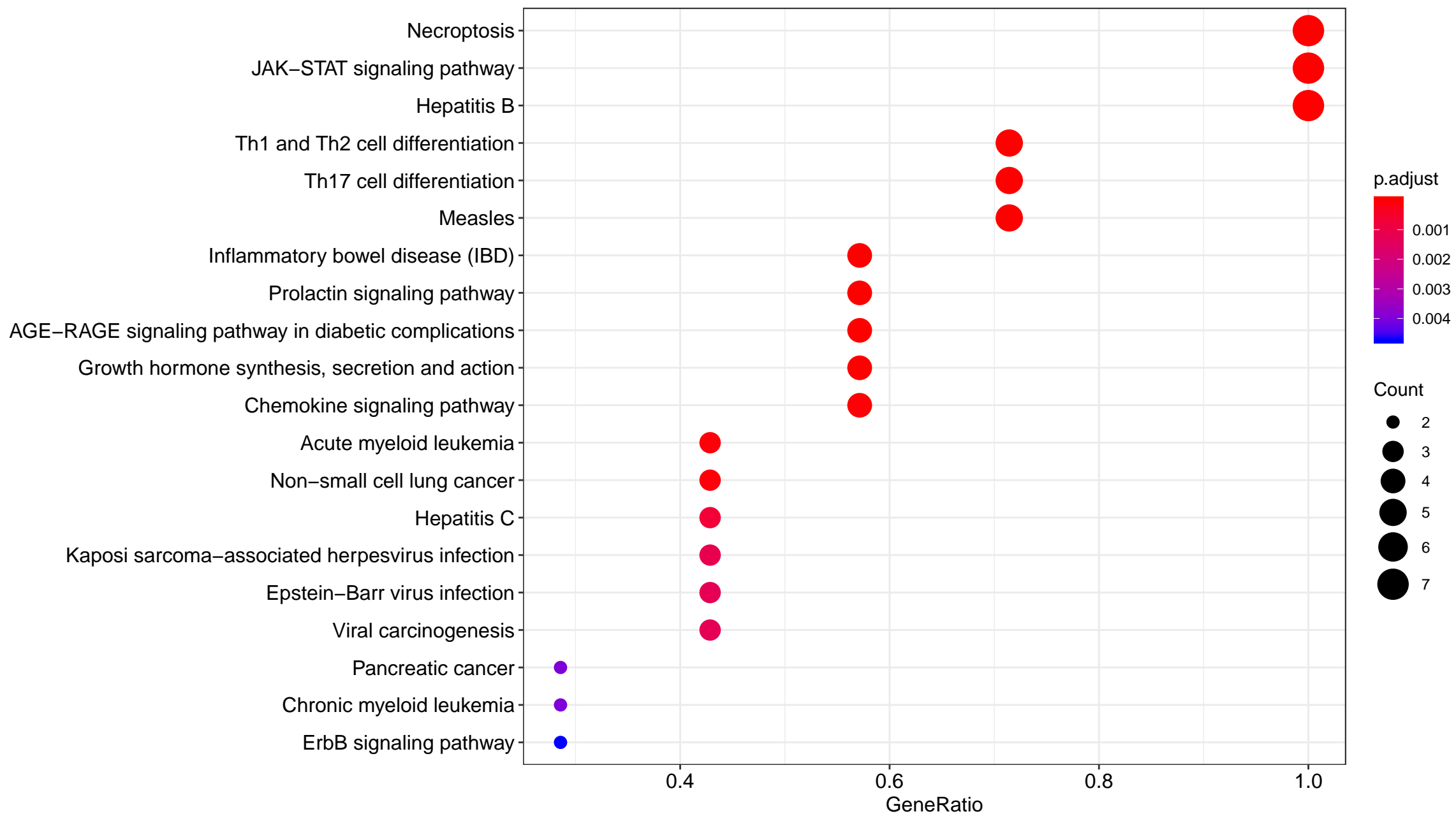

Supplement: Supplementary file 2 [file DataSheet1.ZIP › Source data/alteration/KEGG_dotplot_keygene.pdf]

$\log_{10}(\text{TMB}+1)$

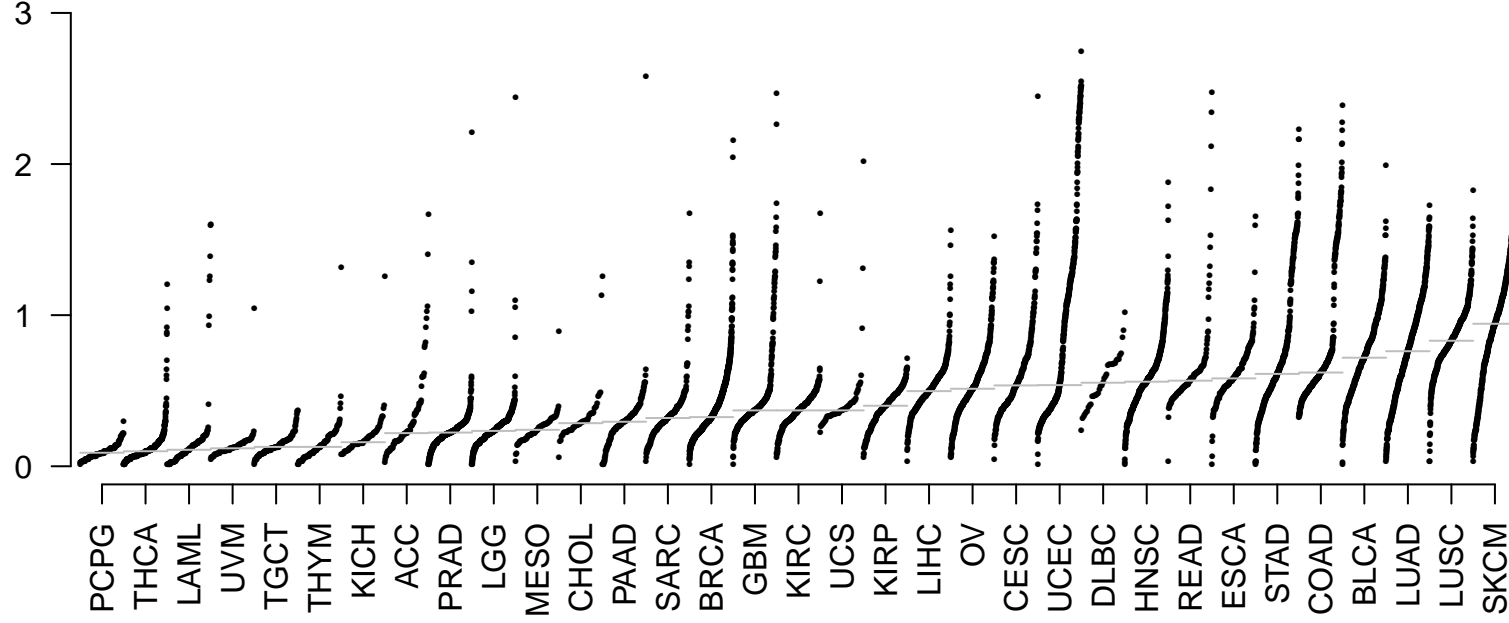

C>T  
T>C  
C>A  
C>G  
T>A  
T>G

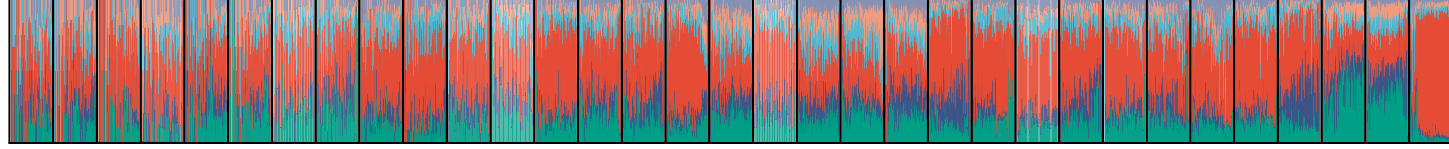

Supplement: Supplementary file 2 [file DataSheet1.ZIP › Source data/alteration/TMB_titv.pdf]

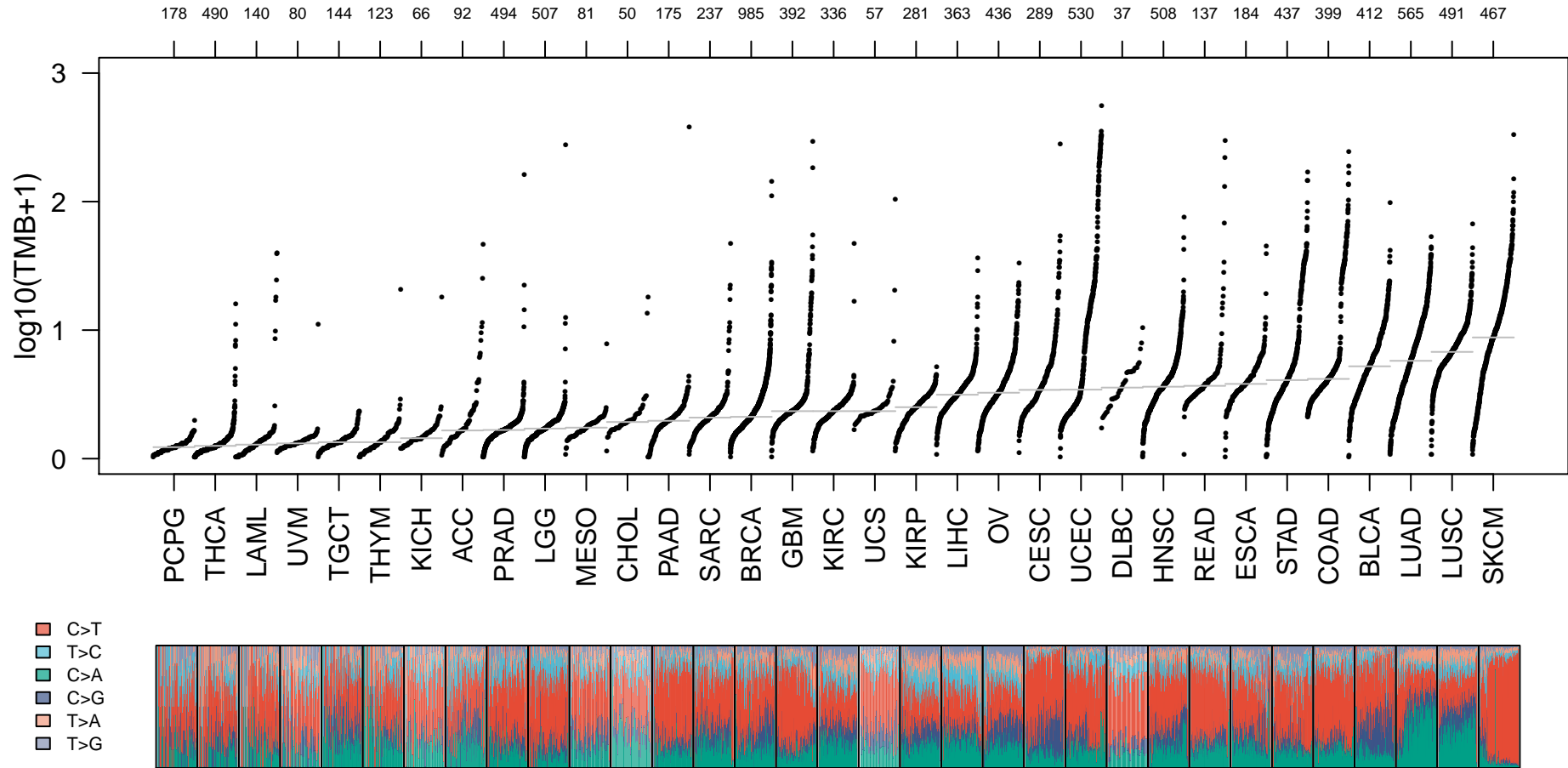

Supplement: Supplementary file 2 [file DataSheet1.ZIP › Source data/alteration/TMB_titv2.pdf]

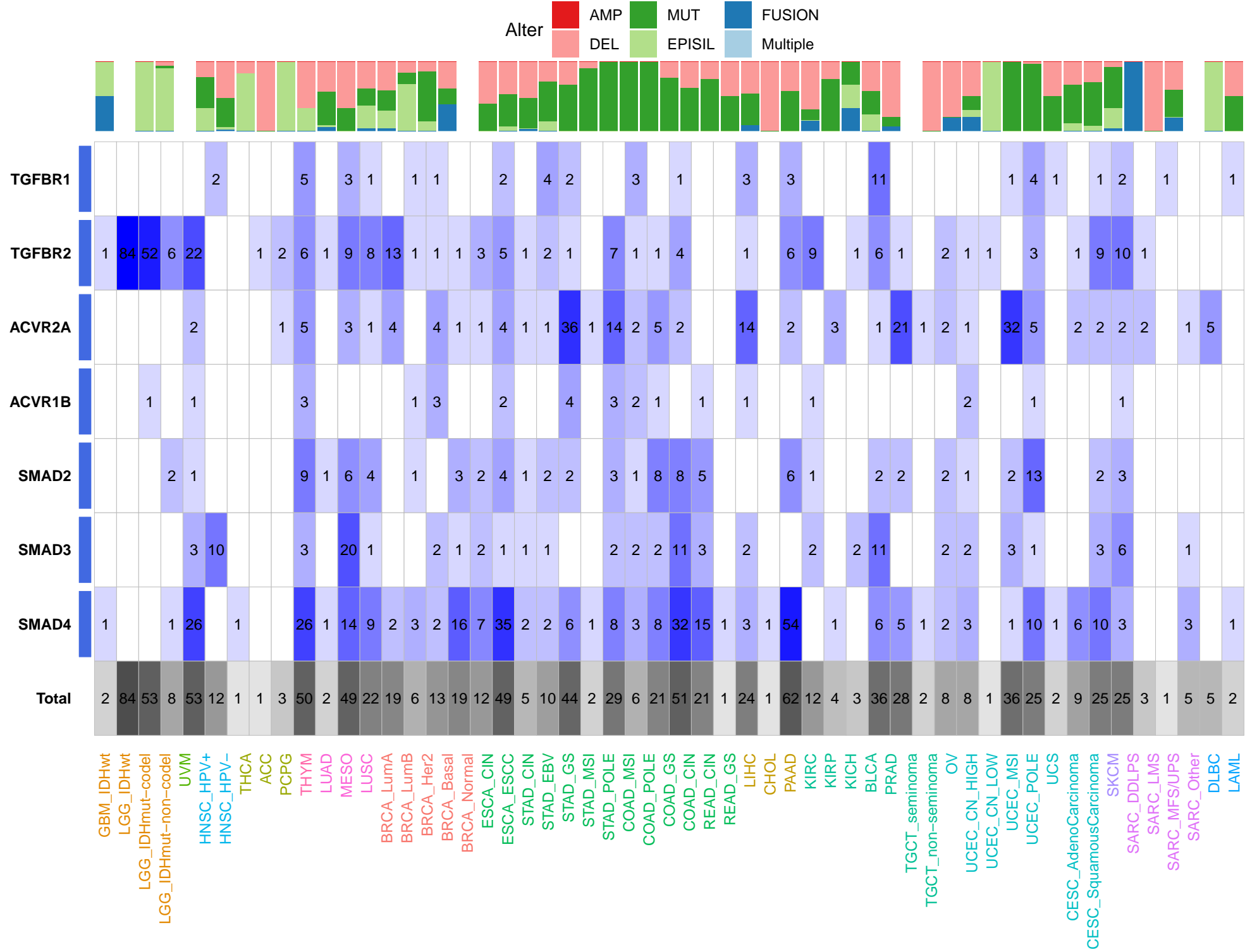

Supplement: Supplementary file 2 [file DataSheet1.ZIP › Source data/alteration/alteration.pdf]

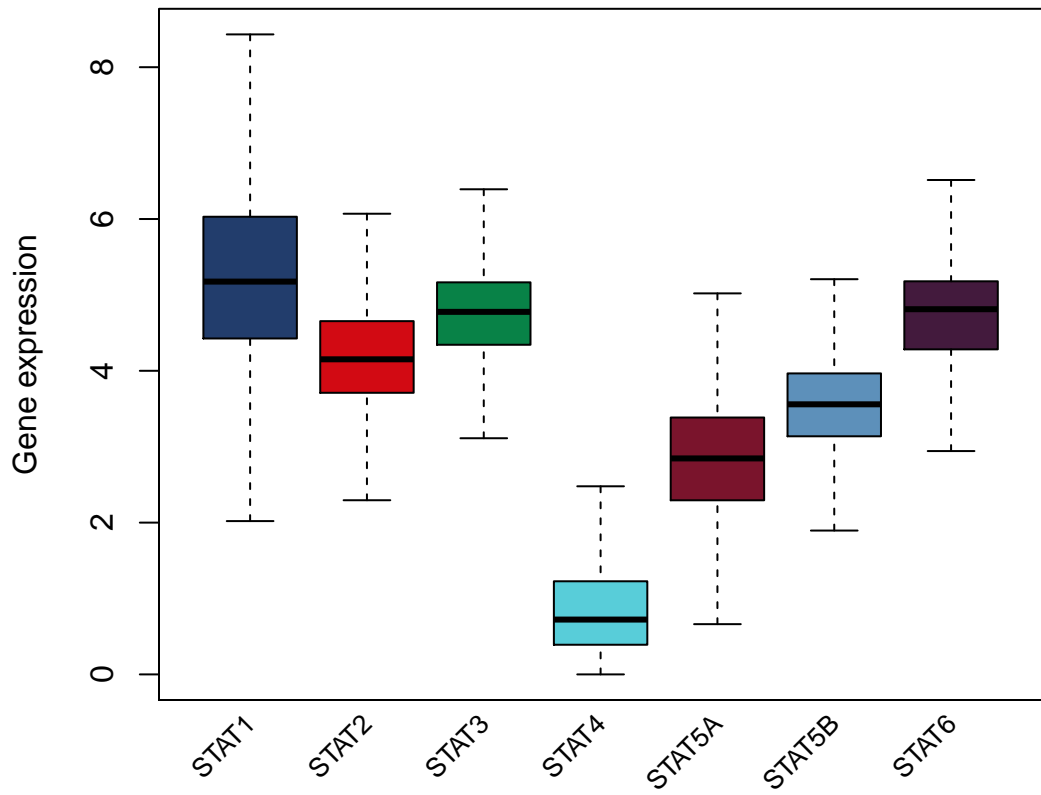

Supplement: Supplementary file 2 [file DataSheet1.ZIP › Source data/boxplot.pdf]

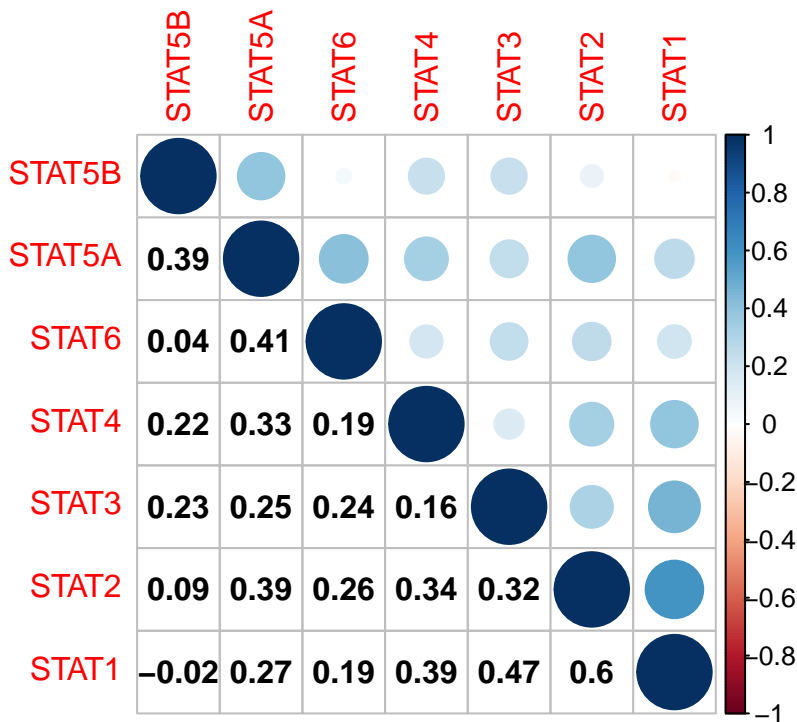

Supplement: Supplementary file 2 [file DataSheet1.ZIP › Source data/corrplot.pdf]

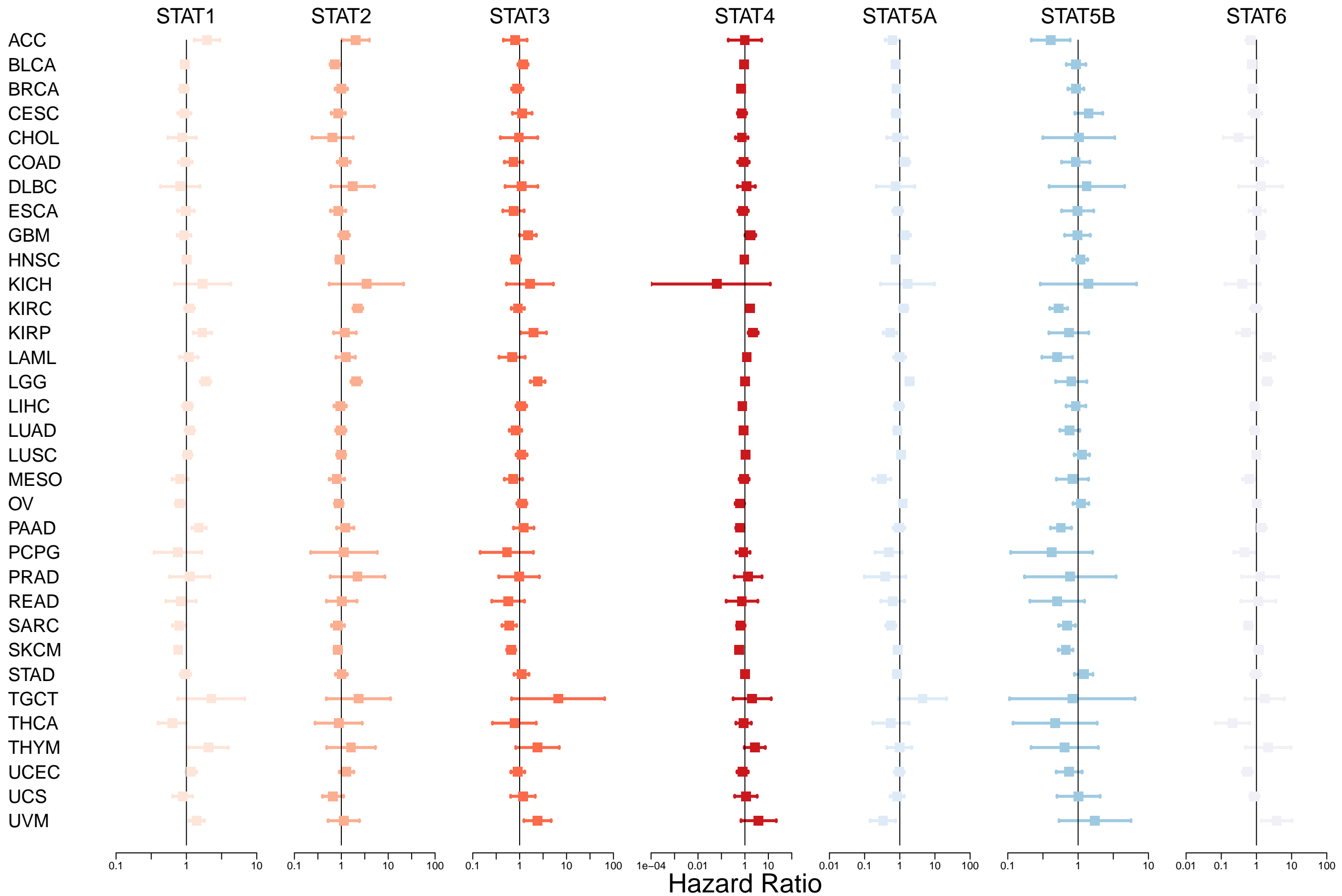

Supplement: Supplementary file 2 [file DataSheet1.ZIP › Source data/forest.pdf]

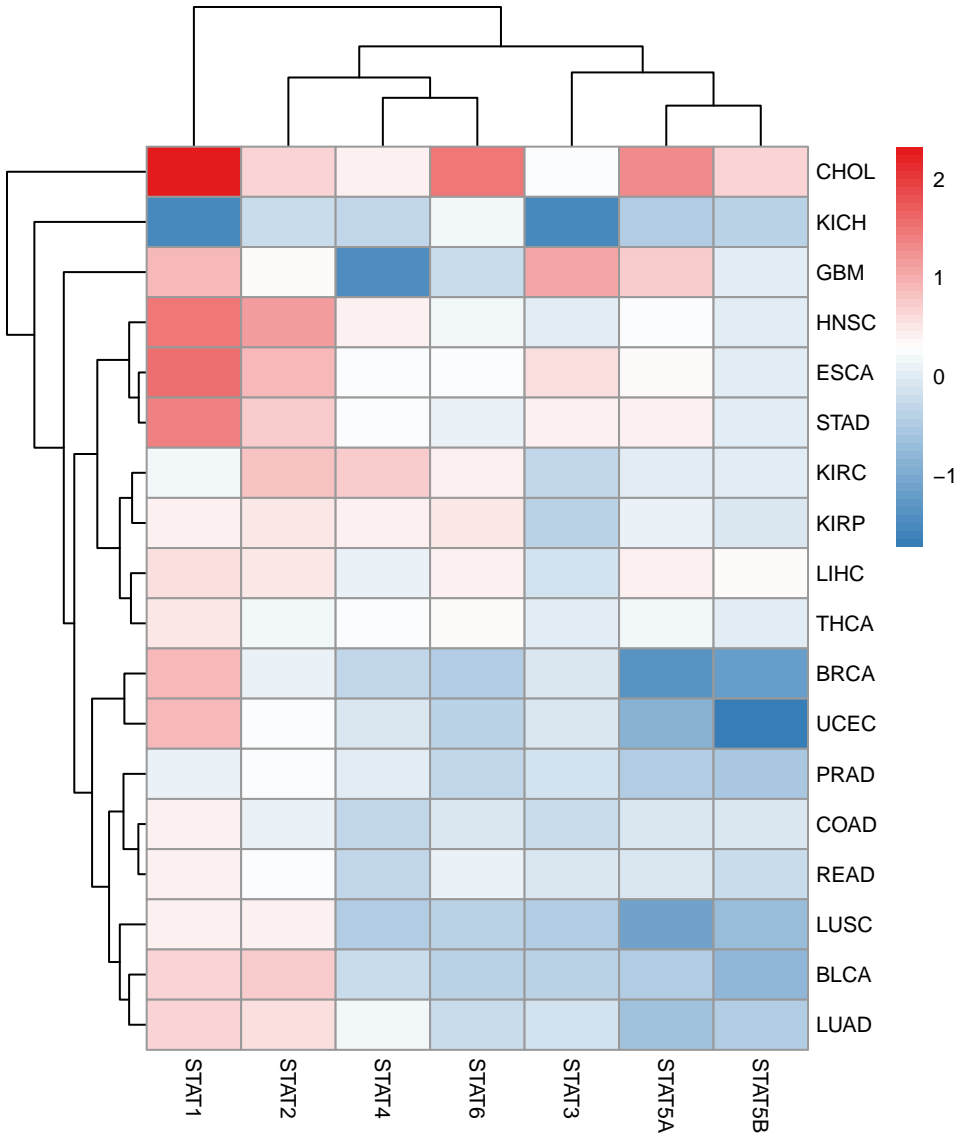

Supplement: Supplementary file 2 [file DataSheet1.ZIP › Source data/heatmap.pdf]

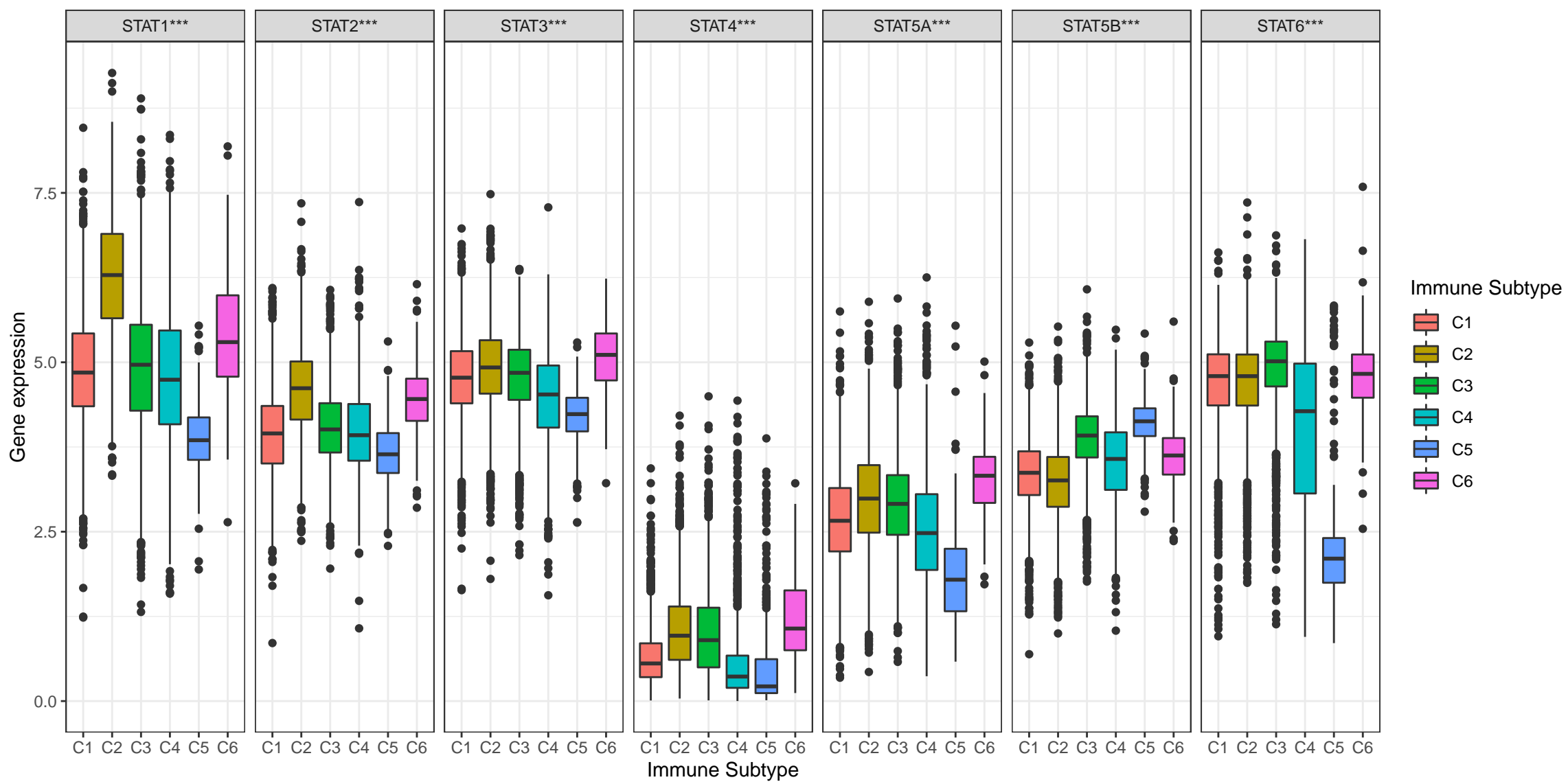

Supplement: Supplementary file 2 [file DataSheet1.ZIP › Source data/immuneType.pdf]
